# Microglia‐Targeted Biomimetic Tetrahedral Framework Nucleic Acid Nanovesicles for Synergistic Treatment of Sepsis‐Associated Encephalopathy

**DOI:** 10.1002/advs.202523716

**Published:** 2026-04-27

**Authors:** Huimin Shi, Qiuxia Gao, Wenying Wang, Bin Li, Yukun Chen, Zhijun Yao, Yujie Li, Junrui Li, Na Li, Gong Gu, Zhimin Hou, Mengyuan Yang, Ruilin Zhang, Hongju Yang, Yuhui Liao, Hongyi Lei

**Affiliations:** ^1^ Department of Anesthesiology Shenzhen Clinical College (Longgang Central Hospital of Shenzhen) Guangzhou University of Chinese Medicine Shenzhen Guangdong China; ^2^ Institute for Engineering Medicine Kunming Medical University Kunming Yunnan China; ^3^ School of Inspection Ningxia Medical University Yinchuan Ningxia China; ^4^ Department of Anesthesiology Southwest Hospital Third Military Medical University (Army Medical University) Chongqing China; ^5^ Institute for Engineering Medicine NHC Key Laboratory of Drug Addiction Medicine Kunming Medical University Kunming Yunnan China; ^6^ Geriatric Medical Center Division of Geriatric Gastroenterology The First Affiliated Hospital of Kunming Medical University Kunming Yunnan China

**Keywords:** anti‐pyroptosis and anti‐inflammatory, cognitive dysfunction, microglia targeting, sepsis‐associated encephalopathy, tetrahedral framework nucleic acid

## Abstract

Sepsis‐associated encephalopathy (SAE), the most prevalent and severe complication of sepsis, is a leading cause of long‐term cognitive deficits and increased mortality. Although anti‐inflammatory and antioxidant therapies have advanced, single‐target drugs cannot disrupt the complex inflammatory cascade in SAE. Therefore, multi‐target synergistic strategies are urgently needed. This study developed a multifunctional biomimetic nanodrug, ME@FDsi, for precise SAE therapy. The system uses a tetrahedral framework nucleic acid (tFNA) as a carrier, connected via base complementary pairing with small interfering RNA (siTNFα) to target TNF‐α. It is also loaded with disulfiram (DSF) to inhibit pyroptosis. The resulting FDsi was encapsulated in erythrocyte membrane vesicles modified with the M1 microglia‐targeting MG1 peptide. ME@FDsi exhibits a nanovesicle structure, prolonged circulation, stability, and biocompatibility. In SAE mice, it crosses the compromised blood‐brain barrier and targets M1 microglia via MG1, releasing DSF and siTNF‐α intracellularly. DSF blocks pyroptosis and IL‐1β release, while siTNFα silences TNF‐α expression. Additionally, tFNA scavenges reactive oxygen species. Together, these actions shift microglia from the M1 to the M2 phenotype. ME@FDsi treatment improved cognitive function, reduced multi‐organ damage, and increased survival in SAE mice. This multi‐mechanism synergistic approach offers a promising therapeutic strategy for clinical SAE and sepsis.

## Graphical abstract

Sepsis‐associated encephalopathy (SAE) lacks effective therapies. We developed ME@FDsi, a biomimetic nanodrug using a tetrahedral framework nucleic acid to deliver disulfiram and siTNFα. It crosses the blood‐brain barrier, targets M1 microglia, inhibits pyroptosis and inflammation, and scavenges ROS. ME@FDsi improved cognitive function, reduced organ damage, and increased survival in SAE mice, offering a promising multi‐target therapeutic strategy.

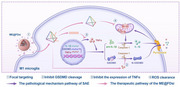

## 1 Introduction

Sepsis‐associated encephalopathy (SAE) is a severe neurological complication triggered by a systemic inflammatory response, with an incidence rate as high as 50%–70% in the intensive care unit (ICU), and is a key factor contributing to poor prognosis in septic patients [1, 2]. Patients primarily present with acute cognitive impairment, which can rapidly progress from mild inattention to lethargy or coma. Studies have confirmed that SAE is not only an independent risk factor for patient mortality but also significantly exacerbates management difficulties and mortality risk within the ICU—its mortality risk is positively correlated with the severity of SAE. Even mild SAE can increase mortality risk by 38%, moderate disease by 80%, and once coma ensues, the mortality risk is as high as 3.37 times that of patients without SAE [3]. More critically, among SAE survivors discharged from the hospital, a considerable proportion experience long‐term cognitive decline, executive dysfunction, psychiatric and behavioral abnormalities, and even persistent disability. These sequelae severely impair their quality of life and social adaptability, imposing a heavy long‐term care burden on families and society [4, 5].

Driven by neuroinflammation and neuronal death, SAE is orchestrated by microglia, which propel a self‐reinforcing vicious cycle. Initially, systemic inflammatory mediators—such as TNF‐α, IL‐1β, IL‐6, endotoxins, reactive oxygen species, and metabolic byproducts—enter the brain tissue through the compromised blood‐brain barrier, directly activating the resident immune cells of the brain: microglia [6]. Once activated, microglia shift toward a pro‐inflammatory phenotype and subsequently produce large quantities of various neurotoxic substances, including the aforementioned cytokines, chemokines, and reactive oxygen species. These substances not only directly exacerbate neuronal damage and synaptic dysfunction and recruit peripheral immune cell infiltration but also induce pyroptosis in microglia. Pyroptosis is a highly inflammatory and lytic form of programmed cell death. Its occurrence leads to plasma membrane rupture and the release of large amounts of potent inflammatory factors such as mature IL‐1β and IL‐18, along with other intracellular contents—akin to detonating an “inflammatory bomb” within the brain. This not only directly damages surrounding neurons but also severely disrupts the integrity of the blood‐brain barrier (BBB) [7, 8]. Subsequently, the inflammatory storm—released by pyroptotic microglia and continuously produced by surviving activated microglia—further activates and recruits additional microglia, thereby triggering more pyroptosis and inflammatory responses. Meanwhile, the persistent disruption of the BBB facilitates the influx of more peripheral inflammatory mediators and immune cells into the brain, establishing a positive feedback loop. Ultimately, this self‐amplifying “inflammation‐damage” cycle leads to severe cerebral dysfunction, leaving survivors at risk of long‐term adverse outcomes such as cognitive impairment and functional disability. Although existing antibiotic therapies can control infection, they do not suppress pro‐inflammatory cytokines and are often associated with risks of secondary seizures, cognitive impairment, and drug resistance [9, 10]. Therefore, there is an urgent need to develop therapeutic agents capable of breaking the vicious cycle of aberrant microglial activation and pyroptosis.

Disulfiram (DSF), a medication used to treat alcohol addiction, has been found to inhibit pyroptosis by suppressing the formation of GSDMD pores, demonstrating significant potential in the treatment of sepsis and SAE [11, 12, 13]. However, DSF faces limitations such as poor water solubility, low bioavailability, and a short half‐life [14]. To overcome these challenges, we introduced a novel drug‐loading system—tetrahedral framework nucleic acid (tFNA)—as a drug carrier for DSF (forming FD). tFNA is a 3D nanostructure that has garnered increasing attention due to its broad applications in drug delivery [15]. Compared to carriers such as liposomes and PLGA/PLA nanoparticles, tFNA offers multiple advantages, including excellent biocompatibility, programmability, and drug‐loading capacity [16, 17, 18]. Previous studies have shown that tFNA can deliver oligonucleotide therapeutics (e.g., small interfering RNA and microRNA), peptides (e.g., antimicrobial peptides), and small‐molecule drugs (e.g., resveratrol and curcumin) [16, 17, 19, 20]. Additionally, research has demonstrated that tFNA exerts immunomodulatory effects both in vitro and in vivo [15, 21, 22]. Specifically, tFNA can inhibit M1 polarization in macrophages and reduce the secretion of inflammatory cytokines and reactive oxygen species [23].

Furthermore, small interfering RNA targeting the pro‐inflammatory cytokine TNF‐α (siTNFα) was precisely loaded onto FD via the principle of base complementary pairing, forming FDsi. This siTNFα can efficiently silence TNF‐α expression at the genetic level, thereby blocking the signaling pathway mediated by this key inflammatory mediator at its source [20, 24, 25, 26]. Subsequently, we employed erythrocyte membrane coating to encapsulate the FDsi‐loaded construct, aiming to prolong its circulation time and enhance bioavailability [27, 28, 29]. To achieve specific targeting of M1‐type microglia, we modified the surface with the MG1 peptide (sequence: CHHSSSAR), which was identified through phage display screening and exhibits high affinity for M1 microglia [30, 31, 32].

**SCHEME 1:**
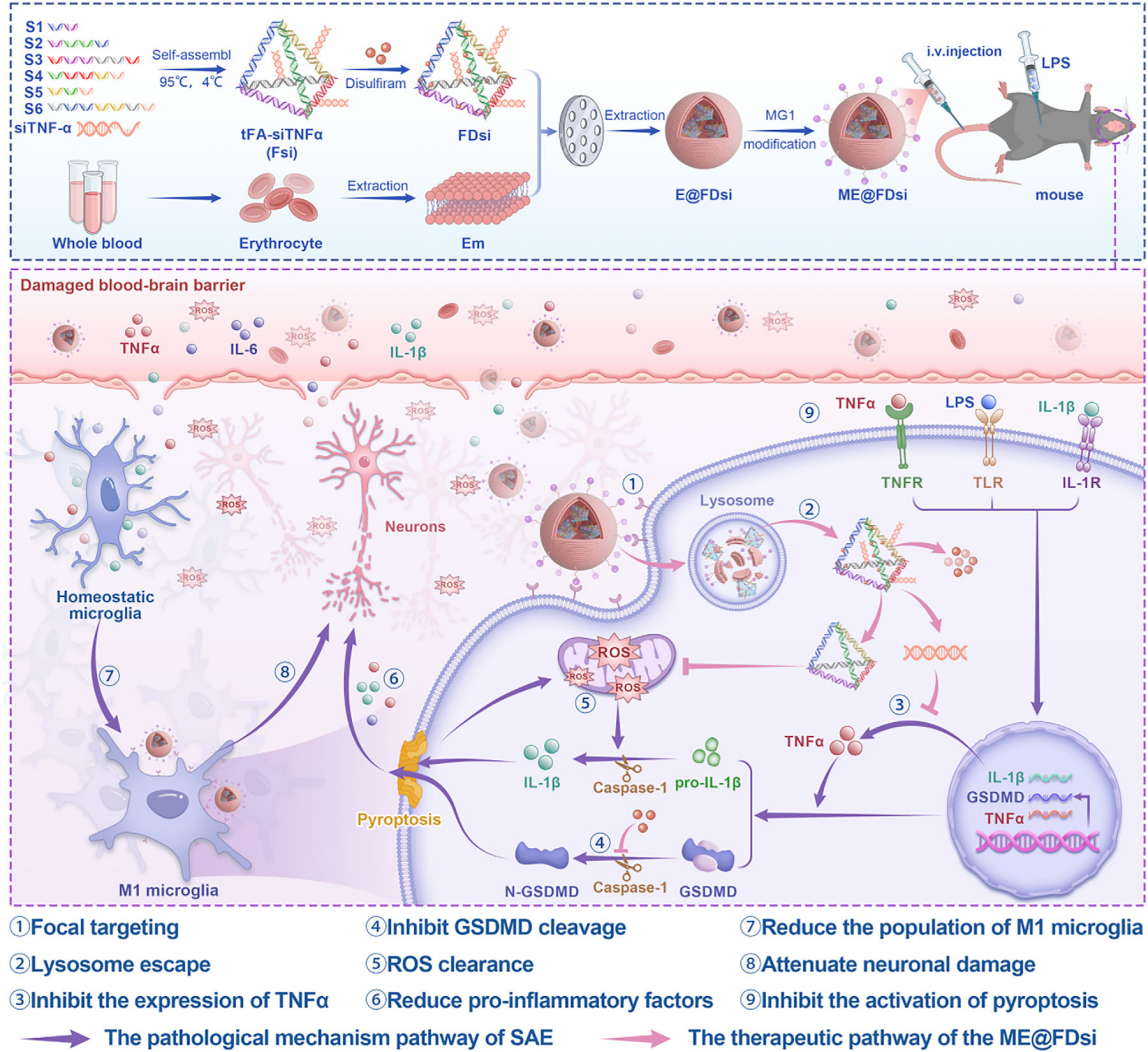
Schematic illustration of the ME@FDsi nanoparticle preparation and its synergistic anti‐pyroptotic/anti‐inflammatory therapeutic effect in an LPS‐induced SAE mouse model.

The ultimately constructed nanosystem, ME@FDsi (Scheme 1), upon intravenous administration, efficiently crosses the compromised blood‐brain barrier and is precisely delivered to activated microglia within the brain. After successful lysosomal escape, the system releases its therapeutic components, effectively breaking the vicious positive feedback cycle driving neuroinflammation through multi‐mechanistic synergistic actions. The core mechanisms are as follows: on one hand, the released disulfiram directly inhibits the cleavage of the pyroptosis execution protein Gasdermin D, thereby blocking the pyroptosis process in microglia. This significantly reduces the release of pro‐inflammatory factors such as IL‐1β, which in turn limits its binding to the IL‐1 receptor and the subsequent activation of the NF‐κB pathway. On the other hand, the Fsi nanostructure simultaneously achieves gene silencing of TNF‐α and scavenging of ROS. This not only reduces the initiation of the NF‐κB pathway at the source by limiting TNF‐α binding to its receptor but also synergistically weakens their role as “second signals” in amplifying the pyroptosis pathway. Consequently, two key upstream pathways that strongly activate and amplify NF‐κB signaling are simultaneously suppressed, depriving this core inflammatory pathway of sustained activation signals. This combined action collectively modulates the immune polarization of microglia. Experimental results confirm that this treatment significantly inhibits neuroinflammation and oxidative stress, reduces the shift of microglia toward a pro‐inflammatory phenotype, and re‐establishes immune homeostasis in the brain. Thereby, it effectively ameliorates SAE‐related neurological impairment and cognitive deficits in animal models, improves survival rates, and reduces systemic inflammation and organ damage (Scheme 1). Given that sepsis and SAE are promoted by the synergy of multiple inflammatory mediators, and targeting a single mediator fails to disrupt the inflammatory cascade, the ME@FDsi‐based nanodrug demonstrates significant potential for multi‐mechanism synergistic therapy, offering a novel nanotherapeutic strategy for SAE.

## 2 Results

### 2.1 Synthesis and Characterization of ME@FDsi

**FIGURE 1:**
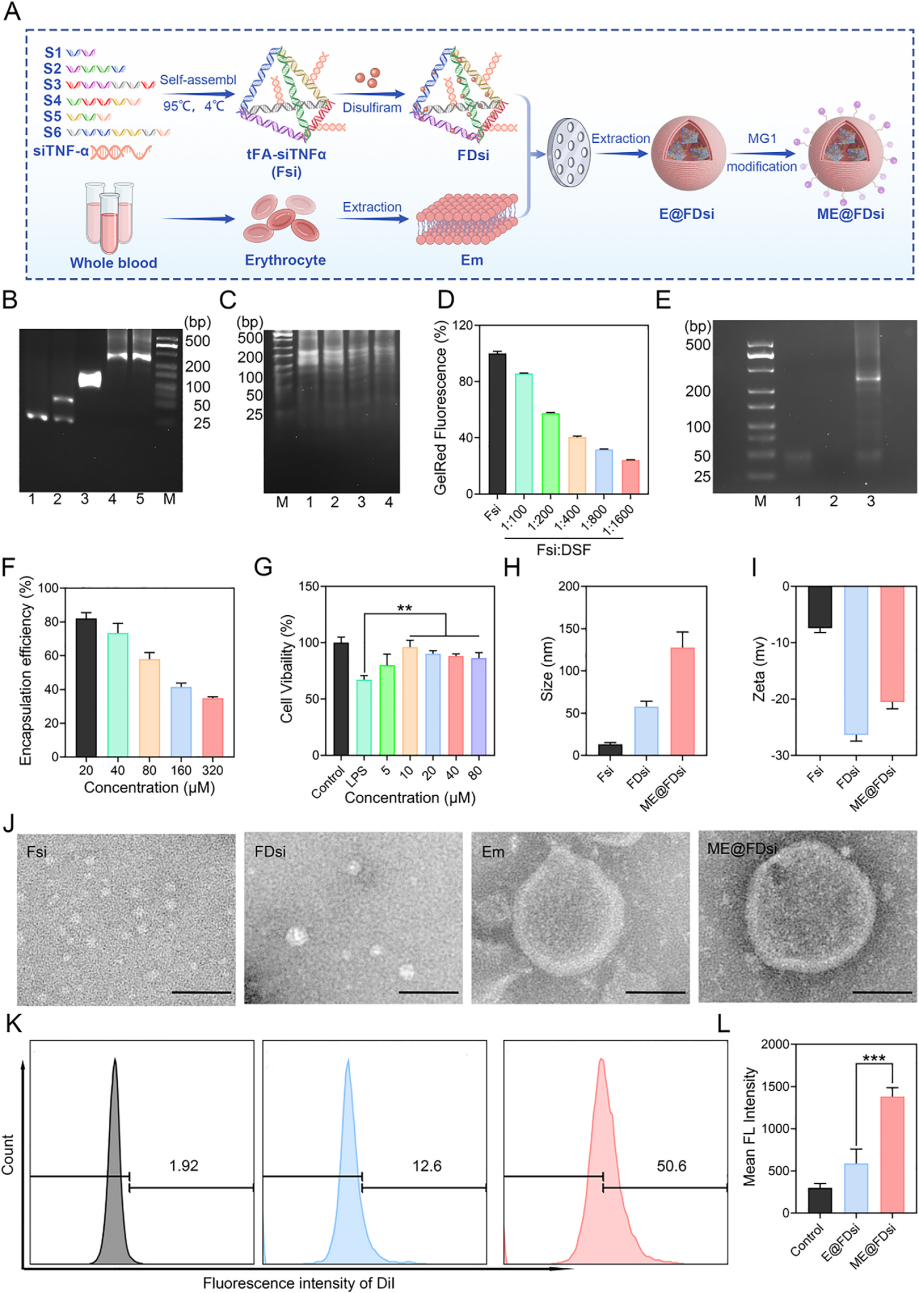
Synthesis and characterization of FDsi and ME@FDsi. (A) Schematic diagram of the synthesis of ME@FDsi. (B) Confirmation of successful Fsi synthesis via agarose gel electrophoresis. Lane 1, S1; Lane 2, S1+S2; Lane 3, S1 + S2 + S3; Lane 4, S1 + S2 + S3 + S4 + S5 + S6 (tFNA); Lane 5, tFNA+siTNF‐α (Fsi); M, Marker. (C) Confirmation of successful FDsi synthesis via agarose gel electrophoresis. M, Marker; Lane 1, Fsi; Lane 2, Fsi:DSF = 1:100; Lane 3, Fsi:DSF = 1:200; Lane 4, Fsi:DSF = 1:400. (D) The Gel‐Red fluorescence intensity ratio (λex = 312 nm) of FDsi to Fsi at different DSF ratios (Fsi:DSF = 1:100–1600). (E) Stability analysis of Fsi. Dissociation of Fsi after incubation with serum at 37°C was detected by agarose gel electrophoresis. M, Marker; Lane 1, siTNF‐α control without incubation; Lane 2, siTNF‐α incubated in serum for 2 h; Lane 3, Fsi incubated in serum for 2 h. (F) Encapsulation efficiency curve of DSF loaded onto Fsi with increasing DSF concentration (n = 3). (G) Cell viability of BV2 cells after LPS stimulation and treatment with different concentrations of ME@FDsi for 24 h. (H) Molecular sizes of Fsi, FDsi, and ME@FDsi determined by DLS. (I) Zeta potentials of Fsi, FDsi, and ME@FDsi measured by DLS. (J) TEM images of Fsi, FDsi, membrane, and ME@FDsi (Scale bar: 50 nm). (K and L) Uptake of Dil‐labeled ME@FDsi compared to FDsi encapsulated by RBC membranes without conjugated MG1 peptide. Uptake in LPS‐treated BV2 cells was detected by flow cytometry. Data are presented as mean ± standard deviation (SD) (n = 3). Statistical significance was calculated by one‐way ANOVA. ^*^ *p* < 0.05, ^**^ *p* < 0.01, ^***^ *p* < 0.001, n.s. indicates no significant difference.

tFNA‐siTNF‐α (Fsi) was prepared by self‐assembling short‐chain DNA fragments and therapeutic siTNF‐α (specific components detailed in Table S1). Successful synthesis of Fsi was confirmed by agarose gel electrophoresis (Figure 1B). Fsi was then mixed with various concentrations of DSF (20, 40, 80, 160 µm) for 6 h. The formation of Fsi‐DSF (FDsi) was verified by gel electrophoresis (Figure 1C), which showed a gradual band shift with increasing DSF concentration, indicating successful drug loading. Existing studies indicate that small molecules can interact with DNA through an intercalative binding mode [33]. To confirm this binding mode, free Fsi and FDsi were stained with GelRed, a dye that binds to nucleic acid grooves. The FDsi bands became progressively fainter than those of free Fsi (Figure 1C), suggesting that DSF displaced GelRed from the DNA [34]. To further confirm the binding site of DSF, a GelRed fluorescence competition assay was performed. FDsi was prepared at various Fsi/DSF ratios, stained with GelRed, and its fluorescence intensity (λex = 312 nm) was measured. The intensity decreased as DSF concentration increased (Figure 1D), confirming that DSF competes with GelRed for the same intercalative binding sites on DNA. To assess the stability of Fsi containing siTNF‐α against enzymatic degradation, the DNA nanostructure was incubated in an environment containing fetal bovine serum (FBS). Agarose gel electrophoresis analysis showed that unprotected free siRNA was almost completely degraded after 2 h of incubation, while the siRNA assembled within Fsi showed a released siTNF‐α band (Figure 1E), indicating that Fsi effectively protects siTNF‐α from enzymatic degradation. To determine the drug loading ratio in Fsi, the encapsulation efficiency at different component ratios was measured. With Fsi fixed at 200 nm, the encapsulation efficiency of DSF decreased as DSF concentration increased (Figure 1F). At a ratio of 40 µm DSF to 200 nm Fsi, the encapsulation efficiency reached 73.43 ± 5.73%, and cell viability under LPS stimulation was 88.04 ± 2.13% (Figure 1G). Based on these results, this ratio was selected for subsequent experiments. The sizes of Fsi, FDsi, and ME@FDsi were 13.16 ± 2.069 nm, 57.75 ± 6.566 nm, and 127.7 ± 18.35 nm, respectively (Figure 1H). Their zeta potentials were ‐7.33 ± 0.8077 mV, ‐26.35 ± 1.12 mV, and ‐20.5 ± 1.218 mV, respectively (Figure 1I). To verify the stability of FDsi and ME@FDsi nanoparticles, both were dispersed in PBS and FBS, and their size changes were monitored over 72 h. The unmodified FDsi was relatively unstable in serum, whereas ME@FDsi maintained good size stability in both PBS and FBS over 72 h (Figure S1), benefiting from the hydrophilicity and stabilizing effect of glycans on the cell membrane surface [35]. Leveraging the established long‐circulation advantage of erythrocyte membranes [36, 37], we evaluated the release profile of this biomimetic delivery system in vitro. The results (Figure S2) showed sustained and slow drug release over 72 h, avoiding rapid drug degradation and loss. This sustained release behavior confirms that the erythrocyte membrane shell acts as an effective diffusion barrier and helps maintain the structural integrity of the carrier during circulation. Transmission electron microscopy (TEM) revealed that Fsi, FDsi, and ME@FDsi exhibited tetrahedral, spherical, and core–shell morphologies, with sizes of approximately 10–20 nm, 20–40 nm, and 120–130 nm, respectively (Figure 1J). To assess whether membrane proteins were preserved after coating, we performed SDS‐PAGE analysis. The protein profile of erythrocyte membrane‐coated nanoparticles closely resembled that of purified erythrocyte membranes (Figure S3), confirming minimal protein alteration during preparation. Furthermore, flow cytometry analysis showed that ME@FDsi modified with the MG1 peptide exhibited stronger targeting ability toward M1‐type microglia, indicating successful MG1 peptide modification (Figure 1K,L).

### 2.2 ME@FDsi's Targeted Activation of Microglia In Vitro and In Vivo

The blood‐brain barrier (BBB) is a complex structure comprising not only tight junctions between endothelial cells but also other cells forming the neurovascular unit, which collectively participate in the precise regulation of the BBB. Effective drug penetration across the BBB is crucial for treating SAE. We utilized a Transwell in vitro model to verify that ME@FDsi can penetrate brain microvascular endothelial cells. After constructing the in vitro BBB model, 1 µg/mL LPS was added for 24 h to simulate a compromised BBB. Subsequently, Cy3‐labeled Fsi, FDsi, and ME@FDsi were added to the upper chamber of the Transwell, and the fluorescence intensity in the lower chamber was measured using a fluorescence spectrometer at 2, 6, and 12 h. The results showed that the penetration rate of ME@FDsi across the BBB was significantly higher than that of Fsi and FDsi and increased in a time‐dependent manner (Figure S4A). In addition, to evaluate the transport efficiency of ME@FDsi under different BBB conditions, two additional control groups were established in the Transwell model: an intact BBB group (bEnd.3 monolayer treated with PBS) and a disrupted BBB group (bEnd.3 monolayer treated with 1 µg/mL LPS for 24 h). Cy3‐labeled ME@FDsi was added to the upper chamber, and the fluorescence intensity in the lower chamber was measured at 2, 6, and 12 h post‐addition (Figure S4B). The results showed that in the intact BBB group, the transmembrane transport rate of ME@FDsi remained relatively unchanged at all time points. Although a slight upward trend was observed within 12 h, this increase did not reach statistical significance, indicating that the tight junctions of the intact BBB effectively restricted passive diffusion of the nanoparticles. In striking contrast, under pathological conditions simulating SAE (LPS‐treated group), ME@FDsi exhibited a significant, time‐dependent, and statistically significant enhancement in permeability, with a penetration rate approximately 3.3‐fold higher than that of the control group at 12 h. These results confirm that the BBB penetration ability of ME@FDsi is strictly dependent on BBB integrity, enabling efficient brain entry only under SAE‐associated inflammatory conditions—establishing a foundation for its subsequent in vivo efficacy in targeting M1 microglia. The above results demonstrate that ME@FDsi BBB penetration is inflammation‐dependent. However, penetrating the BBB is only the first step—whether the drug can be effectively taken up by target cells (M1 microglia) is critical for therapeutic efficacy. Therefore, we established four combinations in the same Transwell system to comprehensively evaluate the inflammation‐dependent targeted uptake of ME@FDsi: intact BBB + resting BV2, intact BBB + M1 BV2, disrupted BBB + resting BV2, and disrupted BBB + M1 BV2. After 12 h of incubation, flow cytometry analysis (Figure S5) revealed that ME@FDsi uptake by BV2 cells exhibited significant dual inflammation dependence, with uptake in the disrupted BBB + M1 BV2 group being significantly higher than in all other groups. These results reveal that the brain‐targeted accumulation of ME@FDsi depends on two synergistic inflammatory signals: disruption of BBB integrity (allowing particle entry) and M1 activation of microglia (promoting particle uptake). This dual inflammation dependence ensures precise delivery of the drug to target cells under SAE pathological conditions while maintaining minimal accumulation in healthy brain tissue, providing an important basis for its translational potential.

**FIGURE 2:**
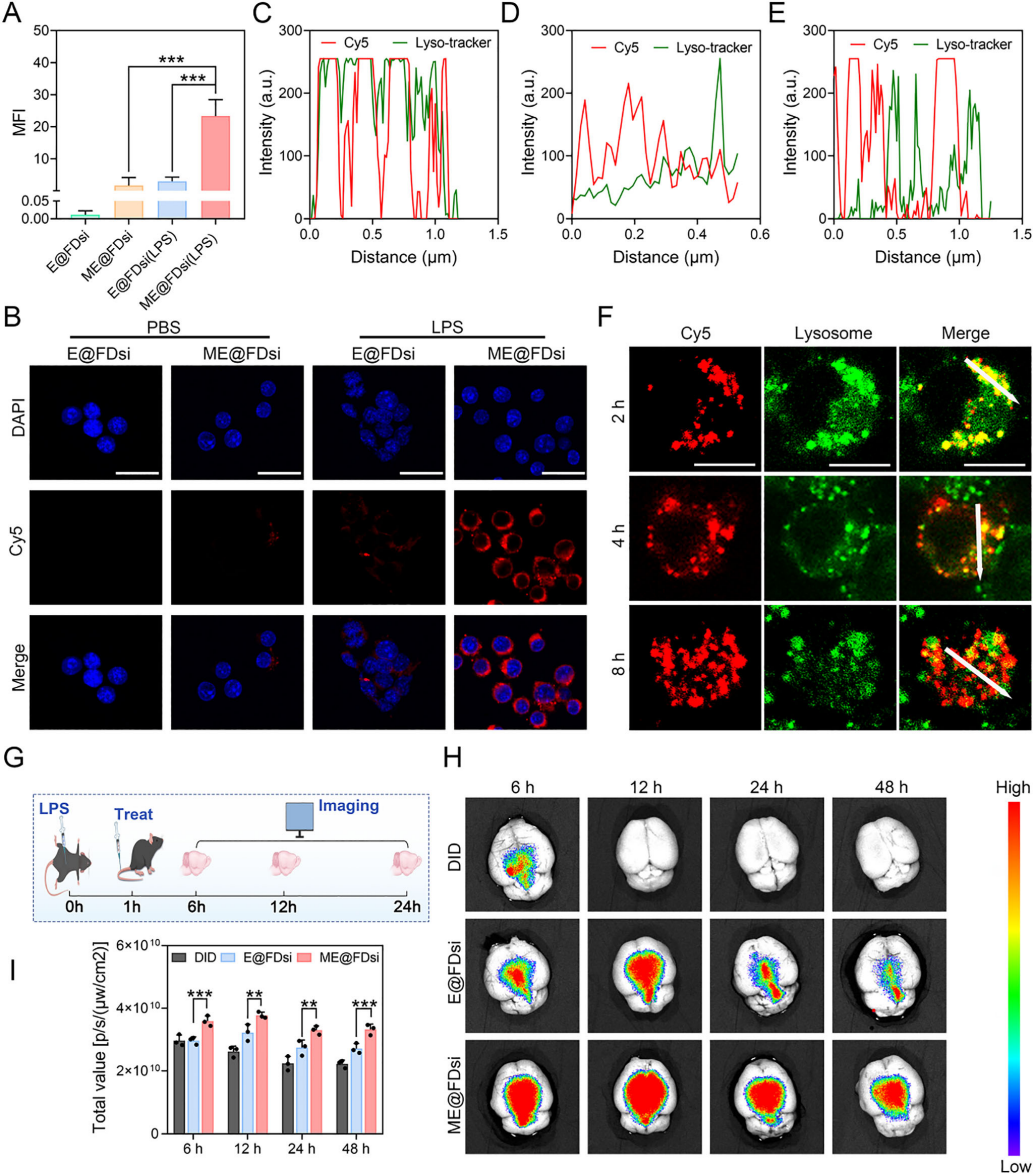
Blood‐brain barrier penetration capability and specific targeting of M1 microglia. (A, B) Cellular uptake of E‐FDsi and ME@FDsi after 4 h was characterized in both BV2 and BV2‐M1 (LPS‐treated) cells. E‐FDsi and ME@FDsi were labeled with the Cy5 dye (red). Scale bar: 30 µm. Quantitative analysis of the uptake levels of E‐FDsi and ME@FDsi across different treatment groups is shown. Data are presented as mean ± SD (*n* = 3). (C, D, E, F) To detect the lysosomal escape ability of the nanoparticles, Cy5‐labeled ME@FDsi was prepared. BV2 cells were seeded in dishes and cultured for 24 h. The cells were then stimulated with 1 µg/mL LPS to simulate an inflammatory response. ME@FDsi was added to the LPS‐induced cells and incubated for 2, 4, or 8 h, followed by staining of lysosomes with Lysotracker Green. Scale bar: 10 µm. Fluorescence intensity analysis was performed on the regions of interest (white arrows) in the images. (G) Schematic diagram of the administration for small animal imaging. Mice were intraperitoneally injected with 10 mg/kg LPS, followed by injection of Cy5‐labeled E‐FDsi and ME@FDsi after 1 h. Brain tissues were collected for imaging at 6, 12, and 24 h. (H, I) Imaging results of mouse brain tissues collected at 6, 12, 24, and 48 h. Data are presented as mean ± SD (*n* = 3). Statistical significance was determined by one‐way ANOVA and two‐way ANOVA. ^*^p < 0.05, ^**^p < 0.01, ^***^p < 0.001; *n.s*. indicates no significant difference.

The MG1 peptide is a homing peptide that specifically recognizes M1 microglia, identified and isolated via phage display technology [30]. To further investigate whether this targeting effect stems from the precise selectivity of the MG1 peptide for the M1 phenotype rather than non‐specific cell binding, we conducted a series of in vitro and in vivo experiments. Microglia were activated using lipopolysaccharide (LPS)—one of the most potent stimuli for microglial activation due to its interaction with Toll‐like receptor 4 (TLR4) on the microglial surface. BV2 cells were treated with 1 µg/mL LPS for 24 h [38], with a PBS‐treated group (resting microglia) serving as the control. The targeting effects of E@FDsi and ME@FDsi in BV2 cells were assessed via immunofluorescence. The results demonstrated that the uptake efficiency of ME@FDsi by M1 microglia was significantly higher than that of E@FDsi, whereas resting microglia showed no significant difference in uptake between ME@FDsi and E@FDsi, confirming the specific recognition capability of M1 microglia for MG1 peptide‐modified vesicles (Figure 2A,B). Notably, the mean fluorescence intensity of ME@FDsi in BV2 cells was 8 times higher than that of E@FDsi, indicating efficient entry of these vesicles into pro‐inflammatory M1 phenotype microglia. To verify whether the targeting effect is specific to M1 microglia, we first stimulated BV2, Raw264.7, and HUVEC cells with PBS, LPS, and IL‐4, respectively (Figure S6A–D). The results showed that in addition to exhibiting significant targeting toward M1 microglia, ME@FDsi also displayed a certain degree of targeting toward Raw264.7 cells. However, no obvious targeting was observed toward resting or M2‐type macrophage cell lines or HUVEC cells, preliminarily confirming the highly selective targeting capability of the MG1 peptide‐functionalized nanosystem for the M1 phenotype. To further simulate the complex microenvironment with multiple cell types coexisting in vivo and to exclude the possibility of non‐specific uptake caused by general inflammatory activation, we established a co‐culture system of LPS‐stimulated BV2, Raw264.7, and HT22 cells to more rigorously evaluate the cell type selectivity of ME@FDsi. The three co‐cultured cell types were pretreated with 1 µg/mL LPS for 24 h to simulate an inflammatory state, followed by incubation with Cy3‐labeled ME@FDsi for 4 h. Uptake of ME@FDsi by each cell population was detected by flow cytometry. As shown in Figure S7A,B, ME@FDsi exhibited the highest uptake efficiency in LPS‐stimulated BV2 cells. Raw264.7 cells also showed a certain degree of uptake, but this was significantly lower than that of BV2 cells, while uptake by HT22 cells was extremely low. These results further confirm in a co‐culture system that more closely mimics in vivo complexity that the targeting effect of ME@FDsi exhibits cell type selectivity: preferential recognition and uptake of M1‐like microglia, with minimal non‐specific uptake by non‐immune cells such as neurons.

Furthermore, the ability of nanodrugs to escape from lysosomes is crucial for their function. We used a green fluorescent lysosomal probe to label lysosomes and tracked the localization of ME@FDsi after cellular entry via fluorescence microscopy. Experimental results showed that yellow fluorescence signals appeared in cells after 2 h of co‐incubation with ME@FDsi (Figure 2C–F). This signal resulted from the overlap of red fluorescence from Cy5‐labeled nanomaterials and green fluorescence from lysosomes, indicating successful internalization and localization of ME@FDsi nanocomposites within lysosomes after 2 h. After 4 h of co‐incubation, the yellow signal resulting from the overlap of red and green fluorescence began to diminish, with partial separation of red and green fluorescence observed in some regions, suggesting that the earliest detectable lysosomal escape events may occur at 4 h. By 8 h of co‐incubation, the overlapping yellow signal further decreased, and red and green fluorescence became clearly separated, indicating that the lysosomal escape efficiency of ME@FDsi reached a higher level at 8 h. However, lysosomal escape alone does not equate to successful functional delivery—whether the cargo retains its bioactivity during the escape process is critical for therapeutic efficacy. Therefore, we further evaluated whether the loaded DSF and Fsi remained functionally active after incubating ME@FDsi in a simulated lysosomal environment (pH 5.0) for 8 h. As shown in Figure S8A, the functional integrity of DSF was assessed by measuring relative ALDH enzyme activity. The Control group was set as baseline activity (100%). The pure DSF group (not incubated in a lysosomal environment) significantly inhibited ALDH enzyme activity, with an inhibition rate of 66.5%. The ME@Fsi group (without DSF loading) showed no inhibition of ALDH, excluding interference from tFNA/siTNFα. In contrast, ME@FDsi incubated at pH 5.0 for 8 h still significantly inhibited ALDH activity, with an inhibition rate of 46.85%. Although slightly lower than that of the pure DSF group (likely due to partial degradation in the lysosomal environment), these results demonstrate that DSF retained its functional inhibitory capacity after escape. Meanwhile, we assessed the integrity of Fsi in ME@FDsi after incubation in pH 5.0 buffer for 1, 2, 4, and 8 h using agarose gel electrophoresis. As shown in Figure S8B, the Fsi bands were clear and intact at 1 and 2 h, indicating structural stability during the initial phase of lysosomal retention. At 4 h, the bands began to weaken, coinciding with the onset of lysosomal escape. At 8 h, distinct Fsi bands remained detectable, confirming that at least a portion of the cargo successfully escaped while maintaining structural integrity. Together, these results demonstrate that the lysosomal escape of ME@FDsi is functional—both DSF and Fsi retained bioactivity after escape, providing the structural foundation for their subsequent synergistic anti‐inflammatory and anti‐pyroptotic effects.

Given the limitations of in vitro blood‐brain barrier (BBB) models in fully recapitulating the complexity and real‐time functionality of the BBB in vivo, we further evaluated the in vivo targeting efficacy by intravenously injecting fluorescently labeled nanoparticles into SAE mice via the tail vein. To determine whether the accumulation of ME@FDsi in the brain is inflammation‐dependent, we established three groups: a healthy control group (no LPS), a moderate inflammation group (10 mg/kg LPS), and a severe inflammation group (15 mg/kg LPS). Brain tissues were collected at 6, 12, and 24 h post‐injection for ex vivo fluorescence imaging. As shown in Figure S9, at 6 h post‐injection, the fluorescence intensity in the brain injury region of both LPS‐treated groups was significantly higher than that of the healthy control group. However, no significant difference was observed between the 10 and 15 mg/kg LPS groups, suggesting that BBB opening may have reached a plateau at this early stage, with initial particle entry primarily governed by BBB permeability. At 12 and 24 h, the fluorescence intensity in the 15 mg/kg LPS group was significantly higher than that in the 10 mg/kg LPS group, indicating that enhanced microglial activation accompanying aggravated inflammation promoted targeted accumulation. These results confirm that the brain accumulation of ME@FDsi is positively correlated with the severity of neuroinflammation. On this basis, we further validated the targeting effect of the MG1 peptide. As shown in Figure 2H,I, at 6, 12, and 24 h post‐injection, the fluorescence intensity in the brain injury region of the ME@FDsi group was significantly higher than that of the E@FDsi group, consistent with previous observations and confirming the targeting effect of the MG1 peptide. Notably, by 48 h, the fluorescence intensity of the free DiD group had decreased to background levels, indicating that the free dye was rapidly cleared or quenched in the brain. In contrast, both the E@FDsi and ME@FDsi groups still exhibited detectable fluorescence signals at 48 h, demonstrating that the signal attenuation reflects normal clearance and metabolism of the nanoparticles in the brain, rather than rapid quenching of the DiD dye.

Given the limitations of in vivo imaging in distinguishing specific cell populations, we further clarified the precise cellular localization of ME@FDsi in the brain using immunofluorescence colocalization and flow cytometry. As shown in Figure S10, DiD‐labeled ME@FDsi (red) exhibited strong colocalization with Iba1^+^ microglia (green) in the hippocampus and cortex, whereas minimal colocalization was observed with NeuN^+^ neurons or GFAP^+^ astrocytes. Flow cytometric analysis of brain myeloid populations (Figure S11, with the gating strategy shown in Figure S12) further revealed that ME@FDsi was primarily taken up by resident microglia (CD11b^+^ CD45^(low)), with a positivity rate of 59.08 ± 12.80%, significantly higher than that of infiltrating macrophages (CD11b^+^ CD45^(high), 33.72 ± 12.74%). These results demonstrate that ME@FDsi specifically targets M1 microglia in the brain parenchyma, with negligible non‐specific uptake by neurons, astrocytes, or infiltrating myeloid cells.

As the MG1 peptide recognizes the M1‐like inflammatory phenotype, which may also be present in activated peripheral macrophages, we further evaluated its potential binding to peripheral myeloid cells after confirming the brain‐targeting specificity of ME@FDsi. As shown in Figure S13 (gating strategy detailed in Figure S14), flow cytometric analysis of spleen cells from SAE mice revealed that the positivity rate of ME@FDsi in peripheral macrophages (CD11b^+^ F4/80^+^) was 23.42 ± 7.435%, significantly lower than that in brain resident microglia (59.08 ± 12.80%). These results indicate that although the MG1 peptide exhibits some degree of binding to peripheral myeloid cells under inflammatory conditions—likely due to their M1‐like polarization—its targeting efficiency for brain resident microglia remains significantly higher, ensuring preferential accumulation of the drug in the intended target cells.

In addition, to systematically evaluate the systemic distribution and off‐target accumulation of ME@FDsi, we collected the heart, liver, spleen, lungs, and kidneys from SAE mice at 6, 12, 24, 48, 72, 96, and 120 h after tail vein injection for ex vivo fluorescence imaging. As shown in Figure S15, ME@FDsi was predominantly distributed in the liver and spleen, with the strongest fluorescence signals observed at 6–24 h, followed by a gradual decrease over time and approaching background levels by 120 h, consistent with the typical clearance pattern of nanoparticles via the mononuclear phagocyte system. Fluorescence signals in the heart, kidneys and lungs remained at low levels throughout all time points. These results indicate that although ME@FDsi primarily accumulates in clearance organs such as the liver and spleen, it can be gradually eliminated within 120 h without significant long‐term off‐target retention risk.

### 2.3 ME@FDsi In Vitro Therapeutic Efficacy

**FIGURE 3:**
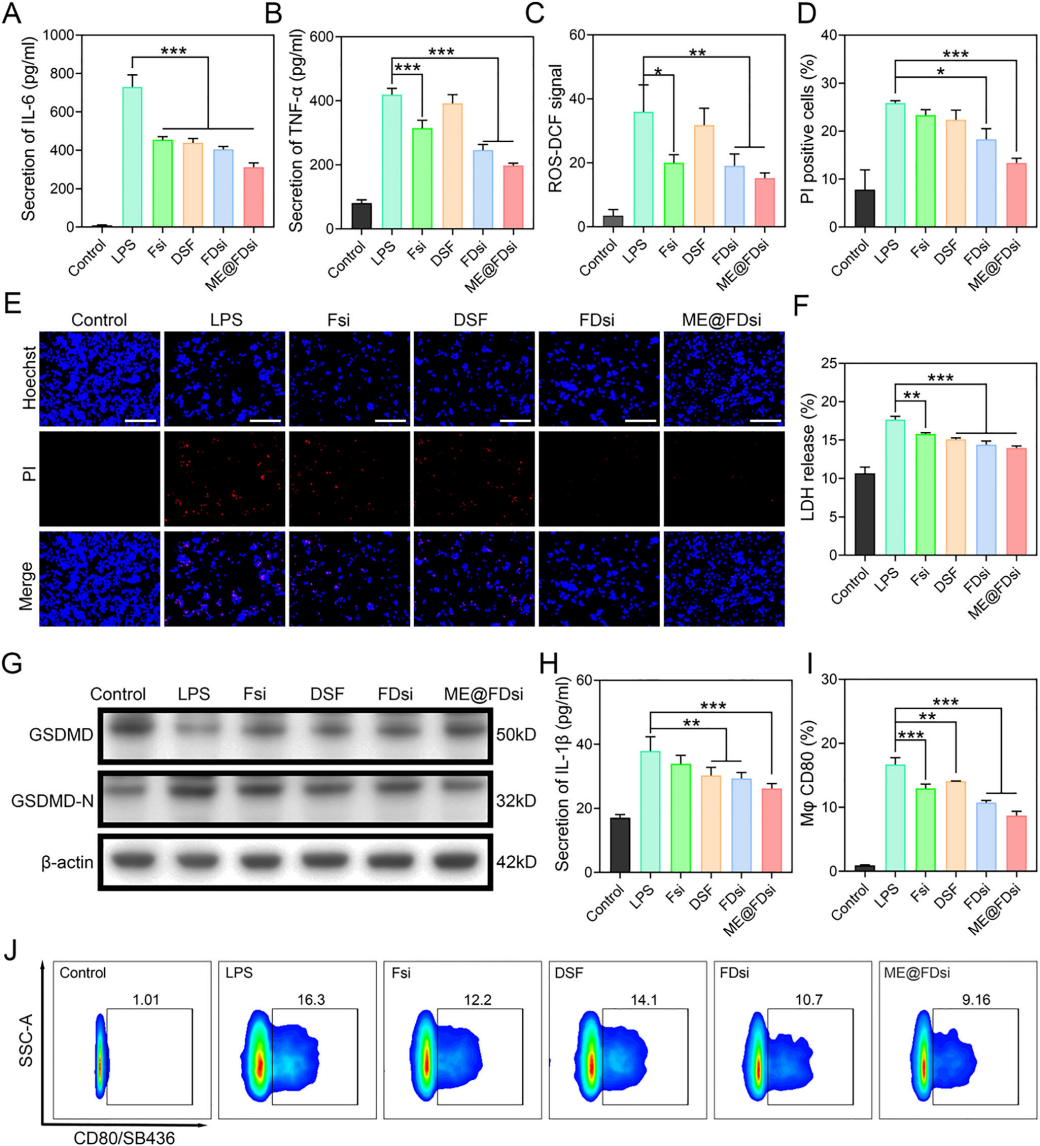
In vitro anti‐inflammatory effect evaluation of ME@FDsi. (A, B) Secretion of inflammatory cytokines, including IL‐6 and TNF‐α, from BV2 cells after LPS stimulation was detected by ELISA. (C) Intracellular reactive oxygen species (ROS) generation in BV2 cells under different treatment conditions was detected by flow cytometry, with showing the quantitative analysis of DCF fluorescence intensity. (D, E) BV2 cell status after different treatments was detected using Hoechst 33342 / Propidium Iodide (PI) double staining. Scale bar is 200 µm. (F) Lactate dehydrogenase (LDH) was released from BV2 cells after treatments with the indicated methods. (G) Western blot analysis showing the expression of pyroptosis‐related proteins GSDMD and GSDMD‐N in BV2 cells after various treatments. β‐Actin was used as a protein loading control. (H) Secretion of the inflammatory cytokine IL‐1β from BV2 cells after LPS stimulation was detected by ELISA. (I, J) Expression of the M1‐type cell marker CD80 in BV2 cells under different treatment conditions was detected by flow cytometry, with (I) showing the quantitative analysis of CD80 levels. Statistical significance was determined by one‐way ANOVA. ^*^p < 0.05, ^**^p < 0.01,^***^p < 0.001; *n.s*. indicates no significant difference.

Microglia, the brain's resident macrophages, play a key role in regulating inflammatory balance through their unique polarization states—M1 (pro‐inflammatory) and M2 (anti‐inflammatory). To evaluate the anti‐inflammatory effects of our nanoformulations, we polarized BV2 microglial cells toward the M1 phenotype using LPS. After co‐incubating LPS‐activated microglia with free Fsi, free DSF, FDsi, or ME@FDsi, we measured IL‐6 and TNF‐α levels by ELISA (Figure 3A,B). The LPS‐treated group showed significant upregulation of pro‐inflammatory cytokines (such as IL‐6 and TNF‐α), indicating BV2 cell activation toward the M1 pro‐inflammatory state. Treatment with all nanoformulations significantly inhibited IL‐6 and TNF‐α expression. Notably, both Fsi and DSF effectively suppressed IL‐6, indicating their anti‐inflammatory potential. However, only Fsi—not DSF—significantly reduced TNF‐α, highlighting the specific gene‐silencing effect of siTNF‐α. We then used the DCFH‐DA probe to assess the reactive oxygen species (ROS) scavenging effect of the nanodrugs on BV2 cells via flow cytometry and inverted fluorescence microscopy. Flow cytometry results (Figure S16; Figure 3C) showed that compared to cells treated with LPS alone, DSF had relatively weak antioxidant capacity, while the nanodrugs significantly reduced LPS‐induced ROS generation. Fluorescence microscopy results in Figure S17 also confirmed this conclusion. Furthermore, the supernatant of cells treated with LPS and nigericin showed increased lactate dehydrogenase (LDH) release and a rise in PI‐positive cell count (Figure 3D–F), indicating cell death due to loss of plasma membrane integrity. After treatment with free drugs and nanoparticles, both LDH release and the number of PI‐positive cells decreased. To further verify whether LPS‐induced cell death is mediated by GSDMD‐dependent pyroptosis, we detected GSDMD levels by Western blot (Figure S18; Figure 3G), finding increased GSDMD cleavage and significantly elevated expression of GSDMD‐N, which decreased after treatment. Since pyroptosis involves GSDMD‐N forming pores in the cell membrane, allowing release of activated inflammatory factors (such as IL‐1β) from the cell and triggering a strong inflammatory response, we used an ELISA kit to validate IL‐1β protein expression (Figure 3H). The results showed that treatment with DSF, FDsi, and ME@FDsi inhibited IL‐1β expression, with ME@FDsi exhibiting a superior inhibitory effect. Inflammatory responses have the capacity to recruit or activate more pro‐inflammatory M1 microglia. Therefore, we further investigated the effect of nanoparticles on BV2 cell phenotype polarization. After LPS treatment (Figure 3I,J), CD80 expression significantly increased in BV2 cells, indicating a shift in polarization toward the pro‐inflammatory M1 phenotype. Treatment with Fsi, FDsi, and ME@FDsi nanoparticles all showed inhibitory effects on CD80 expression, demonstrating that our materials can suppress the transition of BV2 cells to the M1 phenotype.

To further confirm the necessity of the engineered nanostructure in a complex cellular environment, we introduced a key control group—the simple mixture group (Mix), which contained equivalent amounts of free DSF, tFNA, siTNFα, and erythrocyte membrane fragments. Under the same experimental conditions as ME@FDsi, we compared the anti‐inflammatory, antioxidant, and anti‐pyroptotic effects of each group on LPS‐stimulated BV2 cells. As shown in Figures S19–S22, compared with the LPS group, the Mix group exhibited a certain degree of inhibition on various inflammatory indicators, suggesting that the individual components in a simply mixed state can exert some functions at the cellular level. However, the inhibitory effects of the ME@FDsi group were significantly superior to those of the Mix group: further reduced secretion levels of IL‐6 and TNF‐α (Figure S19), significantly decreased ROS generation (Figure S20), lower proportion of PI‐positive cells (Figure S21A,B), enhanced inhibition of IL‐1β release (Figure S21C), and a significantly lower proportion of CD80‐positive cells compared to the Mix group (Figure S22). Notably, in the detection of TNF‐α, the Mix group showed no significant difference compared with the LPS group (Figure S19B), while the ME@FDsi group significantly inhibited TNF‐α secretion. This difference is highly consistent with the phenomenon observed in Figure 1E: tFNA effectively protects siTNF‐α from nuclease degradation in serum (Figure 1E), whereas free siTNF‐α is completely degraded within 2 h. In the Mix group, siTNFα lacked the protection of tFNA and was rapidly degraded during incubation, failing to exert its gene silencing function. In the ME@FDsi group, tFNA stably carried siTNFα through complementary base pairing, protected its integrity, and enabled successful delivery to the cytoplasm to exert RNA interference effects. These results further confirm that the tFNA carrier is indispensable for the functional efficacy of siTNFα. Collectively, these results provide strong evidence that the observed therapeutic effects do not arise from simple additive effects of individual components, but rather from the precise targeting, protection from enzymatic degradation, and efficient intracellular delivery conferred by the engineered nanostructure, fully demonstrating the structural advantages of assembled nanovesicles in achieving synergistic intracellular release. Considering that previous experiments found the MG1 peptide also has some targeting effect on Raw 264.7 cells, we simultaneously used LPS to stimulate Raw 264.7 cells to simulate an in vitro inflammatory environment and used ELISA kits to detect the inflammatory factors IL‐6, TNF‐α, and IL‐1β (Figure S23A–C). When treated with the nanodrugs, the expression of IL‐6, TNF‐α, and IL‐1β was significantly inhibited. Subsequently, the DCFH‐DA probe was used to assess the ROS scavenging effect of the nanodrugs on Raw 264.7 cells via flow cytometry. Flow cytometry analysis (Figure S24A,B) revealed a marked suppression of LPS‐evoked ROS production by the nanodrugs relative to LPS treatment alone. The same results were observed under inverted fluorescence microscopy (Figure S25).

In summary, LPS mimics bacterial infection to trigger an inflammatory response, further stimulating oxidative stress and elevated levels of pro‐inflammatory cytokines. This process may induce pyroptosis, leading to lytic cell death and the release of more inflammatory factors, thereby activating and recruiting M1 microglia. Internalization of these nanoparticles is followed by the delivery of encapsulated drugs into the cytoplasmic compartment, leading to the desired pharmacological effects. Drug release alleviates oxidative stress by scavenging ROS. Furthermore, this therapy inhibits the expression of pro‐inflammatory cytokines and cellular pyroptosis, reduces microglial polarization toward the M1 phenotype, thereby mitigating the inflammatory response.

### 2.4 ME@FDsi Reduces Brain Inflammation and Improves the Brain's Microenvironment

**FIGURE 4:**
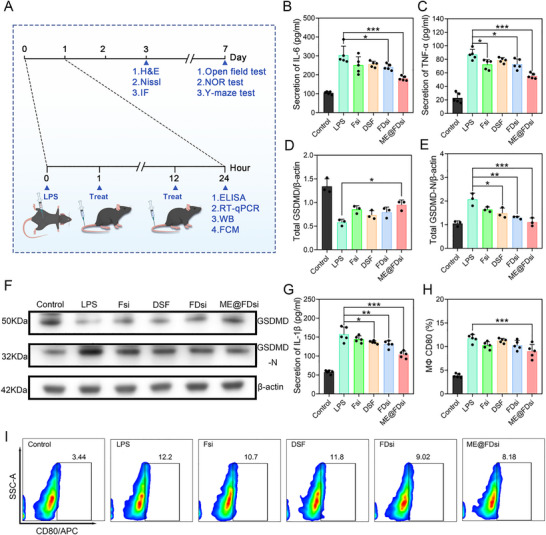
Evaluation of the anti‐inflammatory effects of ME@FDsi in the brains of septic mice. (B, C) Secretion of inflammatory cytokines, including IL‐6 and TNF‐α, in the brain tissue of septic mice was detected by ELISA. (D, E) Quantitative results of the expression levels of full‐length GSDMD and cleaved GSDMD‐N relative to β‐actin. (F) Western blot analysis showing the expression of pyroptosis‐related proteins GSDMD and GSDMD‐N in the brain tissue after various treatments. β‐Actin was used as a protein loading control. (G) Secretion of the inflammatory cytokine IL‐1β in the brain tissue of septic mice was detected by ELISA. (H, I) Flow cytometric analysis of the CD80+ immune cell population in mouse brain tissue and quantitative analysis of CD80 levels. Statistical significance was determined by one‐way ANOVA. ^*^p < 0.05, ^**^p < 0.01, ^***^p < 0.001; *n.s*. indicates no significant difference.

Given that cognitive dysfunction in SAE is primarily driven by excessive inflammatory factors (e.g., TNF‐α and IL‐1β) that cross the compromised blood‐brain barrier or directly act on brain tissue, we assessed their levels in brain homogenates by ELISA (Figure 4B,C). The results indicated that the expression of both IL‐6 and TNF‐α was inhibited, with ME@FDsi showing the best effect. It is noteworthy that although free drugs also exhibited some inhibitory effect on the pro‐inflammatory cytokine IL‐6 in prior cell experiments, they showed no statistical significance in brain treatment. This is likely due to the short half‐life of free drugs in the complex in vivo environment and their insufficient concentration reaching the brain, highlighting the advantages of nanodrugs. In contrast, the Fsi nanoparticles loaded with siTNFα not only precisely targeted TNF‐α in vitro but also effectively suppressed TNF‐α levels in the brain model, fully validating the efficacy of their targeted therapy and the critical value of the nanocarrier for brain drug delivery. Meanwhile, we further verified the expression of inflammatory factors using qPCR (Figure S26) and obtained consistent results. Furthermore, during sepsis, various inflammatory factors activate microglia and induce pyroptosis. During pyroptosis, cells release large quantities of pro‐inflammatory factors, which further recruit more immune cells to gather in the brain, forming an inflammatory storm and disrupting the neural tissue microenvironment. GSDMD is the key executioner molecule mediating pyroptosis, whose core function is to form pores in the cell membrane upon activation, leading to cell swelling and rupture, and the release of pro‐inflammatory substances [39]. To clarify whether pyroptosis was activated, we first detected the expression of GSDMD and its activated fragment (GSDMD‐N) in brain tissue by Western Blot. As shown in Figure 4D–F, the level of GSDMD‐N was significantly upregulated in the LPS model group, indicating that GSDMD was extensively cleaved and activated, initiating the pyroptosis process. After treatment, the generation of GSDMD‐N was significantly inhibited, demonstrating that the key execution step of pyroptosis was blocked. Subsequently, to confirm the downstream inflammatory effects of pyroptosis, we examined the release and expression of IL‐1β by ELISA and qPCR (Figure 4G; Figure S27). The results showed that both the release of mature IL‐1β and its mRNA levels were significantly reduced, providing functional evidence that our therapeutic strategy effectively inhibited pyroptosis, thereby reducing the consequent release of inflammatory factors. As M1 microglia are pro‐inflammatory immune cells in the central nervous system that initiate inflammatory responses under pathological conditions, exacerbating neural damage and impeding the improvement of SAE, we analyzed the polarization state of microglia using flow cytometry (Figure 4H,I; Figure S28 shows the gating strategy). We found that LPS significantly increased CD80^+^ microglia, indicating a shift toward the M1 phenotype. Free drugs caused a slight, non‐significant reduction, whereas ME@FDsi markedly decreased CD80^+^ cells, demonstrating effective inhibition of M1 polarization. These results demonstrate that systemic inflammation induced by LPS triggers pyroptosis, leading to GSDMD activation and the release of inflammatory factors such as IL‐1β. These factors further recruit and promote microglial polarization toward the pro‐inflammatory M1 phenotype. The activated M1 microglia, in turn, release new inflammatory mediators, exacerbating pyroptosis and forming a self‐amplifying “vicious inflammatory cycle” that ultimately drives neural damage and the progression of SAE. Our nanotherapy, on one hand, directly reduces the secretion of pro‐inflammatory factors, and on the other hand, by inhibiting GSDMD‐mediated pyroptosis, not only directly reduces IL‐1β release but, more critically, blocks the core driver of this vicious cycle, thereby reducing M1 microglial activation and providing an effective therapeutic strategy for alleviating neuroinflammation and tissue damage.

To further verify whether the nanostructure of ME@FDsi is essential for its anti‐inflammatory effects in the brain, we similarly established a key control group in which DSF, tFNA, siTNFα, and erythrocyte membrane vesicles were simply mixed (Mix group) without assembly into a complete nanostructure. The therapeutic effects of the Mix group and the ME@FDsi group on brain inflammation were compared in the SAE mouse model. As shown in Figure S29, compared with the LPS group, the Mix group exhibited extremely limited inhibitory effects on brain inflammatory indicators: the protein levels of IL‐6 and TNF‐α, the release of IL‐1β, and the proportion of CD80‐positive microglia (Figure S30, with the gating strategy shown in Figure S31) showed no significant differences from the LPS group. In contrast, the ME@FDsi group significantly inhibited all the aforementioned indicators. These results further clearly demonstrate that the anti‐inflammatory effect of ME@FDsi in the brain depends on its intact nanovesicle structure—simply mixed components cannot be effectively delivered to the target brain regions nor exert synergistic anti‐inflammatory and anti‐pyroptotic effects.

### 2.5 ME@FDsi Improves Cognitive Dysfunction in SAE Mice

**FIGURE 5:**
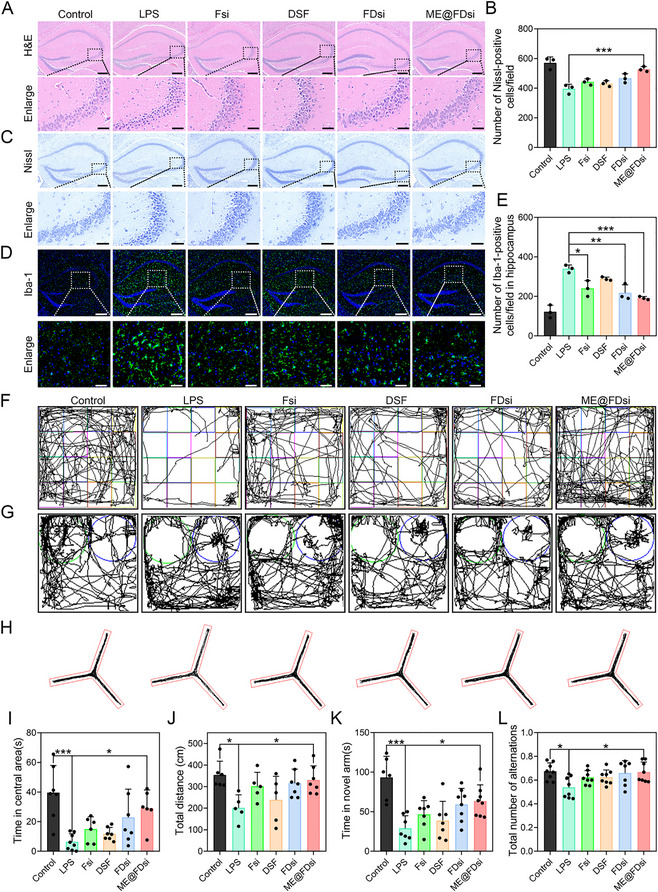
ME@FDsi ameliorates cognitive dysfunction in SAE mice. (A) Representative images of H&E staining of brain tissue on day 3 post‐modeling (scale bar: 200 µm) and magnified views (scale bar: 50 µm). (B) Quantitative analysis of Nissl‐positive cells. (C) Representative images of Nissl‐positive cells on day 3 post‐modeling (scale bar: 200 µm) and magnified views (scale bar: 50 µm). (D) Representative immunofluorescence images showing Iba‐1 expression in the injured hippocampal region of septic model mice from different treatment groups on day 3 post‐treatment (scale bar: 200 µm) and magnified views (scale bar: 50 µm). (E) Quantitative analysis of Iba‐1‐positive cells in the injured hippocampal region of septic model mice from different treatment groups on day 3 post‐treatment. (E) Quantitative analysis of Iba‐1‐positive cells in the injured hippocampal region of septic model mice from different treatment groups on day 3 post‐treatment. (F) Movement trajectories of septic model mice from different treatment groups in the open field test performed on day 7 post‐treatment. (G) Movement trajectories of septic model mice from different treatment groups in the novel object recognition test performed on day 7 post‐treatment. (H) Movement trajectories of septic model mice from different treatment groups in the Y‐maze test performed on day 7 post‐treatment. (I, J) (I) Statistical graph of the time spent in the central zone by mice from different groups. (J) Statistical graph of the total distance traveled by mice from different groups. (K) Statistical graph of the time spent exploring the novel object by mice from different groups. (L) Statistical graph of the correct alternation rate of mice from different groups. Statistical significance was determined by one‐way ANOVA. ^*^p < 0.05, ^**^p < 0.01, ^***^p < 0.001; *n.s*. indicates no significant difference.

To further validate the neuroprotective effects of ME@FDsi, we assessed neuronal necrosis and brain tissue damage in mice three days after LPS modeling. Hematoxylin and eosin (H&E) staining revealed that brain tissue damage in the ME@FDsi group was significantly alleviated compared to other treatment groups (Figure 5A). Nissl staining revealed a marked reduction in neuronal survival in SAE mice (395.3 ± 31.18) compared to the PBS group (571.0 ± 39.95). ME@FDsi treatment significantly restored neuronal survival (528.0 ± 17.35, Figure 5B,C). To evaluate the level of neuroinflammation in brain tissue post‐modeling, we detected the expression of ionized calcium‐binding adapter molecule 1 (Iba‐1)—a marker for microglial activation. Following brain tissue injury, microglia rapidly activate, exhibiting swollen and hypertrophied morphological characteristics. As resident immune cells in the central nervous system, microglia possess sensing, protective, and defensive functions. In neurodegenerative diseases, microglial dysfunction can exacerbate neuronal damage. Therefore, we assessed the expression levels via immunofluorescence to evaluate the degree of neuroinflammation in brain tissue during SAE (Figure 5D,E). The hippocampus, critical for learning and memory, is particularly vulnerable in SAE, and its damage correlates with patient prognosis. The study found that in the ME@FDsi treatment group, the number of Iba‐1‐positive cells in the hippocampus of the brain injury area was significantly reduced on day 3 post‐modeling. These results indicate that ME@FDsi treats SAE by mitigating neuronal damage and reducing microglial activation. Given that the cortex also contributes to recognition memory and executive function, we extended our analysis to this region. As shown in Figures S32–S34, ME@FDsi similarly attenuated cortical neuronal damage (H&E and Nissl staining) and microglial activation (Iba‐1 immunofluorescence), with results mirroring those in the hippocampus. These parallel neuroprotective effects in both brain regions provide a comprehensive histological basis for the improved cognitive function observed in ME@FDsi‐treated mice during behavioral tests.

We assessed the learning ability and spatial memory function of mice after SAE using the open field test, novel object recognition test, and Y‐maze test. In the open field test, SAE mice tended to stay in the peripheral areas and showed reduced desire to explore the central space (Figure 5F,I,J). The time spent exploring the center zone was only 6.324 ± 5.560 s in the LPS group. In contrast, ME@FDsi‐treated mice showed a significant increase to 29.27 ± 12.15 s, which was 4.62 times that of the LPS group (6.324 ± 5.560 s). This performance reached 74% of the level observed in healthy controls (39.56 ± 18.36 s). Although other treatment groups showed some degree of improvement, the differences were not statistically significant. These results demonstrated that mice treated with ME@FDsi exhibited significantly better recovery in learning and spatial memory capabilities compared to other treatment groups. In the novel object recognition test (Figure 5G,K), ME@FDsi‐treated mice spent 63.46 ± 20.55 s of their time exploring the novel object, representing a significant improvement of 34.55% compared to the LPS group (28.91 ± 15.98 s). This performance reached 68% of the level observed in healthy controls (93.33 ± 26.68 s), indicating that ME@FDsi not only alleviated SAE‐associated cognitive deficits but also achieved near‐normal cognitive function recovery. Consistent results were obtained in the Y‐maze test (Figure 5H,L), where the ME@FDsi treatment group showed a higher correct alternation rate (0.6676 ± 0.08482) than the LPS group (0.5384 ± 0.08909), further supporting that ME@FDsi can improve cognitive function in SAE mice. The consistency of therapeutic benefits across three independent behavioral paradigms—each assessing distinct cognitive domains—provides robust evidence that ME@FDsi achieves genuine disease modification rather than isolated symptomatic relief. Notably, the near‐complete restoration of cognitive function (reaching 68%–74% of control levels) is particularly striking given the severity of SAE‐induced cognitive deficits and the current lack of effective treatments for this condition. These functional improvements align closely with our molecular and histological findings of reduced neuroinflammation and neuronal protection, collectively demonstrating that the multi‐target mechanism of ME@FDsi translates into clinically meaningful cognitive recovery and offers translational potential for SAE therapy. It is noteworthy that the free drug group and the FDsi group, although showing a trend toward cognitive improvement, did not reach statistical significance compared to the LPS group. This might be because they influenced the brain microenvironment to some extent through anti‐inflammatory and anti‐pyroptotic effects, but their inability to precisely target the brain inflammation greatly reduced their efficacy. ME@FDsi addressed this issue by targeting microglia in the brain, further demonstrating the advantage of our drug in treating SAE.

In the aforementioned experiments, we confirmed that the integrity of the nanostructure is crucial for the anti‐inflammatory effects of ME@FDsi at both the cellular level (Figures S19–S22) and the brain tissue inflammation level (Figures S29–S31). To further investigate whether this structural dependence ultimately affects neurological function, we compared the effects of intact ME@FDsi and a simple mixture of its components (Mix group) on neuropathology and behavior in the SAE mouse model. As shown in Figures S35–S38, in the SAE mouse model, the simple mixture (Mix group) failed to ameliorate LPS‐induced neuronal damage: H&E staining of the hippocampus and cortex in the Mix group still revealed marked neuronal damage and tissue vacuolation (Figure S35), the number of Nissl staining‐positive neurons showed no significant difference from the LPS group (Figure S36), and the degree of IBA1‐positive microglial activation was also comparable to that of the LPS group (Figure S37). Concurrently, to evaluate the durability of the therapeutic effect of ME@FDsi, we extended the behavioral observation to day 14 post‐treatment. Consistent with the neuropathological results, the Mix group also failed to improve cognitive function in behavioral tests. As shown in Figure S38, at both day 7 and day 14 time points, the performance of mice in the Mix group in the open field test and novel object recognition test showed no significant difference from the LPS group, whereas the ME@FDsi group exhibited significant and sustained cognitive improvement. Notably, the novel object exploration time in the ME@FDsi group on day 14 was significantly higher than on day 7. This may be attributed to the sustained drug release property of ME@FDsi (Figure S2), allowing the therapeutic effect to accumulate over time; concurrently, nerve repair and synaptic plasticity remodeling are inherently time‐dependent processes, and with the progressive improvement of the brain's immune microenvironment, cognitive function reached a more optimal level by day 14. There was no significant difference in the open field test results within the ME@FDsi group between day 7 and day 14, indicating that the improvement in anxiety‐like behavior remained stable over the two‐week period. These results collectively confirm that the neuroprotective effect of ME@FDsi strictly depends on its intact nanovesicle structure—only by precisely assembling the components into a nanostructure can effective brain delivery and synergistic treatment be achieved, ultimately translating into significant and durable cognitive improvement.

### 2.6 ME@FDsi In Vivo Therapeutic Efficacy

**FIGURE 6:**
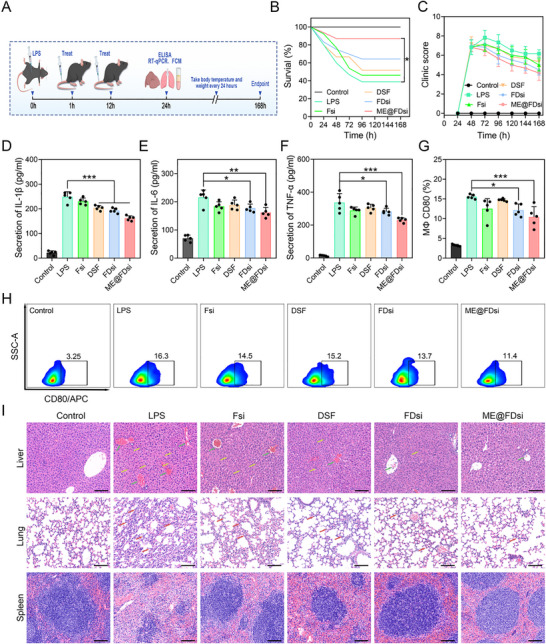
Evaluation of the therapeutic effect of ME@FDsi on septic mice. (A) Schematic diagram of the treatment timeline for LPS‐induced septic mice receiving various treatments. (B) Survival rate of septic mice (*n* = 15). (C) Clinical scores of SAE mice in different treatment groups. (D–F) Secretion of inflammatory cytokines, including IL‐6, TNF‐α, and IL‐1β, in the serum of septic mice was detected by ELISA. (G, H) Flow cytometric analysis of the CD80+ immune cell population in the mouse spleen and quantitative analysis of CD80 levels. (I) H&E staining of liver, lung, and spleen tissues. Green arrows indicate central hepatic venous congestion; yellow arrows indicate necrotic hepatocytes; red arrows indicate neutrophil aggregation. Statistical significance was determined by one‐way ANOVA. ^*^p < 0.05, ^**^p < 0.01, ^***^p < 0.001; *n.s*. indicates no significant difference.

The aforementioned results indicate that ME@FDsi effectively disrupts the vicious cycle of neuroinflammation by targeting the pyroptosis pathway in brain microglia, thereby significantly ameliorating the typical symptoms of SAE. However, sepsis is fundamentally a fatal systemic inflammatory syndrome, and the uncontrolled systemic inflammatory response, along with subsequent multiple organ failure, also contributes to high mortality. Therefore, an ideal SAE treatment strategy should not be confined to the central nervous system but should also benefit systemic stability. Based on this, we further investigated the protective effects of ME@FDsi treatment on systemic inflammation and distal organs (such as the spleen, liver, and lung) in a septic mouse model. To evaluate the therapeutic efficacy against sepsis, septic mouse models (*n* = 10) were intravenously administered different drugs or PBS at 1 and 12 h after LPS injection (Figure 6A). To validate the efficient targeted therapeutic effect of the material, the experiment included an FDsi nanoparticle group; free drug groups (Fsi and DSF) were set to highlight the advantage of drug‐loaded therapy, with a PBS group serving as the control. Survival rate, clinical scores, and changes in body temperature and weight were recorded for 7 consecutive days. Most deaths occurred within the first 48 h, after which the LPS group had a survival rate of only 40%. (Figure 6B). Although mice treated with free drugs and FDsi nanoparticles exhibited prolonged survival, the overall survival rate remained low. In contrast, ME@FDsi nanoparticle treatment significantly increased the survival rate to 80%, corresponding to a 66.7% relative reduction in mortality. This substantial improvement demonstrates that ME@FDsi effectively rescued the majority of animals from lethal SAE challenge. Recording of body weight changes over 7 days (Figure S39) showed the most significant weight loss at 48 h. Weight recovered in both the free drug and FDsi treatment groups, but the recovery was most pronounced in the ME@FDsi treatment group. Recording of temperature changes over 7 days (Figure S40) showed that the body temperature of mice in the LPS group was approximately 35°C, with some individuals exhibiting a significant drop. Among the treatment groups, the ME@FDsi group recovered to above 36°C the fastest and maintained a stable state. These results suggest that ME@FDsi nanoparticles can effectively alleviate sepsis‐related complications associated with body temperature in the mouse model. Furthermore, clinical scores (Figure 6C) were highest in the LPS group, while scores decreased in mice treated with free drugs and FDsi. The ME@FDsi treatment group showed the most significant reduction in clinical scores, indicating improved physical condition.Blood and major organs (such as the spleen) were collected 24 h after LPS modeling for ELISA, qPCR, and flow cytometric analysis to validate the therapeutic effects. As shown in Figure 6D–F, pro‐inflammatory cytokines (such as IL‐6, TNF‐α, and IL‐1β) were significantly upregulated in the LPS‐treated group. When treated with free drugs (Fsi and DSF) and nanoparticles (FDsi and ME@FDsi), the expression of IL‐1β, IL‐6, and TNF‐α was inhibited, with ME@FDsi showing the best effect. To quantify the transcripts of inflammatory genes, we purified RNA from lung samples and conducted qPCR (Figure S41). The results showed that free drugs or nanoparticles effectively inhibited LPS‐induced *IL‐1β*, *IL‐6*, and *TNF‐α* protein expression. Macrophage phenotypes were analyzed by flow cytometry, with the gating strategy shown in Figure S42. As shown in Figure 6G,H, the PBS group had low levels of CD80+ macrophages in the spleen. In contrast, the LPS group showed a significant increase in CD80+ macrophages, indicating enhanced inflammatory response and increased M1‐type cell numbers. Compared to the LPS group, ME@FDsi nanoparticles reduced the proportion of CD80+ macrophages from 15.58 ± 0.563% to 10.45 ± 2.599%. The free drug groups showed a slight decrease in CD80+ cells, but these changes were not statistically significant. Subsequently, to more intuitively evaluate the therapeutic effect of ME@FDsi nanoparticles, we observed pathological changes in organs such as the liver, lung, and spleen using H&E staining (Figure 6I). Compared to healthy mice, the model group exhibited pathological features in the liver, including central venous congestion (green arrows) and hepatocyte necrosis (yellow arrows). These symptoms improved after nanoparticle treatment. Pathological features in the lungs showed thickened alveolar walls accompanied by neutrophil aggregation (red arrows), but no alveolar wall thickening or neutrophil aggregation was observed after nanoparticle treatment. Pathological features in the spleen manifested as blurred boundaries between the cortex and medulla. After nanoparticle treatment, no pathological changes were observed in the spleen tissue, consistent with the control group. These pathological findings from major organs provide substantial support for the restoration of organ structure and function following nanodrug treatment, strongly confirming the protective effect of ME@FDsi against multiple organ injury.

### 2.7 Biosafety

**FIGURE 7:**
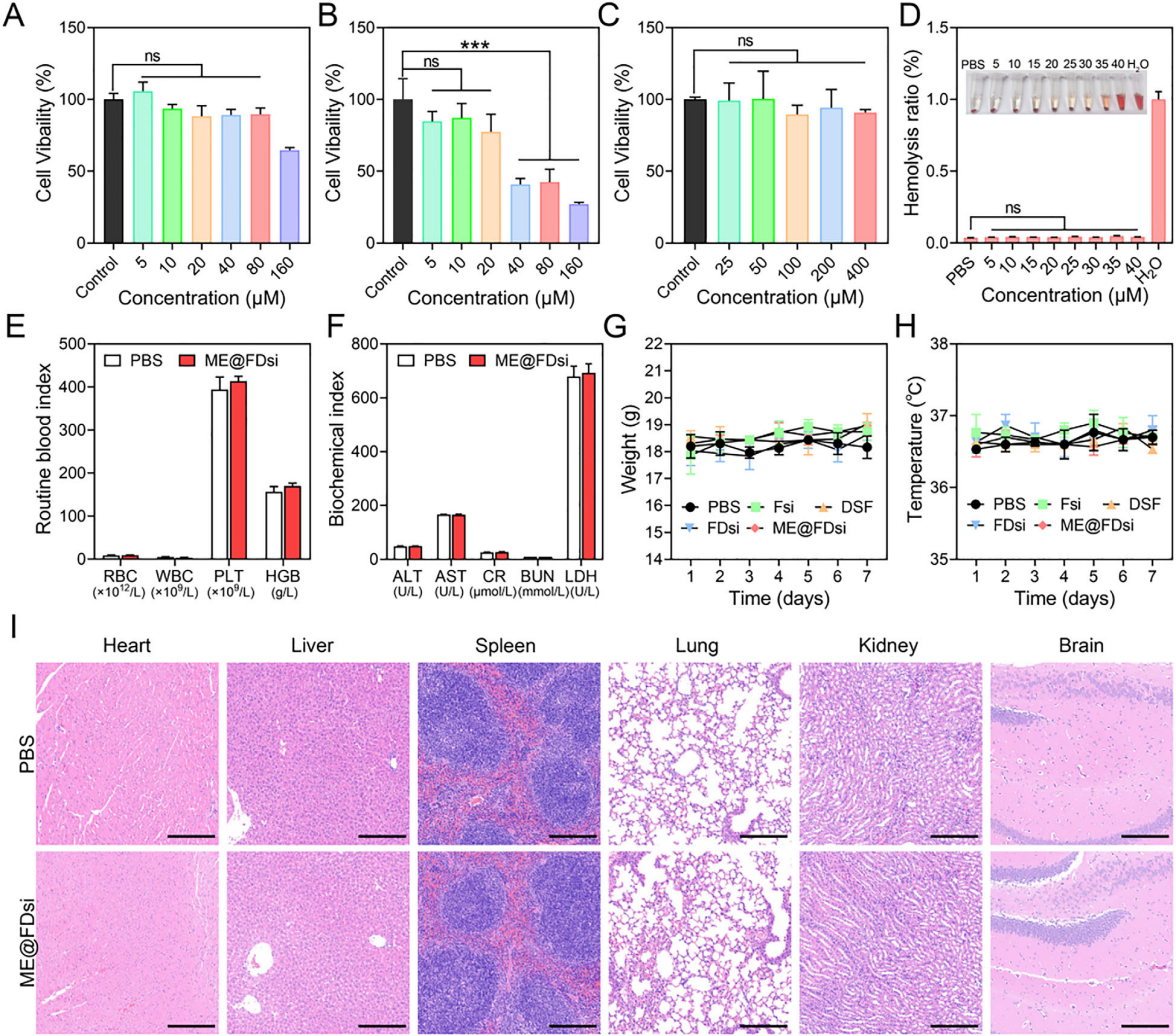
Biosafety Assessment. (A) Viability of BV2 cells (determined by CCK‐8 assay) after 24‐h incubation with different concentrations of ME@FDsi nanoparticles. (B) Viability of BV2 cells (determined by CCK‐8 assay) after 24‐h incubation with different concentrations of DSF. (C) Viability of BV2 cells (determined by CCK‐8 assay) after 24‐h incubation with different concentrations of Fsi. (D) Hemolysis analysis of fresh red blood cells after 24‐h co‐incubation with different concentrations of ME@FDsi. (E, F) Routine blood test parameters and plasma biochemical parameters of healthy mice 48 h after intravenous administration via the tail vein. (G) Body weight changes of healthy mice after intravenous administration. (H) Body temperature changes of healthy mice after intravenous administration. (I) H&E staining results of various organs from healthy mice 48 h after intravenous administration. Scale bar = 200 µm. Data are presented as mean ± standard deviation. Statistical analysis was performed using one‐way ANOVA. ^*^p < 0.05, ^**^p < 0.01, ^***^p < 0.001; *n.s*. indicates no significant difference.

To evaluate the biosafety and potential toxicity of ME@FDsi nanoparticles, we investigated their therapeutic potential through both in vitro and in vivo experiments. After co‐culturing ME@FDsi with BV2 cells for 24 h, no significant decrease in cell viability was observed even at a concentration of 80 µm (Figure 7A). In contrast, free DSF significantly inhibited cell activity at a concentration as low as 40 µm (Figure 7B), while free Fsi exhibited good biocompatibility (Figure 7C). The hemolytic activity of ME@FDsi nanoparticles was further verified by co‐culturing them with freshly collected red blood cells from healthy mice. The results showed no observable hemolysis after 24 h of incubation at room temperature (Figure 7D). Subsequently, we analyzed routine blood parameters, plasma biochemical markers, changes in body weight and temperature, and histopathological alterations in vital organs. Forty‐eight hours after administration, no significant differences were observed between the ME@FDsi nanoparticle group and the control group in terms of red blood cell (RBC) count, white blood cell (WBC) count, platelet (PLT) count, and hemoglobin (HGB) levels (Figure 7E). These results indicate that ME@FDsi nanoparticles do not cause damage to the hematopoietic system of healthy mice. Plasma biochemical markers, including alanine aminotransferase (ALT), aspartate aminotransferase (AST), creatinine (CRE), blood urea nitrogen (BUN), and lactate dehydrogenase (LDH), showed no significant abnormalities (Figure 7F). Body weight and temperature were monitored over 7 days, revealing no notable changes between the administered group and the control group (Figure 7G,H). Histopathological examination of key organs—including the heart, liver, spleen, lungs, kidneys, and brain—conducted 48 h after intravenous administration revealed no signs of tissue damage (Figure S43; Figure 7I). To further evaluate the potential off‐target effects of ME@FDsi on the central nervous system, we performed behavioral safety tests in healthy mice. Healthy mice were divided into three groups: the Control (PBS) group (healthy control), the LPS group (SAE model positive control), and the Control (ME@FDsi) group (healthy mice treated with ME@FDsi). Open field tests were conducted on days 1, 3, and 7 post‐administration to assess the time spent in the center zone. As shown in Figure S44, at all three time points, the center zone residence time of mice in the Control (ME@FDsi) group showed no significant difference compared with the Control (PBS) group. These results indicate that ME@FDsi has no significant effects on anxiety‐like behavior in healthy mice, exhibits no off‐target behavioral toxicity, and its therapeutic effects are pathologically state‐selective. In summary, ME@FDsi demonstrated favorable biosafety both in vitro and in vivo.

## 3 Discussions

The ME@FDsi nanosystem developed in this study represents an advanced therapeutic strategy for SAE, with its core advantages manifested at multiple levels. First, the system ingeniously integrates various functional modules through biomimetic design: it utilizes tetrahedral framework nucleic acid (tFNA) as a drug carrier to achieve efficient loading and protection of both disulfiram (DSF) and siTNF‐α; employs erythrocyte membrane coating to significantly enhance nanoparticle stability, prolong blood circulation time, and reduce immunogenicity; and notably, achieves specific targeting of M1 microglia through MG1 peptide modification, greatly increasing drug accumulation at the lesion site. Second, the platform demonstrates multiple synergistic therapeutic mechanisms: DSF effectively inhibits GSDMD‐mediated pyroptosis, siTNF‐α targets and suppresses a key inflammatory cytokine, while tFNA itself possesses inherent anti‐inflammatory and antioxidant activities. Together, these components work synergistically to comprehensively block the vicious cycle of neuroinflammation. In terms of experimental validation, the therapeutic efficacy of ME@FDsi was not only statistically significant but also demonstrated profound clinical relevance. The 40% absolute improvement in survival is particularly noteworthy, as SAE carries a mortality rate of 50%–70% in ICU settings with no approved disease‐modifying therapies currently available. In cognitive recovery, ME@FDsi‐treated mice exhibited a 4.6‐fold improvement in the open field test and achieved 68% of healthy control performance in novel object recognition—a magnitude of cognitive rescue rarely reported in SAE models. Importantly, these improvements were sustained for at least 14 days, with effect sizes at day 14 comparable to those at day 7, distinguishing ME@FDsi from conventional symptomatic interventions and supporting its potential as a disease‐modifying therapy. The consistency of therapeutic benefits across molecular, cellular, histological, behavioral, and systemic levels further reinforces the reliability of these findings, while its favorable biosafety profile underscores its translational potential.

However, the study also has several noteworthy limitations. Regarding the disease model, it primarily relies on the LPS‐induced sepsis model. While convenient and reproducible, this model does not fully recapitulate the complex pathophysiological processes in clinical sepsis patients, particularly lacking the real‐world context of bacterial infection and comprehensive immune system activation. Concerning targeting specificity, the study found that the MG1 peptide also exhibits some binding affinity for the macrophage cell line (Raw 264.7). This off‐target effect could lead to drug distribution in non‐target tissues, potentially impacting therapeutic efficacy and increasing the risk of unforeseen side effects. Furthermore, the assessment of the nanosystem's long‐term toxicity and immunogenicity is insufficient, lacking safety data following repeated administration, which is crucial for clinical translation. Finally, although cognitive improvement was demonstrated, the specific mechanisms of drug passage across the blood‐brain barrier and its pharmacokinetics within brain tissue require more in‐depth investigation, necessitating further refinement in subsequent studies.

In summary, the ME@FDsi nanosystem provides an innovative solution for SAE treatment, but its clinical translation still faces numerous challenges. More profound research is required in areas such as model representativeness, targeting specificity, and long‐term safety.

## 4 Conclusion

This study successfully developed biomimetic ME@FDsi nanoparticles. By modifying erythrocyte membrane vesicles with the MG1 peptide, the nanoparticles exhibit highly specific targeting capability toward M1‐type microglia, enabling efficient drug delivery to the brain. This system effectively regulates oxidative stress and inflammatory responses, providing a novel strategy for treating SAE. Systematic in vitro studies elucidated the mechanism of action of ME@FDsi nanoparticles, confirming their ability to enhance drug internalization, alleviate damage by scavenging intracellular reactive oxygen species (ROS)‐induced oxidative stress, and inhibit cellular pyroptosis. Concurrently, ME@FDsi nanoparticles can reduce microglial polarization toward the M1 phenotype, decrease the production of pro‐inflammatory factors, and suppress the inflammatory storm. The erythrocyte membrane coating endows the nanoparticles with superior pharmacokinetic properties and enhances their targeting and penetration capabilities toward brain injury sites. In a mouse model of severe sepsis induced by LPS, administration of ME@FDsi nanoparticles significantly increased the survival rate, improved clinical scores and body weight/temperature parameters, and alleviated organ damage. Furthermore, tBV2 cells were dispensed into 6‐well plates at a seeding density of 5 × 10⁵ cells/well.hese nanoparticles exhibited promise in ameliorating SAE outcomes, along with reprogramming microglial phenotypes, reconstituting the immune landscape, and improving murine learning and memory capabilities. This study highlights the significant potential of ME@FDsi nanoparticles as a breakthrough therapy for SAE, promising to substantially improve patient prognosis.

## 5 Experiment Section

### 5.1 Reagents

A comprehensive list of abbreviations used in this study is provided in Table S1. All single‐stranded DNA sequences (see Table S2 for specifics), purified by High‐Performance Liquid Chromatography (HPLC), were synthesized by Sangon Biotech (Shanghai, China). Cell culture‐related reagents, including Dulbecco's Modified Eagle Medium (DMEM), Fetal Bovine Serum (FBS), penicillin/streptomycin, and Phosphate‐Buffered Saline (PBS), were purchased from Gibco Life Technologies. Disulfiram (Catalog no.97‐77‐8), PEG‐300 (Catalog no.25322‐68‐3), and Tween 80 (Catalog no.9005‐65‐6) were obtained from MedChemExpress. Nigericin was purchased from Macklin, and Lipopolysaccharides (LPS) (Catalog no.L2880) were sourced from Sigma–Aldrich. The DiI fluorescent dye, BCA assay kit, Coomassie Brilliant Blue, RIPA lysis buffer, Lactate Dehydrogenase (LDH) (Catalog no.C0017) Cytotoxicity Assay Kit, Cell Counting Kit‐8 (CCK‐8), Cell Apoptosis & Necrosis Assay Kit, and DAPI were all acquired from Beyotime Biotechnology. Phenylmethylsulfonyl fluoride (PMSF), Trypsin‐EDTA solution, and 4% Paraformaldehyde were purchased from Solarbio. SDS‐PAGE gels were obtained from Genscript Biotech. Protein Loading Buffer was sourced from Coolaber. Mouse TNF‐α (Catalog no.1217202), IL‐1β (Catalog no.1210122), and IL‐6 ELISA kits (Catalog no.1210602) were purchased from Dakewe Biotech. The RNA Reverse Transcription Kit (Catalog no.11141ES60) and quantitative PCR (qPCR) kit (Catalog no.11184ES08) were obtained from Yeasen Biotechnology. Triton X‐100 was acquired from Biosharp. The mouse anti‐mouse β‐Actin antibody (RRID:AB_3718569), the rabbit anti‐mouse GSDMD (RRID:AB_3718567) and rabbit anti‐mouse Cleaved‐GSDMD (RRID:AB_3718568) antibodies were purchased from Cell Signaling Technology. HRP‐conjugated anti‐rabbit IgG and anti‐mouse IgG secondary antibodies were also purchased from Cell Signaling Technology. The Precision Plus Protein Dual Color Standard was obtained from Bio‐Rad.

### 5.2 Cell Lines and Cell Culture

The mouse microglial cell line (BV‐2) (RRID:CVCL_0182), and the mouse brain microvascular endothelial cell line (bEnd.3) (RRID:CVCL_0170) were all purchased from the iCell Bioscience Inc in May 2024. The mouse macrophage cell line (RAW 264.7) (RRID:CVCL_0493), and human umbilical vein endothelial cell line (HUVEC) (RRID:CVCL_B6YK)were all purchased from the American Type Culture Collection (ATCC). HUVECs were cultured in DMEM medium supplemented with 10% fetal bovine serum and 1% penicillin/streptomycin, and maintained in a sterile, humidified incubator at 37°C with 5% CO_2_. BV2 and RAW 264.7 cells were cultured in DMEM medium supplemented with 10% heat‐inactivated fetal bovine serum, 1% penicillin/streptomycin, 1% Glutamine, and 1% Sodium Pyruvate, under the same incubation conditions (37°C, 5% CO_2_). Cells were passaged when they reached approximately 70%–80% confluence. All cell lines used in this study were routinely tested for mycoplasma contamination using the GMyc‐PCR Mycoplasma Detection Kit, following the manufacturer's protocol, with consistently negative results (Figure S45).

### 5.3 Erythrocyte Membrane Extraction

Whole blood was collected from anesthetized C57 mice via the orbital plexus and centrifuged (3000 rpm, 5 min). The supernatant (primarily plasma) was discarded, and the red pellet was washed with 1 × PBS until the supernatant became colorless. The red blood cells were then subjected to hypotonic lysis to rupture the erythrocyte membranes, followed by centrifugation at 4°C (10 000 rpm, 15 min). This washing step was repeated twice. The resulting pink erythrocyte membrane pellets were collected and stored at ‐80°C for future use. Prior to use, the membrane pellets were resuspended in PBS and subjected to mild sonication. The membrane protein concentration was determined using the standard Bicinchoninic Acid (BCA) assay.

### 5.4 Preparation of ME@FDsi

Component oligonucleotides (single‐stranded DNA and double‐stranded siRNA) were mixed at a stoichiometric ratio and dissolved in annealing Tris‐Magnesium Sulfate Buffer (containing 10.0 mm Tris, 5.0 mm MgCl_2_, pH 8.0, Beyotime, China) to a final concentration of 1.0 µm. The solution was heated to 95°C for 2 min, then slowly cooled to room temperature and incubated at 4°C overnight. For FDsi synthesis: Component oligonucleotides (single‐stranded DNA and double‐stranded siRNA) were mixed at a stoichiometric ratio and dissolved in annealing Tris‐Magnesium Sulfate Buffer (containing 10.0 mm Tris, 5.0 mm MgCl_2_, pH 8.0, Beyotime, China) to a final concentration of 1.0 µm. This was followed by heating (95°C, 10 min) and cooling (4°C, 20 min). The next step involved the complexation of DSF with Fsi and the preparation of DSPE‐PEG‐MG1. Different concentrations of DSF (20, 40, 80, 160, 320 µm) were mixed with Fsi (200 nm) and vortexed for 6 h. Residual single‐stranded DNA and unbound DSF were subsequently removed by ultrafiltration (30 kDa molecular weight cut‐off filters, Merck Millipore, USA). After dissolving the PEG8‐MG1 peptide, TCEP was added and co‐incubated at 37°C for 30 min, followed by the addition of DSPE‐PEG2000‐Mal for the conjugation reaction at 37°C with stirring in the dark for 2 h. Unreacted impurities were removed using a dialysis bag to obtain DSPE‐PEG‐MG1. The extracted erythrocyte membrane vesicles were then co‐incubated with FDsi and extruded 15 times through polycarbonate membranes with pore sizes of 800, 400, and 200 nm sequentially to obtain NE‐FDsi. Finally, DSPE‐PEG‐MG1 was co‐incubated with NE‐FDsi for 30 min to obtain ME@FDsi.

### 5.5 Characterization

The successful synthesis of Fsi was verified by agarose gel electrophoresis. The hydrodynamic diameter and zeta potential of Fsi, FDsi, and ME@FDsi were measured using a particle size and zeta potential analyzer (Litesizer 500, Anton Paar). The size and morphology of the nanoparticles were characterized using transmission electron microscopy (Tecnai G2 20 TWIN, FEI). SDS‐PAGE analysis was performed using a multifunctional gel imaging system (Tanon MINI Space 2000). Western blot bands were developed using a chemiluminescence imaging system (ImageQuant 800, Amersham). Flow cytometry analysis was conducted using an Attune NxT flow cytometer (Invitrogen, Thermo Fisher Scientific). Absorbance values for the CCK‐8 assay were measured using a microplate reader (iMark, Bio‐Rad).

### 5.6 In Vitro Release Study

For the release experiment, a dialysis membrane (30 kDa, Solarbio, Beijing, China) was used to separate the release medium, PBS (0.01 mol L^−^^1^, pH 7.4), into an inner compartment (3 mL) and an outer compartment (30 mL). FDsi (equivalent to 40 µm DSF) was dissolved in the inner compartment. The entire system was placed in an environment maintained at 37°C. The amount of DSF released into the outer compartment was measured by determining the OD value at a wavelength of 280 nm at multiple time points.

### 5.7 Cytotoxicity Evaluation

The cytotoxicity of free DSF, free Fsi, and ME@FDsi nanoparticles against BV2 cells was assessed using the CCK‐8 assay, performed as follows: BV2 cells were seeded in a 96‐well plate at a density of 8000 cells per well and cultured overnight. The cells were incubated in the presence of a range of drug concentrations over a 24‐hour period. Subsequently, 10 µL of CCK‐8 reagent was added to each well, followed by further incubation for 2 h. The optical density (OD) was measured at a wavelength of 450 nm using a microplate reader to determine cell viability. The cell survival rate (%) was calculated using the formula: (OD_sample—OD_blank) / (OD_control—OD_blank) × 100.

### 5.8 In Vitro Blood‐Brain Barrier (BBB) Permeability Assay

bEnd.3 cells were seeded into the upper chamber (0.4 µm pore size) of a 24‐well Transwell plate (Corning, USA) at a density of 1 × 10^4^ cells per insert to construct an in vitro BBB model. When the cells reached 80% confluence, they were stimulated with 1 µg/mL LPS for 24 h. Subsequently, Cy3‐labeled Fsi, FDsi, and ME@FDsi were added to the upper chamber. The fluorescence intensity in the lower chamber was measured using a fluorescence spectrometer at 2, 6, and 12 h post‐addition.

### 5.9 Cellular Uptake of Nanoparticles

To monitor the precise uptake of MG1 peptide‐modified ME@FDsi by M1‐type microglia, BV2 cells were differentiated into M1 microglia using LPS (1 µg/mL) and into M2 microglia using IL‐4 (20 ng/mL), with an untreated group serving as the control. Cy3‐labeled E‐FDsi and ME@FDsi were added to the differently treated cells. After 4 h of incubation, the proportion of fluorescently labeled cells to the total cell count was determined using a flow cytometer (Agilent Attune NxT). Separately, BV2 cells were seeded into glass‐bottom dishes at a density of 3 × 10^5^ cells per dish and cultured for 24 h. The old medium was discarded and replaced with medium containing Cy5‐labeled E‐FDsi and ME@FDsi nanomaterials, followed by another 4 h of co‐culture. The cells were then collected, stained with Hoechst staining reagent for 30 min, and observed under a fluorescence microscope to visualize the Cy5 and Hoechst staining.

### 5.10 Immune Evasion

To evaluate the immune evasion capability of the nanoparticles, we prepared Cy5‐labeled ME@FDsi. First, BV2 cells were seeded in glass‐bottom dishes at a density of 3 × 10^5^ cells per dish and cultured for 24 h at 37°C with 5% CO_2_. The BV2 cells were then stimulated with LPS to simulate an inflammatory response. ME@FDsi was added to the LPS‐induced cells and incubated for either 2 or 8 h. After treatment, the cells were washed twice with PBS, stained with Lysotracker Green for lysosomes, and examined by confocal laser scanning microscopy.

### 5.11 Western Blot Analysis

BV2 cells were dispensed into 6‐well plates at a seeding density of 5 × 10^5^ cells per well. After 20 h of culture, the cells were treated with LPS (1 µg/mL). One hour later, the cells were respectively treated with PBS, Fsi, DSF, FDsi, or ME@FDsi. After 24 h, the cells were collected and resuspended in 100 µL of lysis buffer. The levels of protein were assessed utilizing the bicinchoninic acid (BCA) assay kit. Then, 20 µg of protein samples were separated by SDS‐PAGE and transferred onto a PVDF membrane. The membrane was blocked with 5% skim milk for 1 h, followed by overnight incubation at 4°C with anti‐GSDMD (1:1000) and anti‐N‐GSDMD (1:1000) antibodies, respectively. Subsequently, the membrane was incubated with an HRP‐conjugated secondary antibody (1:10000 dilution) at room temperature for 1 h. Finally, bands were visualized using ECL reagent, analyzed with ImageJ software, and normalized to β‐actin as an internal' control.

### 5.12 ELISA

The levels of IL‐1β, IL‐6, and TNF‐α in BV2 cell culture supernatants were quantified using mouse‐specific kits according to the manufacturer's instructions. The concentrations of IL‐1β, IL‐6, and TNF‐α in serum and brain tissues were measured using corresponding mouse‐specific kits, also following the manufacturer's protocols. To detect IL‐1β, IL‐6, and TNF‐α, samples and standards were added to the wells of the microplate, followed by the addition of biotinylated antibodies. The plate was incubated at 37°C to facilitate antigen‐antibody binding. After washing with buffer, the plate was incubated with streptavidin‐HRP at 37°C. Following another wash, the color was developed by adding a 3,3',5,5'‐Tetramethylbenzidine (TMB) substrate solution. The reaction was stopped by adding the stop solution, and the absorbance was measured at 450 nm using a microplate reader (iMark, Bio‐Rad).

### 5.13 Reactive Oxygen Species (ROS) Level Detection

Intracellular ROS levels were measured using a DCFH‐DA assay kit. The specific procedure was as follows: BV2 cells were seeded in 6‐well plates at a density of 5 × 10^5^ cells per well. After 20 h of culture, the cells were treated with LPS (1 µg/mL). One hour later, the cells were respectively treated with PBS, Fsi, DSF, FDsi, or ME@FDsi. After 24 h of treatment, the cells were washed three times with PBS. The cells from each group were then incubated with DCFH‐DA solution for 20 min, washed three times with culture medium, and ROS fluorescence images were captured using a microscope. Additionally, specific fluorescence intensity data were obtained by flow cytometry.

### 5.14 Quantitative Real‐Time PCR (qRT‐PCR) Protocol

Total RNA was first dissolved using TRIzol reagent and purified using an RNA purification kit. cDNA was obtained following standard procedures. The expression levels of target mRNAs were detected using SYBR Green I premix on a qRT‐PCR analysis system (QuantStudio 7, Thermo Fisher Scientific, USA). The expression of all target mRNAs was normalized to GAPDH as an internal control (details provided in Table S3).

### 5.15 In Vivo Small Animal Imaging

E‐FDsi and ME@FDsi were labeled with the fluorescent dye Cy5. Subsequently, Cy5‐labeled NE‐FDsi, ME@FDsi, and free Cy5 were injected into SAE mice via the tail vein. Fluorescence images of the brain were captured at different time points (6, 12, and 24 h) using a full‐spectrum in vivo imaging system (AniView Phoenix).

### 5.16 Animal Model Establishment and Experimental Treatment

Eight‐week‐old male C57BL/6J mice (purchased from Baishtong Biotechnology Co., Ltd.) were used in this study. All experimental procedures were approved by the Animal Ethics Committee of Shenzhen Longgang Central Hospital (Approval No.: NO.2024‐012). The mice were randomly divided into six groups: Control group, LPS group, LPS + Fsi group, LPS + DSF group, LPS + FDsi group, and LPS + ME@FDsi group. At 1 and 12 h after LPS modeling, normal saline, Fsi, DSF, FDsi, and ME@FDsi were administered via tail vein injection, respectively. Finally, heart, liver, spleen, lung, kidney, and brain tissues were collected. Hematoxylin and eosin (H&E) staining was performed on all collected tissues. Additionally, Iba1 immunofluorescence staining and Nissl staining were conducted specifically on brain tissues. Serum samples were also collected to determine cytokine levels.

### 5.17 Flow Cytometry Analysis

At the end of the experiment, spleen and brain tissues were harvested and minced into small pieces. The tissues were then digested with 1500 U/mL collagenase, 1000 U/mL hyaluronidase, and DNase under continuous shaking at 37°C for 40 min. The process began with a filtration step, where a 70 µm nylon mesh was used to retrieve single cells, which were subsequently subjected to two rounds of PBS washing. The isolated single cells were blocked with 1% BSA at 4°C in the dark for 30 min. Subsequently, splenocytes were stained with a viability dye, anti‐F4/80‐FITC (1:100), and anti‐CD80‐APC (1:100) antibodies on ice in the dark for 30 min. Brain cells were stained with a viability dye, anti‐F4/80‐FITC (1:100), and anti‐CD80‐APC (1:100) antibodies under the same conditions. Finally, the cells were washed, resuspended in PBS staining buffer containing 1% fetal bovine serum, and analyzed using a flow cytometer (Attune NxT, Agilent). Data analysis was performed using FlowJo software.

### 5.18 Section Staining and Immunofluorescence Experiments

After anesthesia, the heart, liver, spleen, lung, kidney, and brain tissues were collected from the experimental mice. The tissues were fixed in 10% formalin, dehydrated through an ethanol series, and embedded in paraffin. Sections were then prepared and subjected to Hematoxylin and Eosin (H&E) staining. Additionally, brain tissue sections underwent Nissl staining and Iba‐1 immunofluorescence staining. Finally, the stained sections were observed under a fluorescence microscope, and images were analyzed using Image‐Pro Plus 16.0 software.

### 5.19 In Vivo Biosafety Assessment

Healthy C57 mice were treated with PBS, Fsi, DSF, FDsi, or ME@FDsi. Body temperature and body weight of the mice in each group were monitored for 7 days. Blood was collected for routine hematological tests, and organs, including the heart, liver, spleen, lungs, kidneys, and brain, were harvested for Hematoxylin and Eosin (H&E) staining analysis.

### 5.20 Statistical Analysis

Quantitative data are presented as the mean ± standard deviation (SD) from three or more independent experiments. One‐way analysis of variance (ANOVA) was used for comparisons among multiple groups. All statistical analyses were performed using GraphPad Prism software (version 8). Statistical significance is denoted by asterisks: ^*^p < 0.05; ^**^p < 0.01; ^***^p < 0.001; *n.s*. indicates no significant difference.

## Conflicts of Interest

The authors declare no conflicts of interest.

## Supporting information




**Supporting File**: advs75387‐sup‐0001‐SuppMat.docx.

## Data Availability

The data that support the findings of this study are available from the corresponding author upon reasonable request.
