# Supplementary material for: Microglia‐Targeted Biomimetic Tetrahedral Framework Nucleic Acid Nanovesicles for Synergistic Treatment of Sepsis‐Associated Encephalopathy
Source: Adv Sci (Weinh). 2026 Apr 27;13(40):e23716. doi: 10.1002/advs.202523716 (PMC13335498; doi:10.1002/advs.202523716)
Supplement: Supplementary file 1 — Supporting File: advs75387‐sup‐0001‐SuppMat.docx. [file ADVS-13-e23716-s001.docx]

Supporting Information

**Microglia-Targeted Biomimetic Tetrahedral Framework Nucleic Acid Nanovesicles for Synergistic Treatment of Sepsis-Associated Encephalopathy**

*Huimin Shi^‡^, Qiuxia Gao^‡^, Wenying Wang^‡^, Bin Li^‡^, Yukun* *Chen,* *Zhijun Yao, Yujie Li, Junrui Li, Na Li, Gong Gu, Zhimin Hou, Mengyuan Yang, Ruilin Zhang*, Hongju Yang*, Yuhui Liao*, Hongyi Lei**

H. Shi, Y. Chen, Z. Yao, H. Lei

Department of Anesthesiology

Shenzhen Clinical College (Longgang Central Hospital of Shenzhen)

Guangzhou University of Chinese Medicine

Shenzhen, Guangdong, 510006, China

E-mail: leihongyi2012@163.com

Q. Gao, W. Wang, J. Li, N. Li, G. Gu, Z. Hou, M. Yang, Y. Liao

Institute for Engineering Medicine

Kunming Medical University

Kunming, Yunnan, 650500, China

E-mail: liaoyh8@mail.sysu.edu.cn

B. Li

School of Inspection

Ningxia Medical University

Yinchuan, Ningxia, 750004, China

Y. Li

Department of Anesthesiology

Southwest Hospital

Third Military Medical University (Army Medical University)

Chongqing, 400000, China

1. Zhang

Institute for Engineering Medicine

NHC Key Laboratory of Drug Addiction Medicine

Kunming Medical University

Kunming, Yunnan, 650500, China

1. mail: zhangruilin@kmmu.edu.cn
2. Yang

Geriatric Medical Center

Division of geriatric Gastroenterology

The First Affiliated Hospital of Kunming Medical University

Kunming, Yunnan, 650032, China

E-mail: yanghongju@kmmu.edu.cn

‡These authors contributed equally to this work.

**Table S1.** Comprehensive list of abbreviations used in this study.

| **Abbreviation** | **Full Name** |
| --- | --- |
| DSF | disulfiram |
| siTNF-α | small interfering RNA targeting tumor necrosis factor-alpha |
| tFNA | tetrahedral framework nucleic acid |
| Fsi | tFNA/siTNF-α complex |
| FDsi | tFNA/siTNF-α complex loaded with disulfiram |
| EM | erythrocyte membrane |
| E@FDsi | erythrocyte membrane-coated FDsi (without MG1 peptide modification) |
| MG1 | M1 microglia-targeting peptide (sequence: CHHSSSAR) |
| ME@FDsi | MG1 peptide-modified erythrocyte membrane-coated FDsi |
| Mix | simple mixture of DSF, siTNF-α, tFNA, and erythrocyte membrane vesicles (unassembled) |

**Table S2.** DNA sequences for preparation of Fsi-siTNF-α .The underlined capital

letters represent a 2'-O-methyl base. All nucleic acids have a phosphodiester backbone.

| **Name** | **Sequences (5'-3')** |
| --- | --- |
| S1 | GTCTGAGGCAGTTGAGAGATCTCGAACATTCC |
| S2 | TAAGTCTGAAGATCCATTTATCACCAGCTGCTGCACGCCA  TAGTAGACGTATCACCTGTCC |
| S3 | AGCTACTTGCTACACGAGGATCTTCAGACTTAGGAATGT  TCGAGATCACATGCGAGGACTCGGTCCAATACCGTACTAACG  ATTACAGATCAA |
| S4 | CAGCTGGTGATAAAACGTGTAGCAAGTAGCTTTGATCTG  TAATCGACTCTACGGGAAGAGC-overhang_1_ |
| S5 | ATGCCCATCCGGCTCACTACTATGGCGTGCAG-overhang_1_ |
| S6 | CGAGTCCTCGCATGACTCAACTGCCTCAGACGGACAGG  TGATACGAGAGCCGGATGGGCATGCTCTTCCCGTAGAGATAG  TACGGTATTGGAC-overhang_1_ |
| Cy5-S1 | GTCTGAGGCAGTTGAGAGATCTCGAACATTCC-Cy5 |
| Cy3-S1 | GTCTGAGGCAGTTGAGAGATCTCGAACATTCC-Cy3 |
| TNF-α siRNA  sense | GUCUCAGCCUCUUCUCAUUCCUGCT |
| TNF-α siRNA  antisense | AGCAGGAAUGAGAAGAGGCUGAGACAU-overhang_1-1_ |
| Overhang_1_ | TTA TTA AGT AAC GTC TAG CCC |
| Overhang_1-1_ | GGG CTA GAC GTT ACT TAA TAA |

**Table S3.** Primers sequences of housekeeping gene GAPDH and relevant targeted genes for qRT-PCR.

| **mRNA** | **Primer (5’-3’)** | **Sequence** |
| --- | --- | --- |
| GAPDH | Forward | AACGACCCCTTCATTGAC |
|  | Reverse | TCCACGACATACTCAGCAC |
| TNF-α | Forward | ATGTCTCAGCCTCTTCTCATTC |
|  | Reverse | GCTTGTCACTCGAATTTTGAGA |
| IL-6 | Forward | CTCCCAACAGACCTGTCTATAC |
|  | Reverse | CCATTGCACAACTCTTTTCTCA |
| IL-1β | Forward | GCAACTGTTCCTGAACTCAACT |
|  | Reverse | ATCTTTTGGGGTCCGTCAACT |


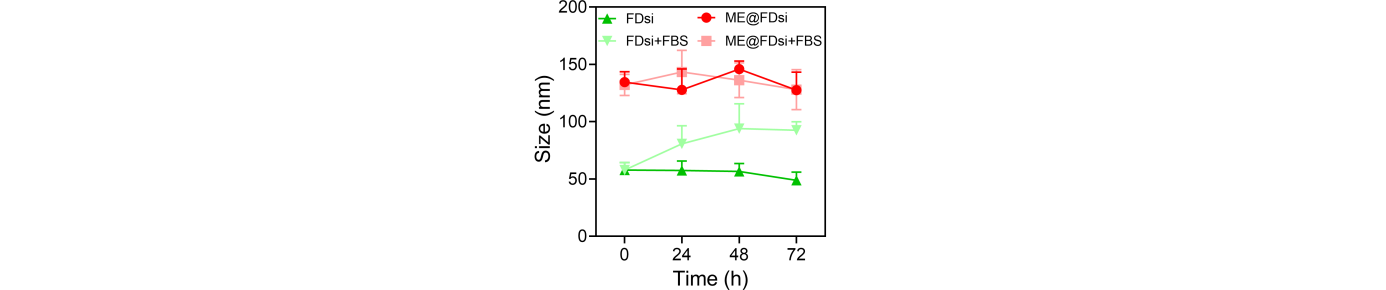


**Figure S1.** Evaluation of the serum stability of FDsi and ME@FDsi.


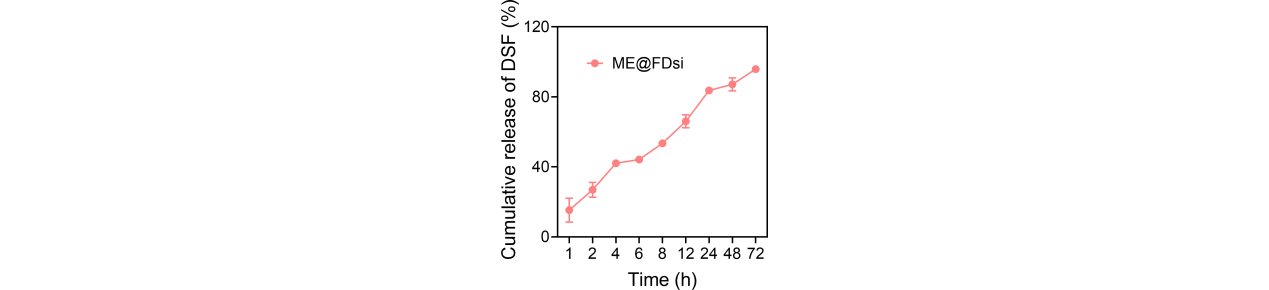


**Figure S2.** *In vitro* release profile of FDsi from ME@FDsi in PBS (pH 7.4, 37°C).


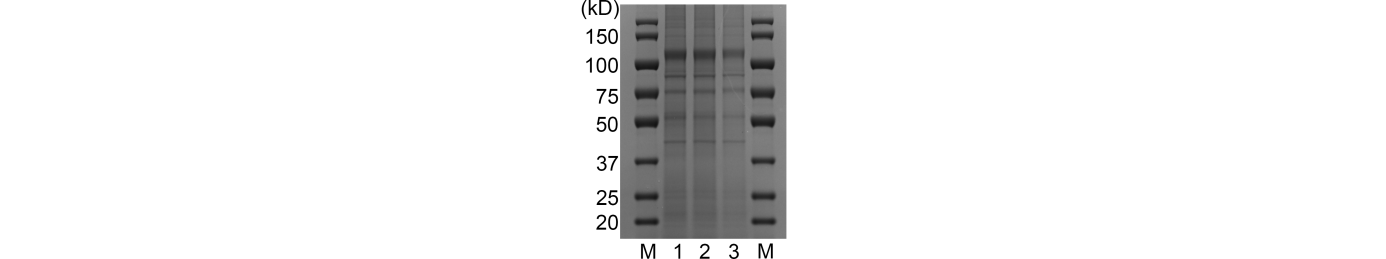


**Figure S3.** Coomassie Brilliant Blue SDS-PAGE gel. M, Marker; Lane 1, RBC membrane only; Lane 2, E@FDsi; Lane 3, ME@FDsi. The image demonstrates the similarity between the RBC membrane and ME@FDsi.


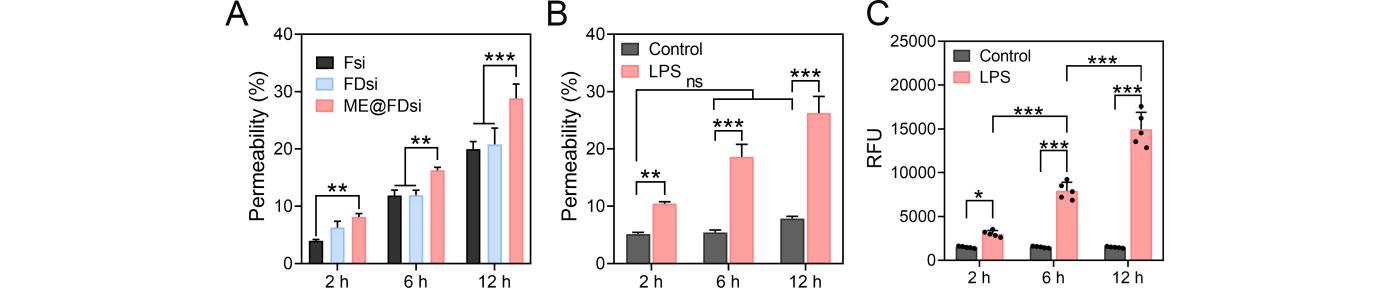


**Figure S4. (A)** An *in vitro* blood-brain barrier (BBB) model was constructed using bEnd.3 cells. When the cells reached 80% confluence, they were stimulated with 1 µg/mL LPS for 24 hours. Subsequently, Cy3-labeled Fsi, FDsi, and ME@FDsi were added to the upper chamber of the Transwell system. The fluorescence intensity in the lower chamber was measured using a fluorescence spectrometer at 2, 6, and 12 hours to evaluate the BBB penetration ability of the vesicles. **(B)** An *in vitro* blood-brain barrier (BBB) model was established using bEnd.3 monolayers cultured in Transwell inserts. The monolayers were treated with either PBS (intact BBB group) or 1 µg/mL lipopolysaccharide (LPS) for 24 h to simulate BBB injury associated with sepsis-associated encephalopathy (SAE). Subsequently, Cy3-labeled ME@FDsi was added to the upper chamber, and the fluorescence intensity in the lower chamber was measured using a fluorescence spectrophotometer at 2, 6, and 12 h post-addition. **(C)** *In vivo* brain accumulation of DiD-ME@FDsi under intact and disrupted BBB conditions. Healthy mice (intact BBB) and LPS-treated mice (disrupted BBB) were intravenously injected with DiD-ME@FDsi. Brain tissues were collected at 2, 6, and 12 h post-injection, homogenized, and fluorescence intensity was measured using a microplate reader (Ex/Em: 644/665 nm). Data are presented as mean ± standard deviation (SD) (*n* = 3). Statistical significance was calculated by one-way ANOVA. **p* < 0.05, ***p* < 0.01, ****p* < 0.001, *n.s.* indicates no significant difference.


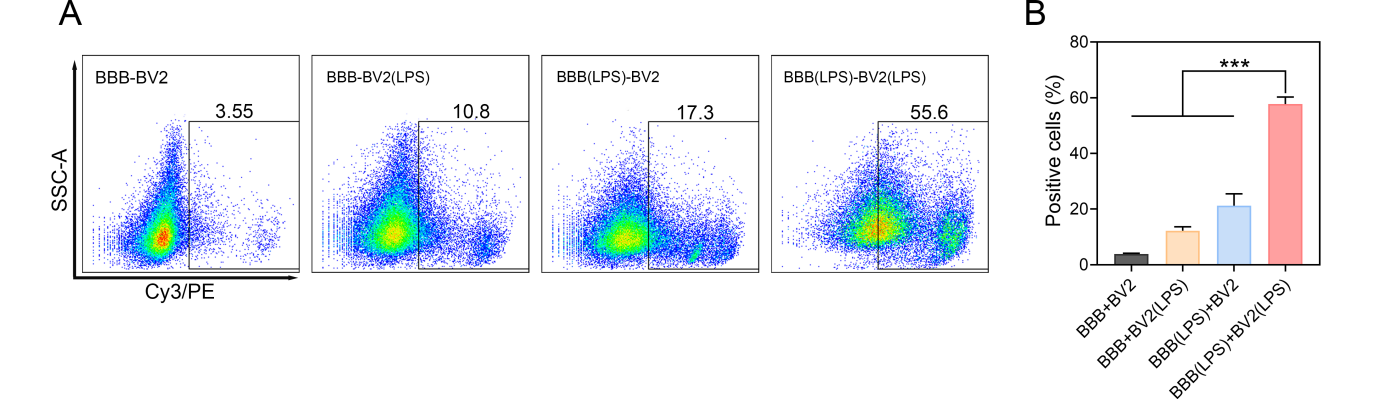


**Figure S5.** bEnd.3 monolayers were cultured in the upper chamber of the Transwell system and pretreated with either PBS (intact BBB group) or 1 µg/mL LPS (disrupted BBB group) for 24 h. BV2 cells in the lower chamber were pretreated with either PBS (resting state) or 1 µg/mL LPS (M1-polarized state) for 24 h. Subsequently, ME@FDsi was added to the upper chamber. After 12 h of co-incubation, BV2 cells were collected from the lower chamber and analyzed by flow cytometry. Data are presented as mean ± standard deviation. Statistical analysis was performed using one-way ANOVA. **p* < 0.05, ***p* < 0.01, ****p* < 0.001, *n.s.* indicates no significant difference.


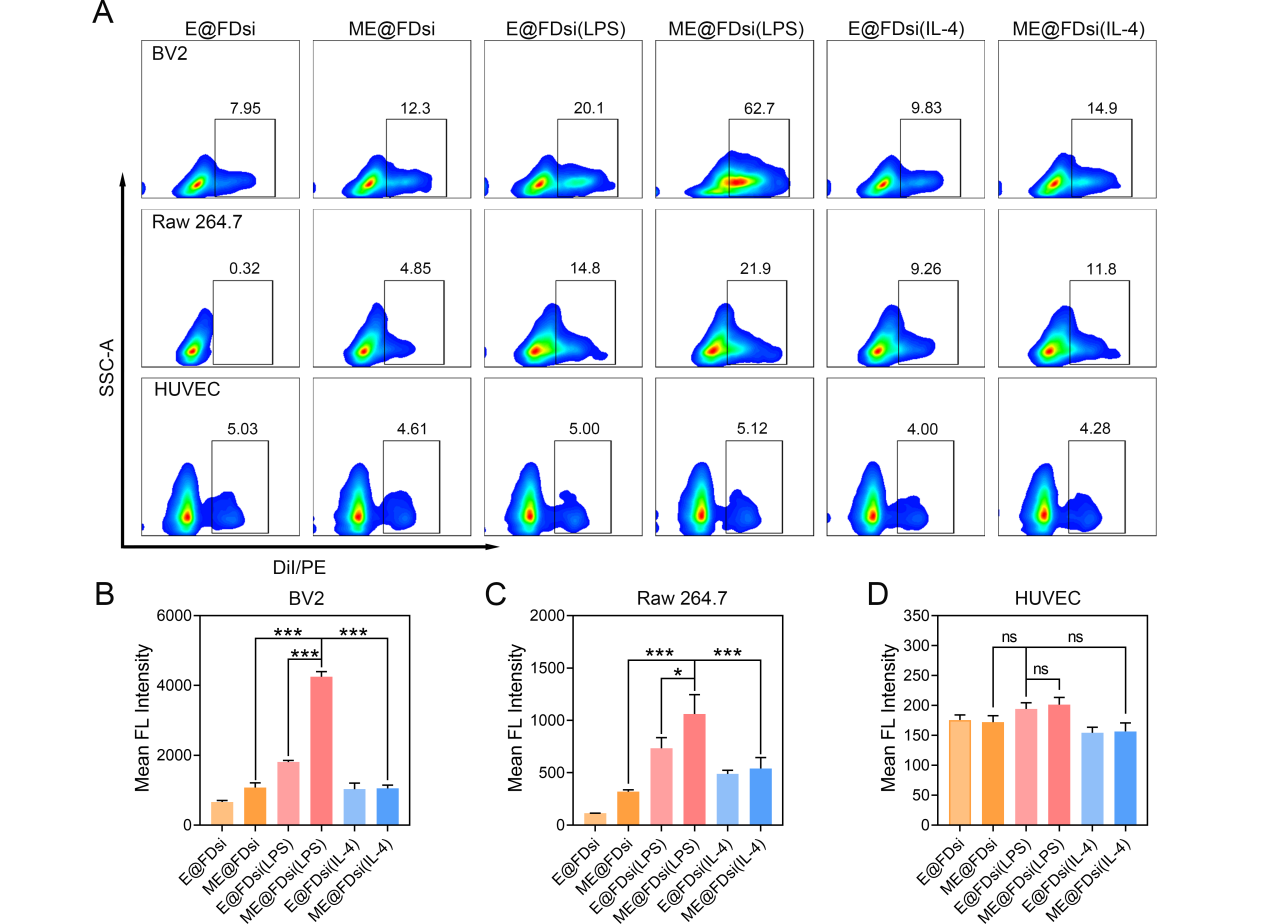


**Figure S6.** Uptake of DiL-labeled E@FDsi and ME@FDsi was detected by flow cytometry in BV2 cells, Raw 264.7 cells, and HUVEC cells treated with PBS, LPS, or IL-4. Data are presented as mean ± standard deviation. Statistical analysis was performed using one-way ANOVA. **p* < 0.05, ***p* < 0.01, ****p* < 0.001, *n.s.* indicates no significant difference.


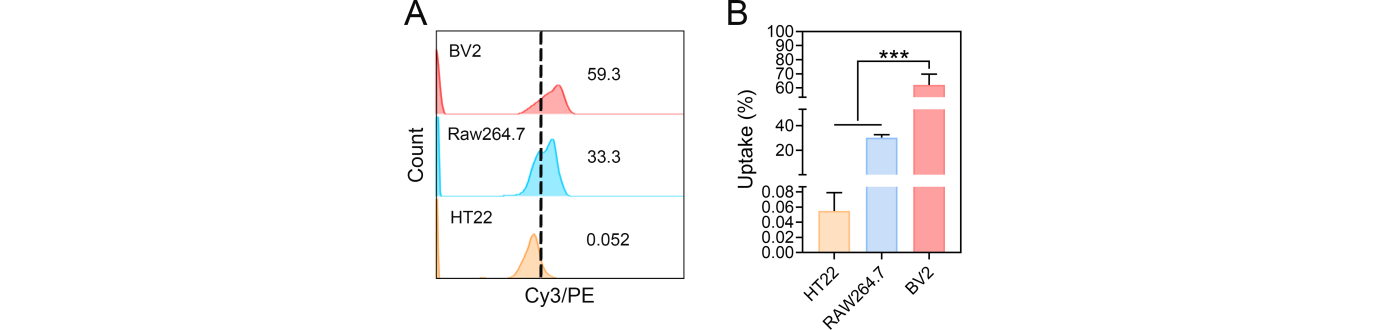


**Figure S7.** To analyze the selectivity of ME@FDsi uptake by different LPS-stimulated cell types in a co-culture system, BV2 (microglia), Raw264.7 (peripheral macrophages), and HT22 (neurons) cells were co-cultured and pretreated with 1 µg/mL LPS for 24 h to simulate an inflammatory environment, followed by incubation with Cy3-labeled ME@FDsi for 4 h. Microglia (CD11b⁺ TMEM119⁺) and peripheral macrophages (CD11b⁺ TMEM119⁻) were distinguished by double staining with CD11b and TMEM119, while neurons (NeuN⁺) were identified by NeuN staining. Uptake of ME@FDsi by each cell population was detected by flow cytometry. Data are presented as mean ± standard deviation. Statistical analysis was performed using one-way ANOVA. **p* < 0.05, ***p* < 0.01, ****p* < 0.001, *n.s.* indicates no significant difference.


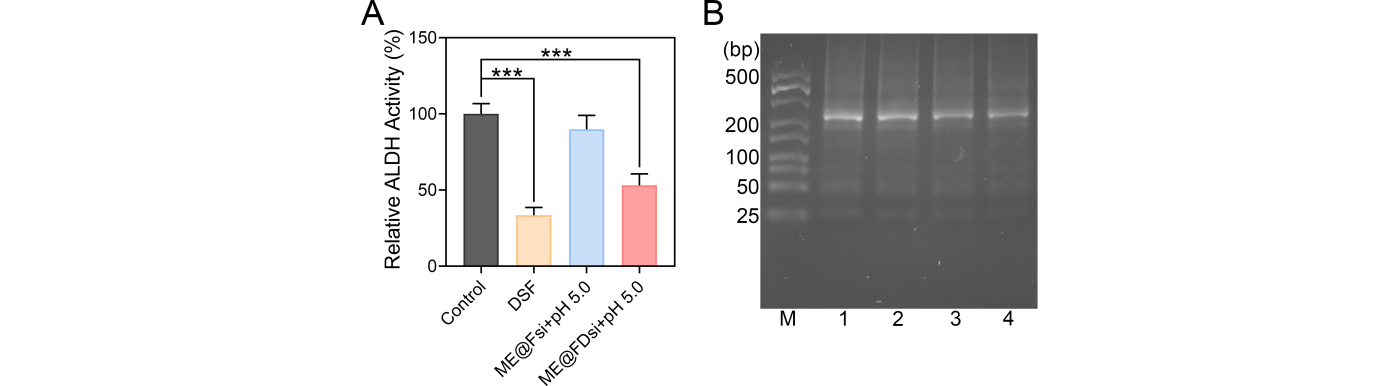


**Figure S8. (A)** Functional activity assessment of DSF in ME@FDsi after 8 h of incubation in a simulated lysosomal environment. Pure DSF (not incubated in lysosomal environment), ME@Fsi, and ME@FDsi were incubated in pH 5.0 buffer for 8 h, followed by detection of their inhibitory effects on relative ALDH enzyme activity. The Control group was untreated and set as 100%. **(B)** Structural stability analysis of tFNA in ME@FDsi under simulated lysosomal conditions. ME@FDsi was incubated in pH 5.0 buffer for 1, 2, 4, and 8 h, and equal amounts of samples were subjected to agarose gel electrophoresis to assess the integrity of Fsi bands. M: Marker; Lane 1, 1h; Lane 2, 2h; Lane 3, 4h; Lane 4, 8h.Data are presented as mean ± standard deviation. Statistical analysis was performed using one-way ANOVA. **p* < 0.05, ***p* < 0.01, ****p* < 0.001, *n.s.* indicates no significant difference.


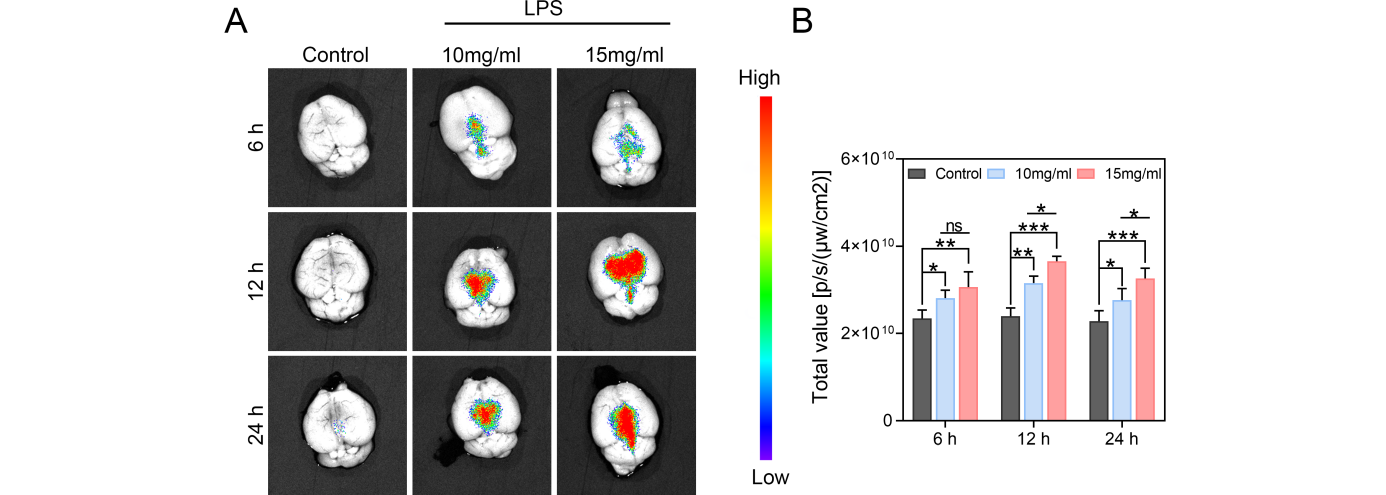


**Figure S9.** Inflammation-dependent analysis of ME@FDsi accumulation in the brain. (A) Healthy control mice (no LPS), 10 mg/kg LPS-treated mice, and 15 mg/kg LPS-treated mice were intravenously injected with DiD-labeled ME@FDsi via the tail vein. Brain tissues were collected at 6, 12, and 24 h post-injection for fluorescence imaging. (B) Quantitative analysis of fluorescence intensity in the brain injury region at each time point. Data are presented as mean ± standard deviation. Statistical analysis was performed using one-way ANOVA. **p* < 0.05, ***p* < 0.01, ****p* < 0.001, *n.s.* indicates no significant difference.


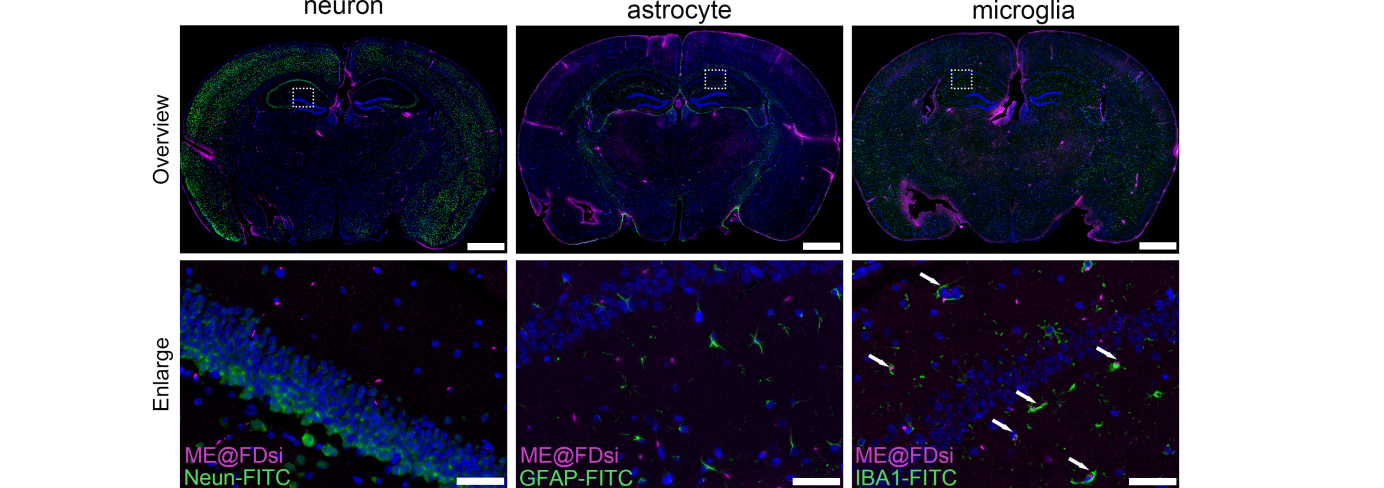


**Figure S10.** Cellular colocalization analysis of DiD-labeled ME@FDsi in the brain. SAE mice were intravenously injected with DiD-labeled ME@FDsi, and brain tissue sections were collected for immunofluorescence staining. Microglia were labeled with Iba1 antibody, neurons with NeuN antibody, and astrocytes with GFAP antibody. Nuclei were counterstained with DAPI. Scale bar: 1000 μm (overview) and 50 μm (magnified view).


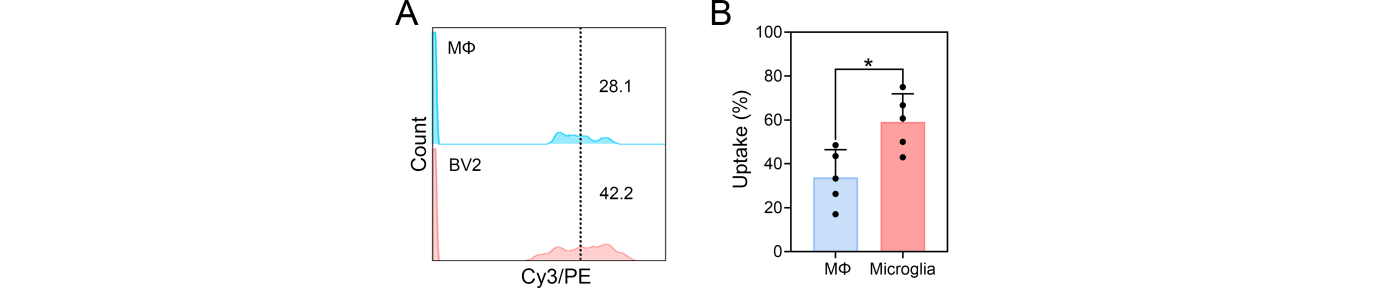


**Figure S11.** Flow cytometric analysis of ME@FDsi uptake by brain myeloid populations. Brain cells from SAE mice injected with Cy3-labeled ME@FDsi were analyzed by flow cytometry. (A) Representative flow cytometry plots showing ME@FDsi uptake by different myeloid populations. (B) Quantitative analysis of Cy3-positive cells (%) in resident microglia (CD11b⁺ CD45^(low)) and infiltrating macrophages (CD11b⁺ CD45^(high)). Data are presented as mean ± standard deviation. Statistical analysis was performed using one-way ANOVA. **p* < 0.05, ***p* < 0.01, ****p* < 0.001, n.s. indicates no significant difference.


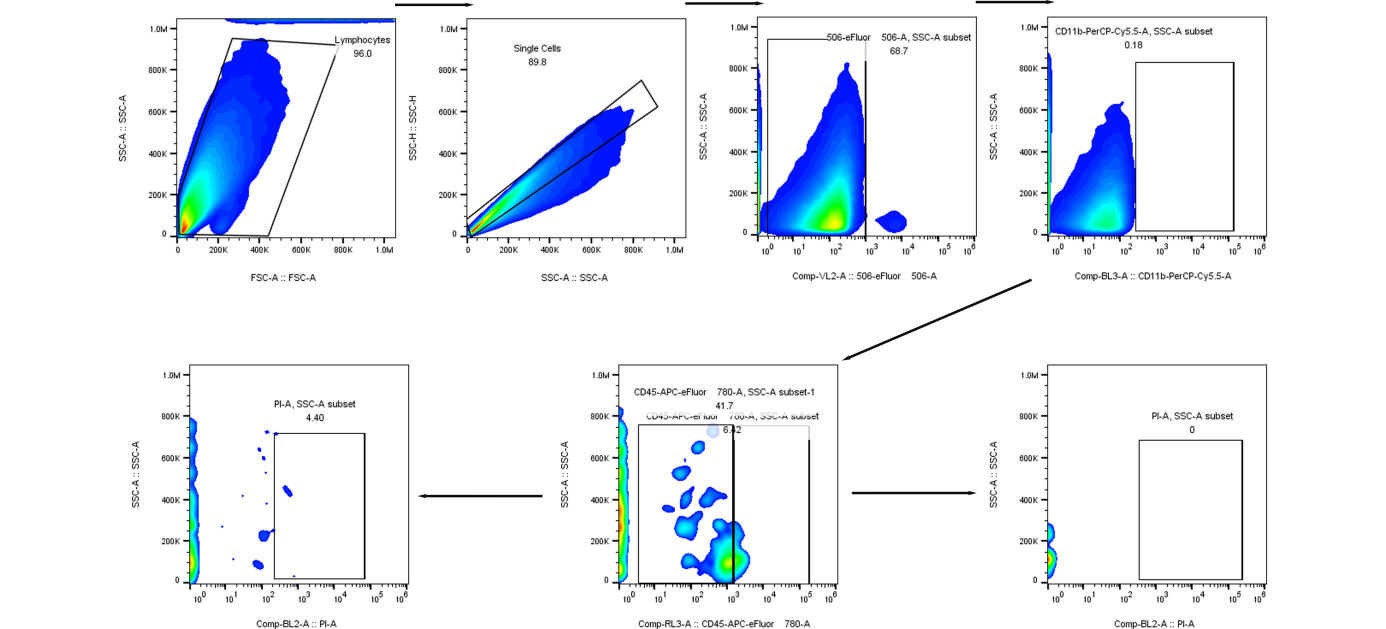


**Figure S12.** Representative gating strategy for distinguishing resident microglia (CD11b⁺ CD45^(low)) and infiltrating macrophages (CD11b⁺ CD45^(high)).


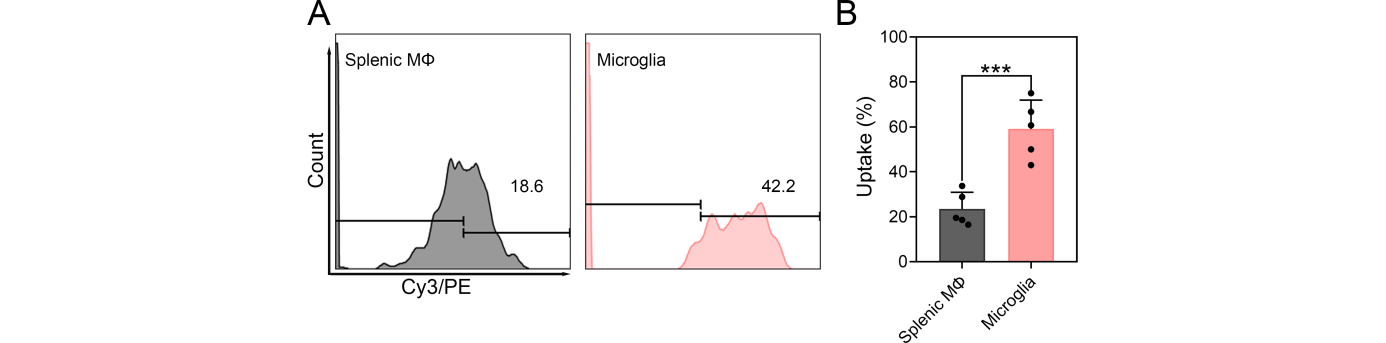


**Figure S13.** in the manuscript. Flow cytometric analysis of ME@FDsi distribution in peripheral and brain myeloid populations. SAE mice (10 mg/kg LPS) were intravenously injected with Cy3-labeled ME@FDsi via the tail vein. Spleen and brain tissues were collected at 12 h post-injection for flow cytometry analysis. (**A**) Representative flow cytometry plots showing ME@FDsi uptake by different myeloid populations. (**B**) Quantitative analysis of Cy3-positive cells (%) in resident microglia (CD11b⁺ CD45^(low)) and splenic macrophages (CD11b⁺ F4/80^(high)). Data are presented as mean ± standard deviation. Statistical analysis was performed using one-way ANOVA. **p* < 0.05, ***p* < 0.01, ****p* < 0.001, *n.s.* indicates no significant difference.


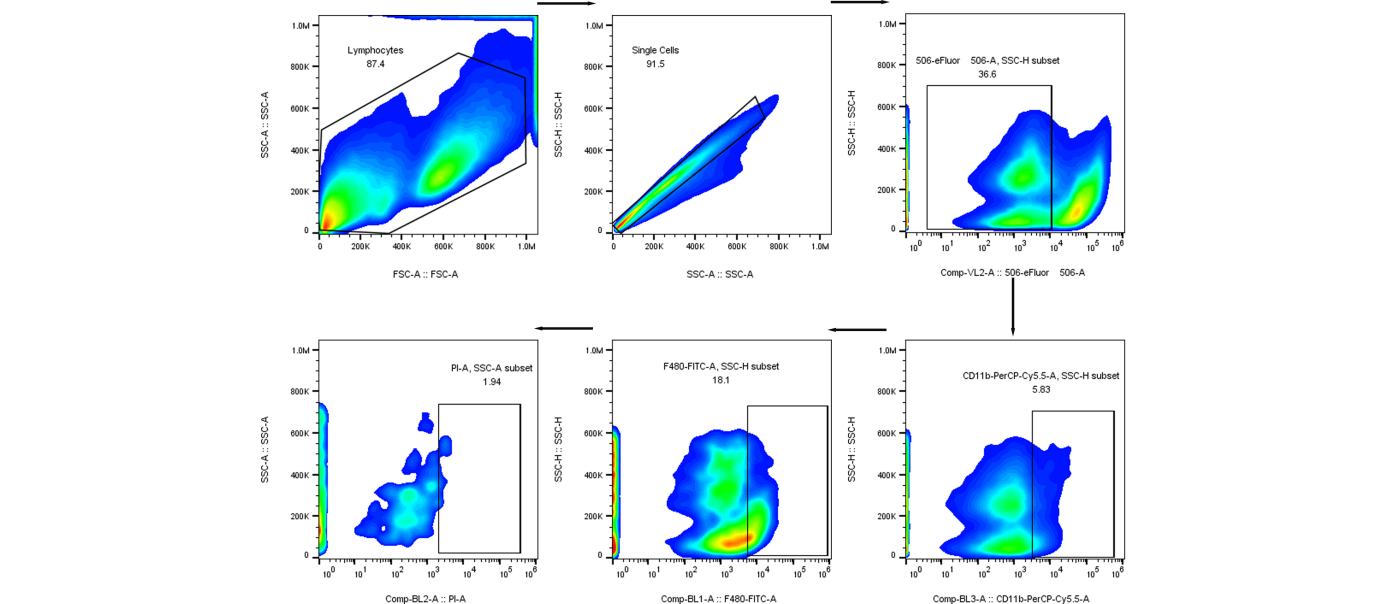


**Figure S14.** Representative gating strategy for splenic macrophages (CD11b⁺ F4/80^(high)).


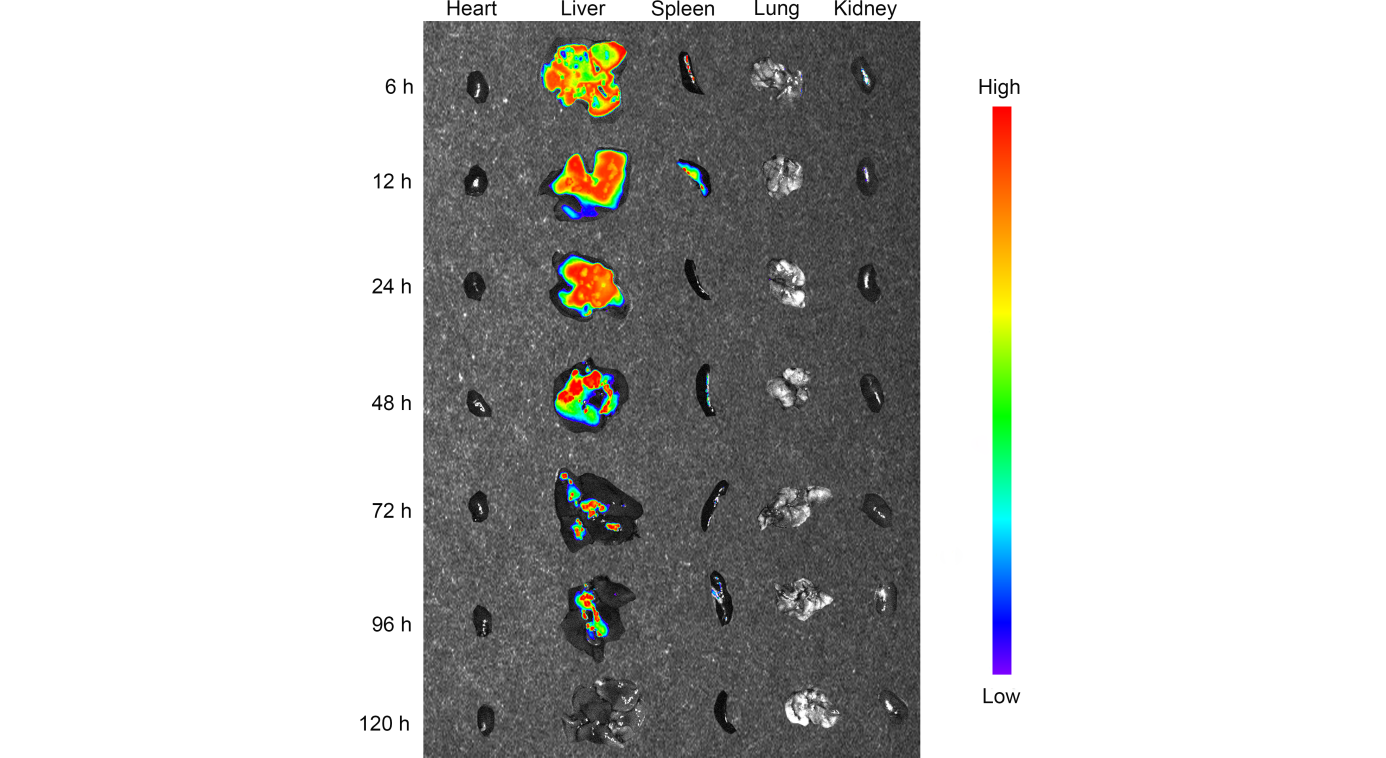


**Figure S15.**  Time-dependent distribution of ME@FDsi in major organs of SAE mice. SAE mice were intravenously injected with DID-labeled ME@FDsi via the tail vein. Heart, liver, spleen, lungs, and kidneys were collected at 6, 12, 24, 48, 72, 96, and 120 h post-injection for ex vivo fluorescence imaging. Representative fluorescence images of each organ at each time point are shown, with the color scale indicating fluorescence intensity.


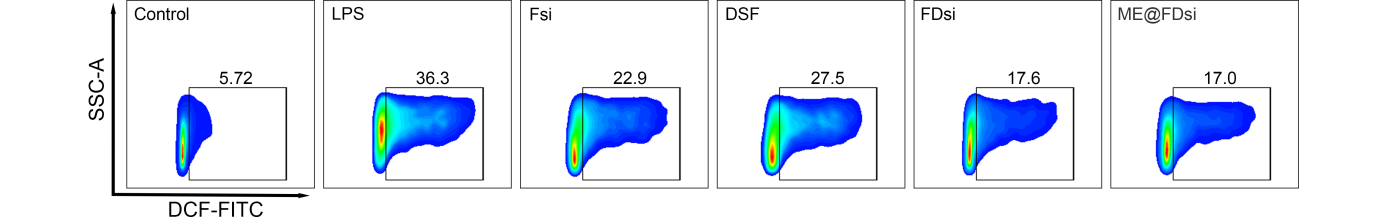


**Figure S16.** Intracellular reactive oxygen species (ROS) generation in BV2 cells under different treatment conditions was detected by flow cytometry.


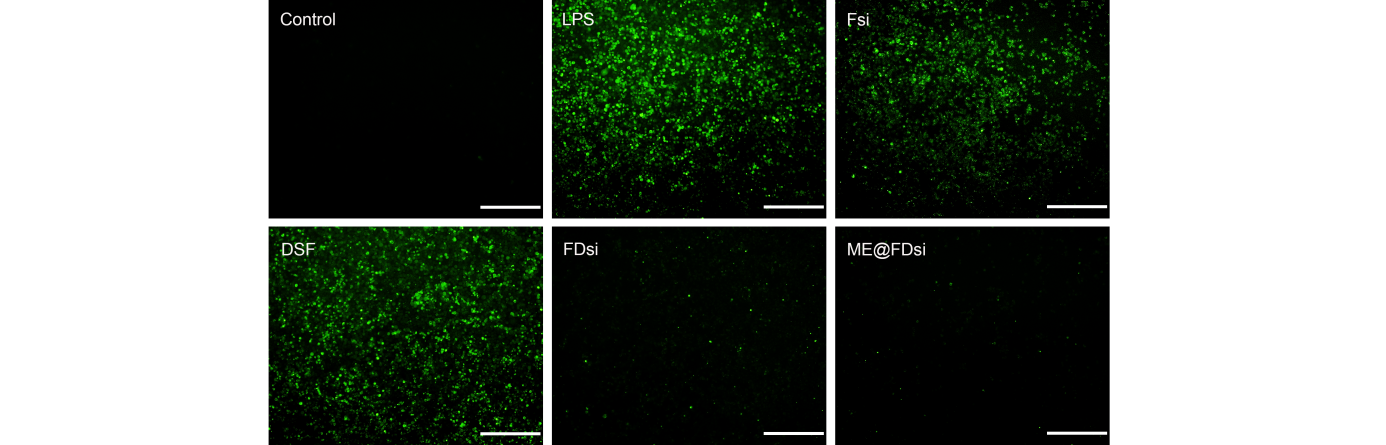


**Figure S17.** Fluorescence imaging of ROS in BV2 cells under different treatments (green: ROS labeled by DCFH-DA). Scale bar, 200 μm.


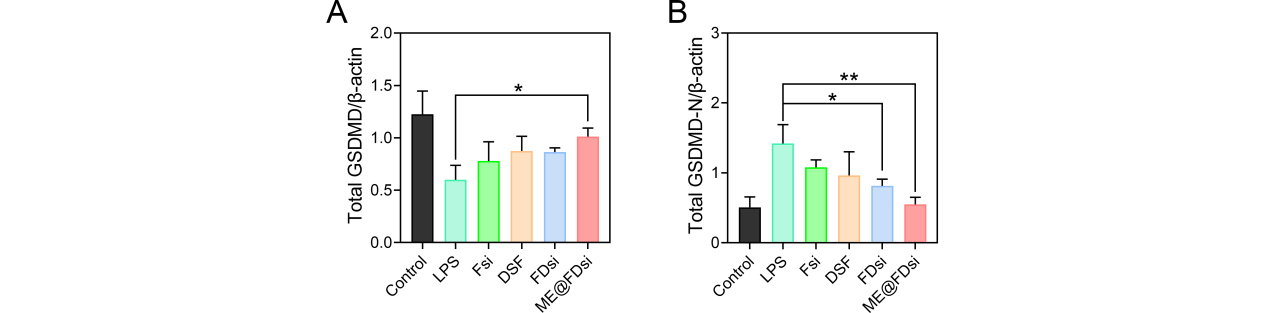


**Figure S18.** Quantitative results of the expression levels of full-length GSDMD and cleaved GSDMD-N relative to β-actin in injured cells after various treatments. Data are presented as mean ± standard deviation. Statistical analysis was performed using one-way ANOVA. **p* < 0.05, ***p* < 0.01, ****p* < 0.001, *n.s.* indicates no significant difference.


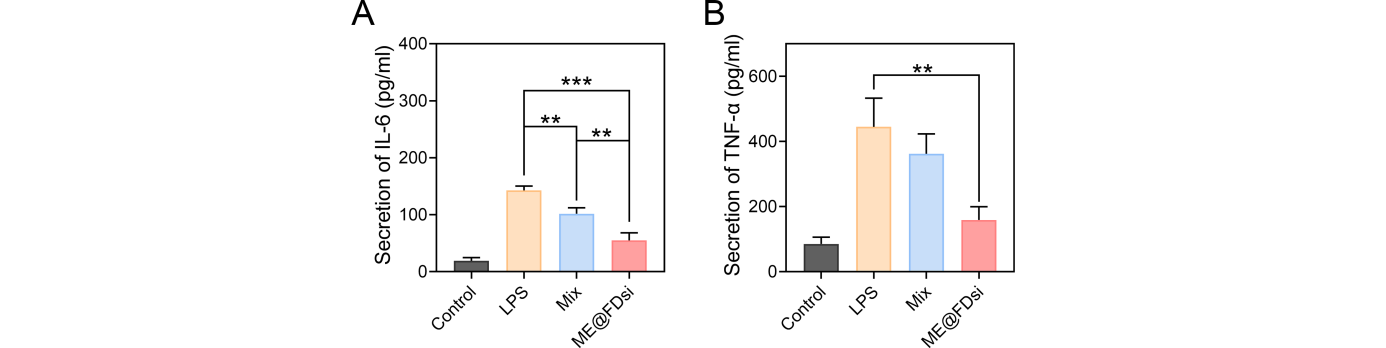


**Figure S19.** Secretion of inflammatory cytokines, including IL-6 and TNF-α, from BV2 cells after LPS stimulation was detected by ELISA. **Data are presented as mean ± standard deviation. Statistical analysis was performed using one-way ANOVA. **p* < 0.05, ***p* < 0.01, ****p* < 0.001, *n.s.* indicates no significant difference.**


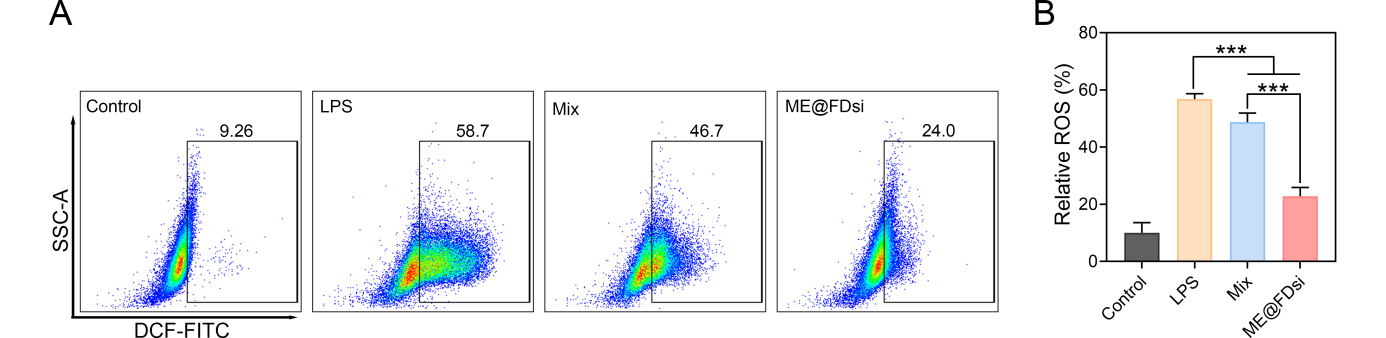


**Figure S20.** Intracellular reactive oxygen species (ROS) generation in BV2 cells under different treatment conditions was detected by flow cytometry. **Data are presented as mean ± standard deviation. Statistical analysis was performed using one-way ANOVA. **p* < 0.05, ***p* < 0.01, ****p* < 0.001, *n.s.* indicates no significant difference.**


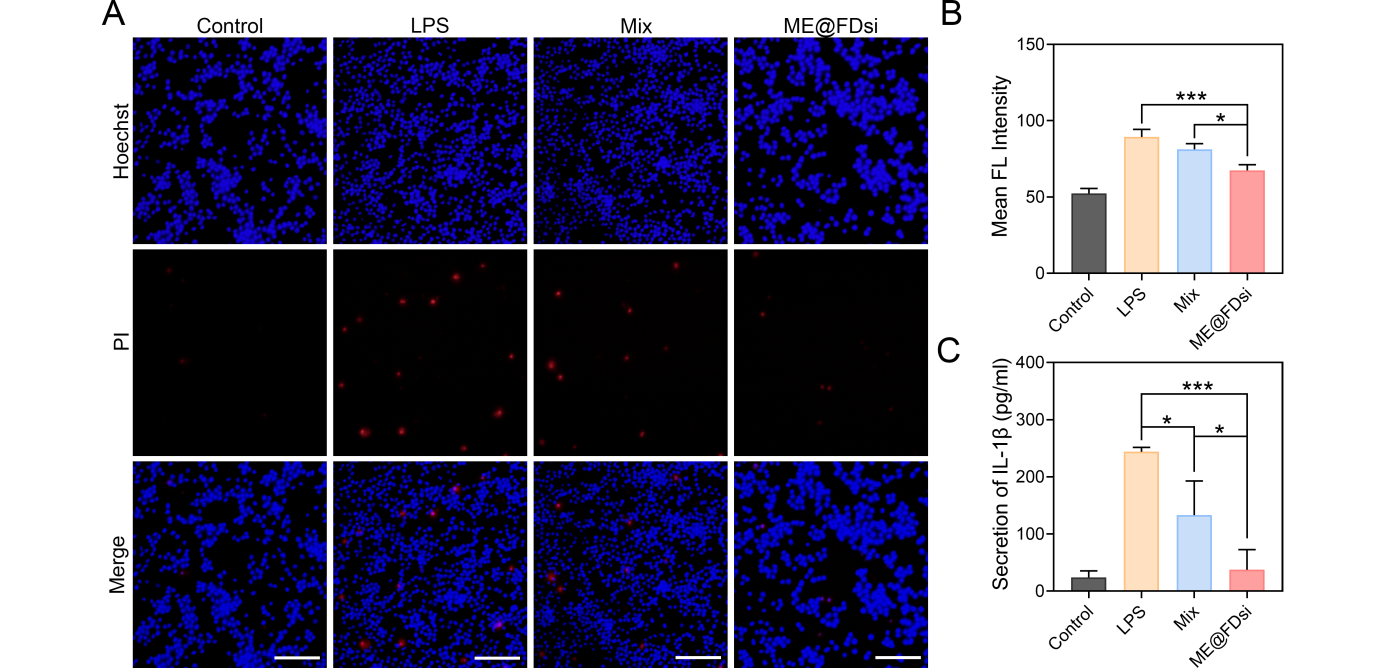


**Figure S21. (A-B)** BV2 cell status after different treatments was detected using Hoechst 33342 / Propidium Iodide (PI) double staining. Scale bar is 200 µm. **(C)** Secretion of the inflammatory cytokine IL-1β from BV2 cells after LPS stimulation was detected by ELISA. **Data are presented as mean ± standard deviation. Statistical analysis was performed using one-way ANOVA. **p* < 0.05, ***p* < 0.01, ****p* < 0.001, *n.s.* indicates no significant difference.**


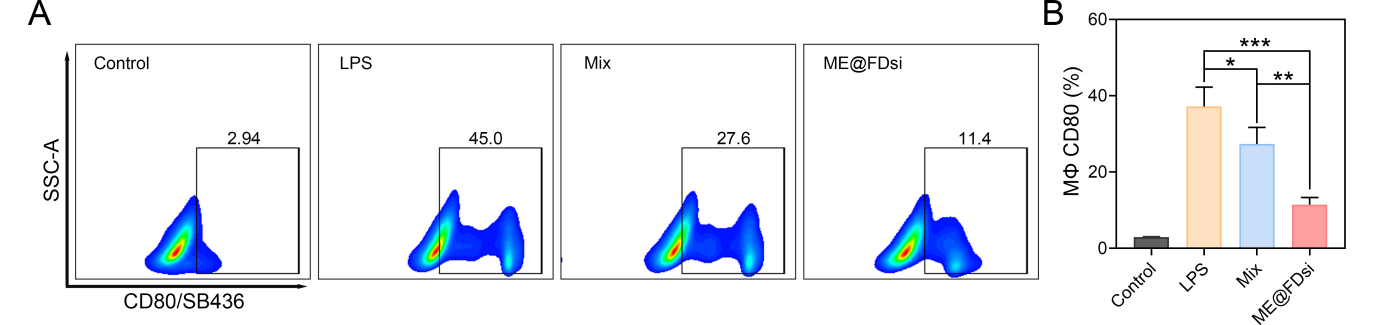


**Figure S22. (A, B)** Expression of the M1-type cell marker CD80 in BV2 cells under different treatment conditions was detected by flow cytometry, with (**B**) showing the quantitative analysis of CD80 levels. **Data are presented as mean ± standard deviation. Statistical analysis was performed using one-way ANOVA. **p* < 0.05, ***p* < 0.01, ****p* < 0.001, *n.s.* indicates no significant difference.**


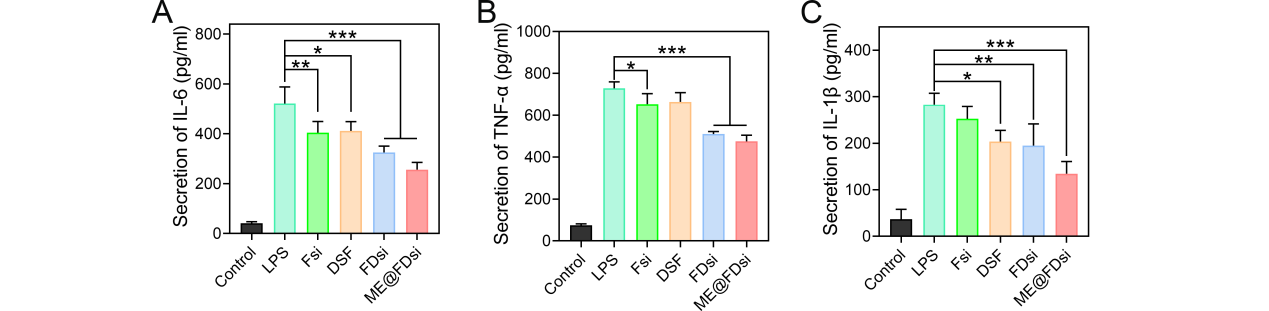


**Figure S23.** Secretion of inflammatory cytokines including IL-6, TNF-α and IL-1β from Raw 264.7 cells after LPS stimulation was detected by ELISA. Data are presented as mean ± standard deviation. Statistical analysis was performed using one-way ANOVA. **p* < 0.05, ***p* < 0.01, ****p* < 0.001, *n.s.* indicates no significant difference.

**
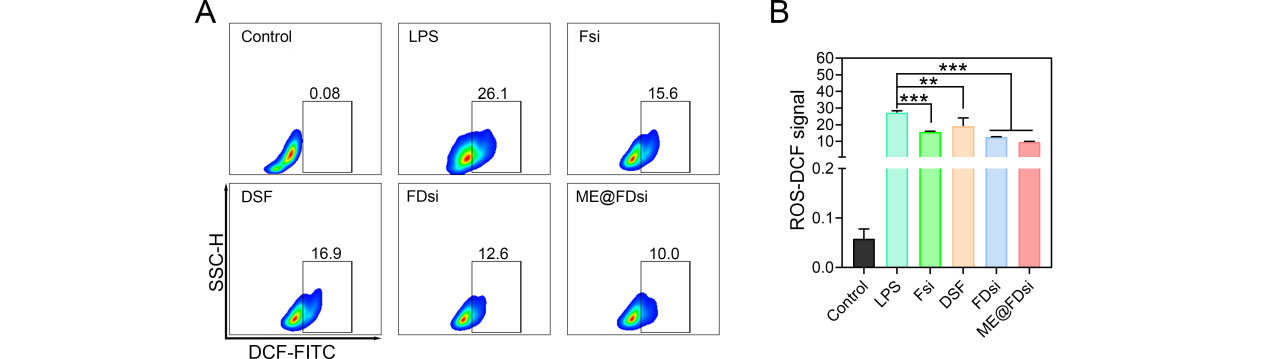
**

**Figure S24.** Intracellular ROS production in Raw 264.7 cells after various treatments, measured by flow cytometry. Data are presented as mean ± standard deviation. Statistical analysis was performed using one-way ANOVA. **p* < 0.05, ***p* < 0.01, ****p* < 0.001, *n.s.* indicates no significant difference.

**
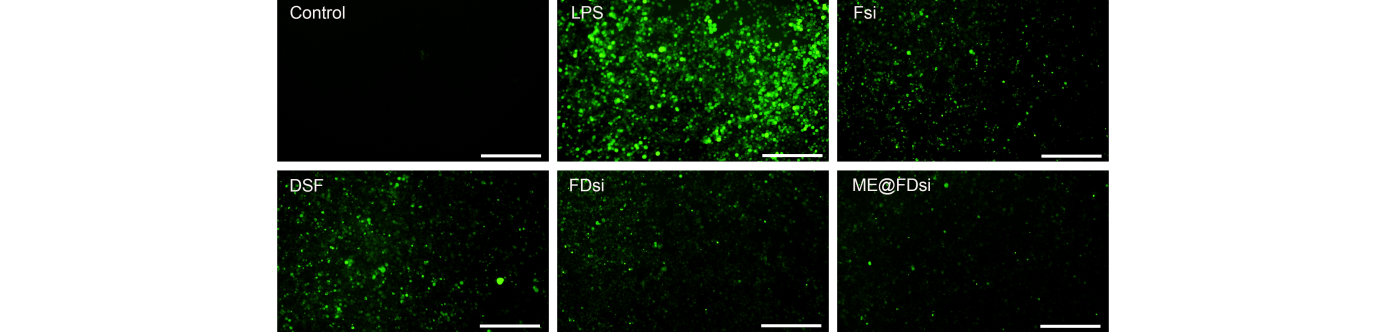
**

**Figure S25.** Fluorescence imaging of ROS in Raw 264.7 cells under different treatments (green: ROS labeled with DCFH-DA). Scale bar, 200 μm.


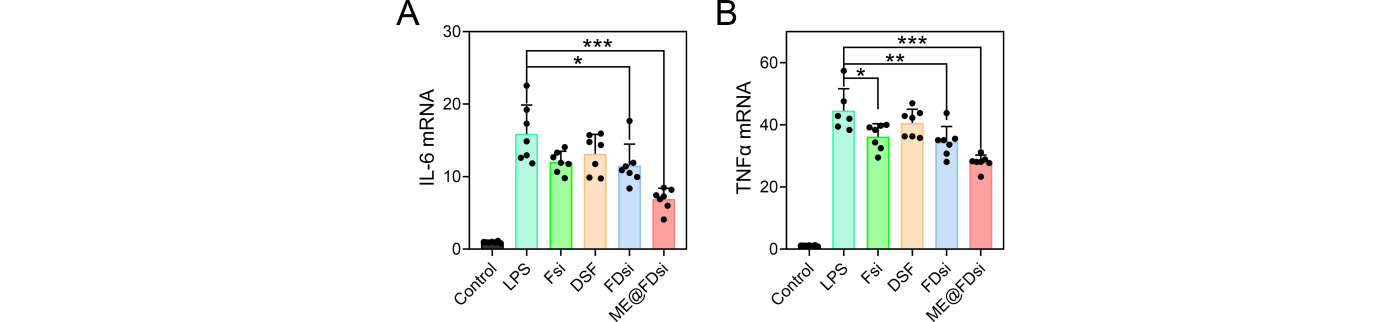


**Figure S26. (A, B)** Expression of *IL-6* and *TNF-α* in the brain tissue of septic mice was analyzed by RT-PCR. **Data are presented as mean ± standard deviation. Statistical analysis was performed using one-way ANOVA. **p* < 0.05, ***p* < 0.01, ****p* < 0.001, *n.s.* indicates no significant difference.**


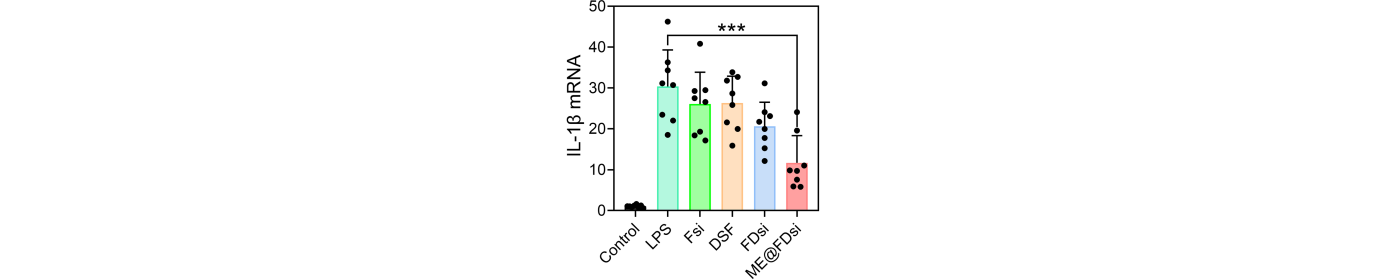


**Figure S27.** Expression of *IL-1β* in the brain tissue of septic mice was analyzed by RT-PCR. **Data are presented as mean ± standard deviation. Statistical analysis was performed using one-way ANOVA. **p* < 0.05, ***p* < 0.01, ****p* < 0.001, *n.s.* indicates no significant difference.**

**
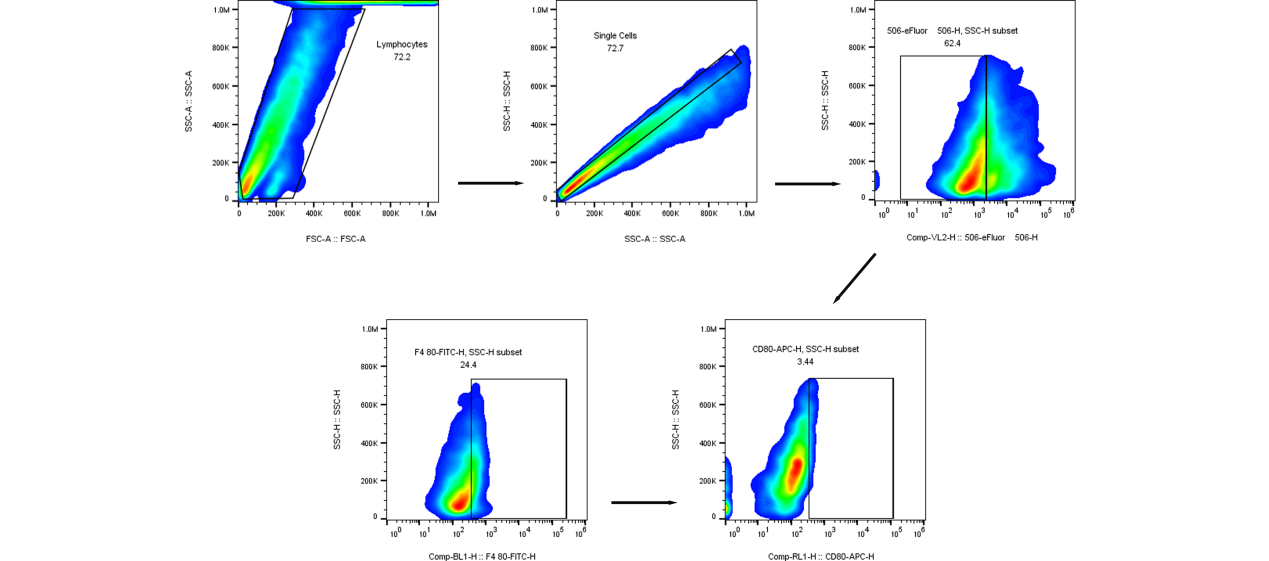
**

**Figure S28.** Gating strategy for sorting M1 macrophages from brain tissue.


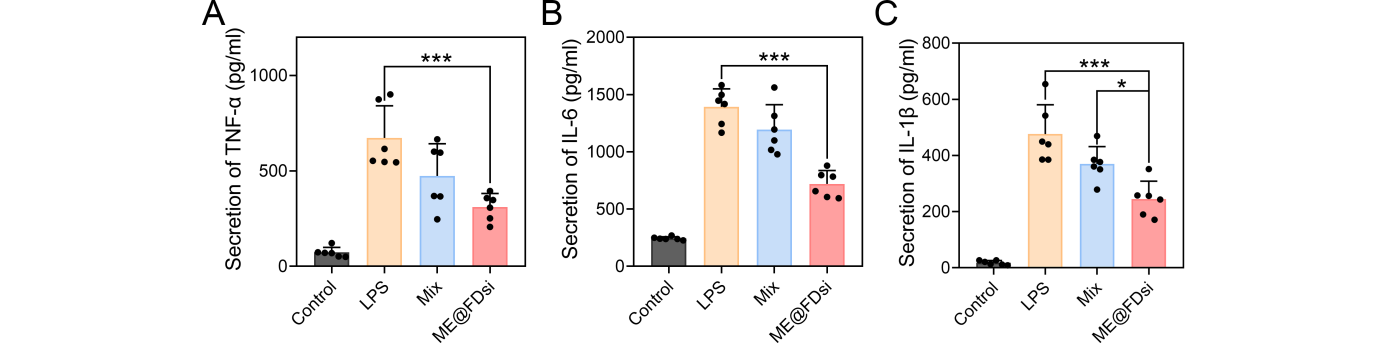


**Figure S29.** **(A-C)** Secretion of inflammatory cytokines, including IL-6, TNF-α, and IL-1β, in the brain tissue of septic mice was detected by ELISA. **Data are presented as mean ± standard deviation. Statistical analysis was performed using one-way ANOVA. **p* < 0.05, ***p* < 0.01, ****p* < 0.001, *n.s.* indicates no significant difference.**


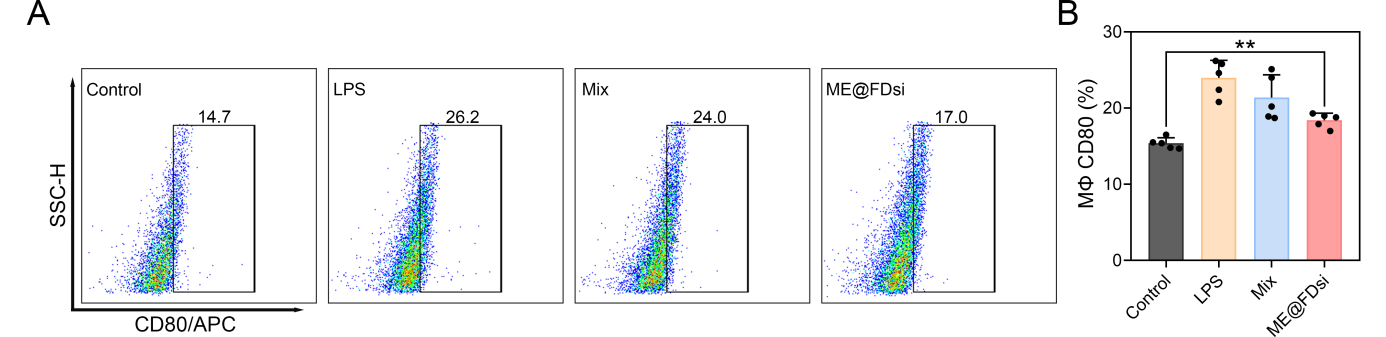


**Figure S30.** **(A, B)** Flow cytometric analysis of the CD80+ immune cell population in mouse brain tissue and quantitative analysis of CD80 levels. **Data are presented as mean ± standard deviation. Statistical analysis was performed using one-way ANOVA. **p* < 0.05, ***p* < 0.01, ****p* < 0.001, *n.s.* indicates no significant difference.**


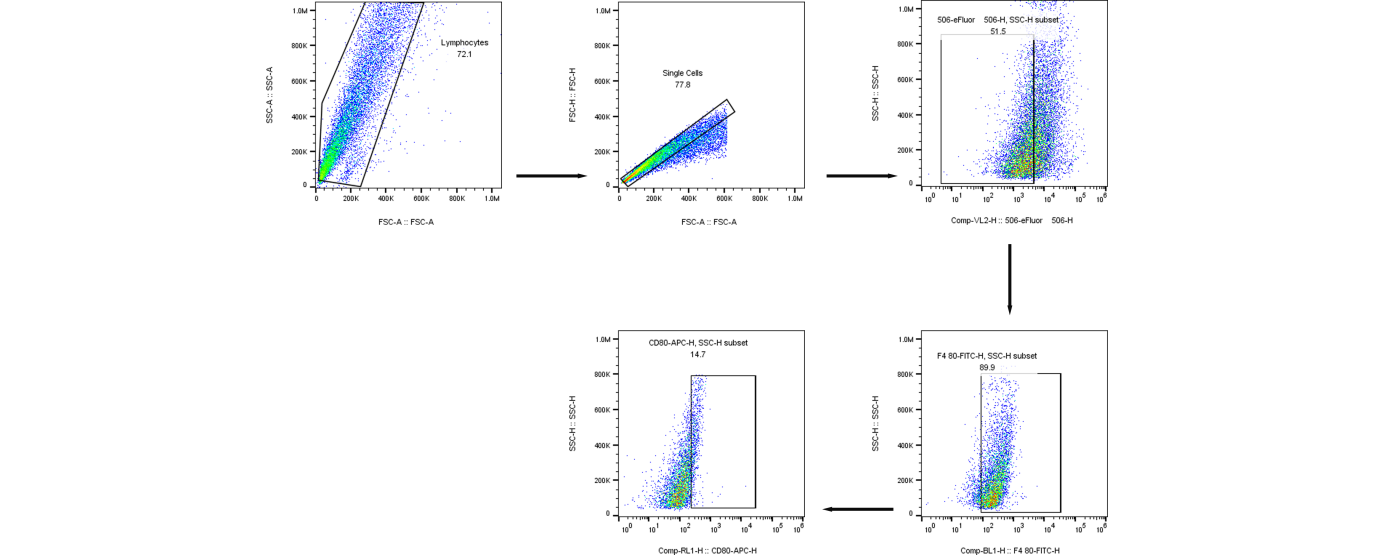


**Figure S31.** Gating strategy for sorting M1 macrophages from brain tissue.


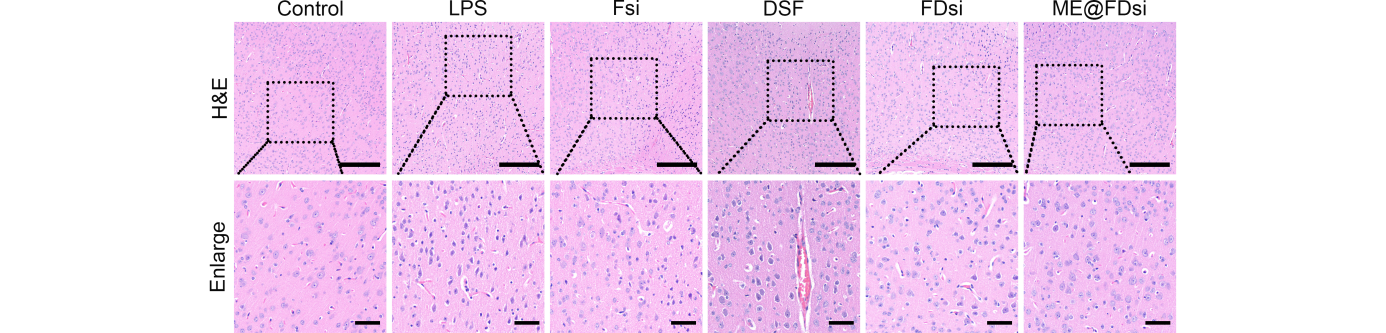


**Figure S32.** Representative images of H&E staining of brain tissue in the cortex on day 3 post-modeling (scale bar: 200 µm) and magnified views (scale bar: 50 µm).


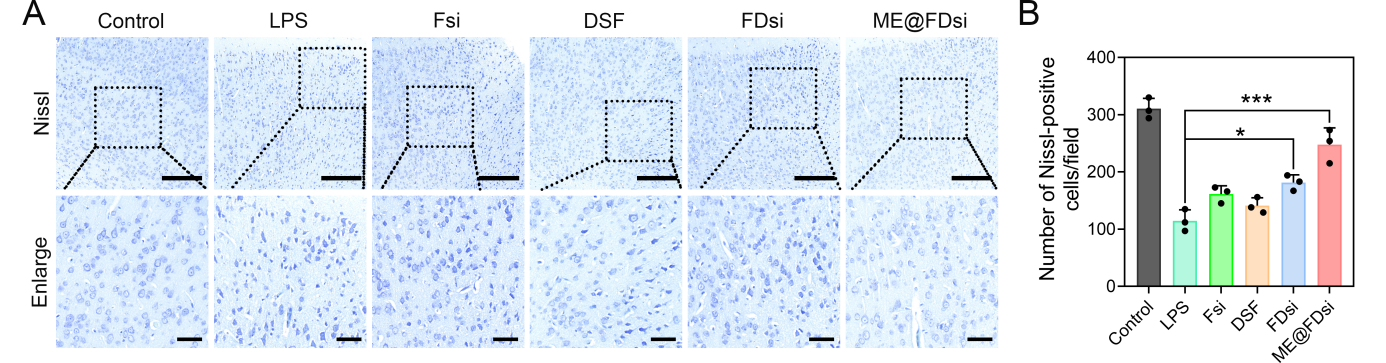


**Figure S33. (A)** Representative images of Nissl-positive cells in the cortex on day 3 post-modeling (scale bar: 200 µm) and magnified views (scale bar: 50 µm). **(B)** Quantitative analysis of Nissl-positive cells. **Data are presented as mean ± standard deviation. Statistical analysis was performed using one-way ANOVA. **p* < 0.05, ***p* < 0.01, ****p* < 0.001, *n.s.* indicates no significant difference.**


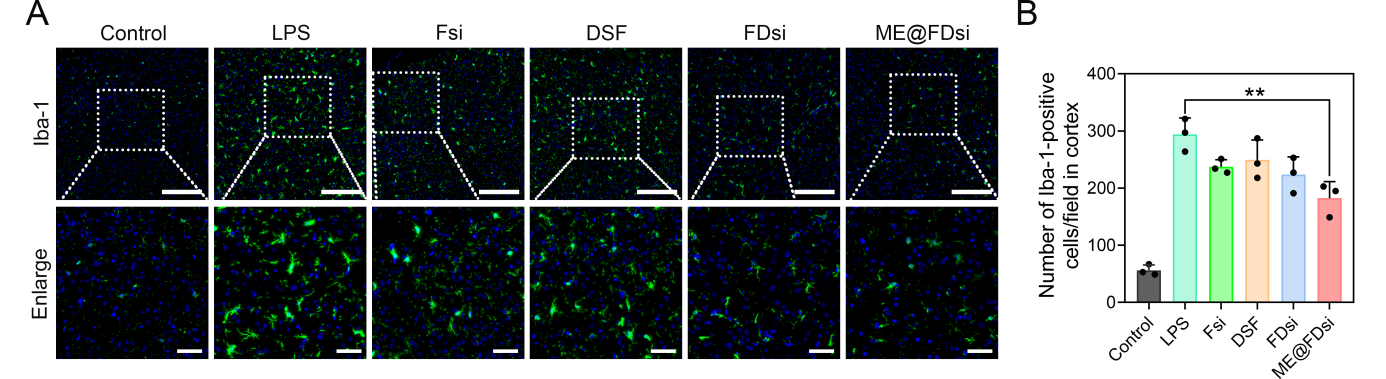


**Figure S34. (A)** Representative immunofluorescence images showing Iba-1 expression in the injured cortex region of septic model mice from different treatment groups on day 3 post-treatment (scale bar: 200 µm) and magnified views (scale bar: 50 µm). **(B)** Quantitative analysis of Iba-1-positive cells in the injured cortex region of septic model mice from different treatment groups on day 3 post-treatment. **Data are presented as mean ± standard deviation. Statistical analysis was performed using one-way ANOVA. **p* < 0.05, ***p* < 0.01, ****p* < 0.001, *n.s.* indicates no significant difference.**


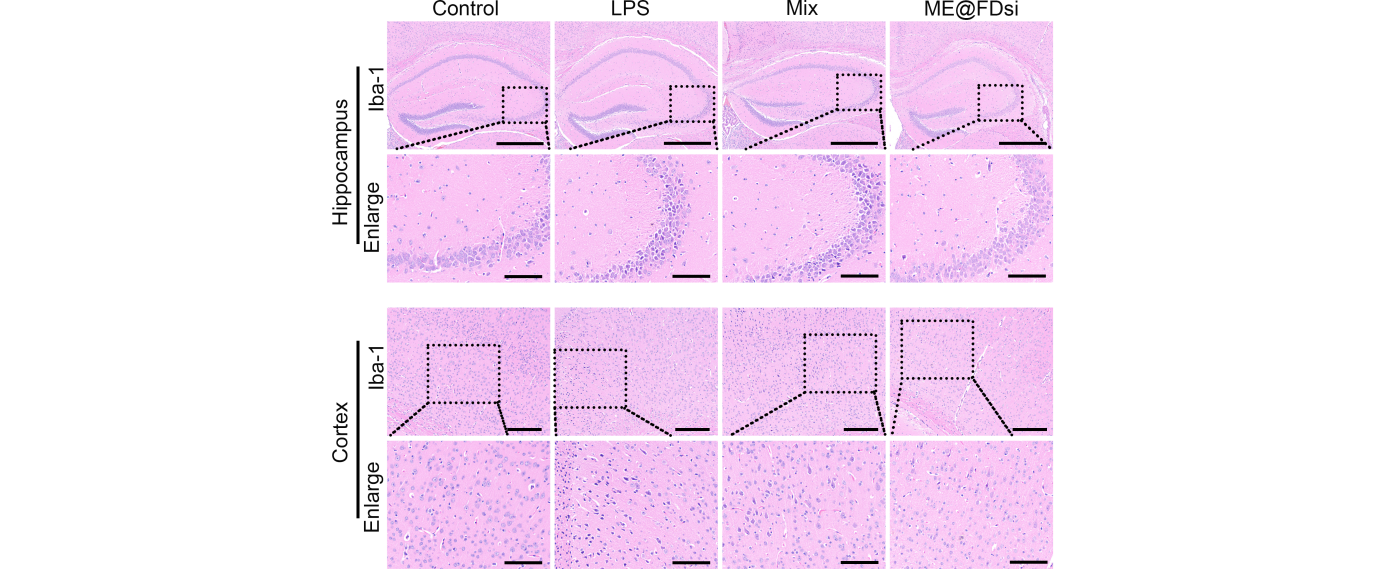


**Figure S35.** Representative images of H&E staining in the hippocampus and cortex on day 3 post-modeling (scale bar: 200 µm) and magnified views (scale bar: 50 µm).


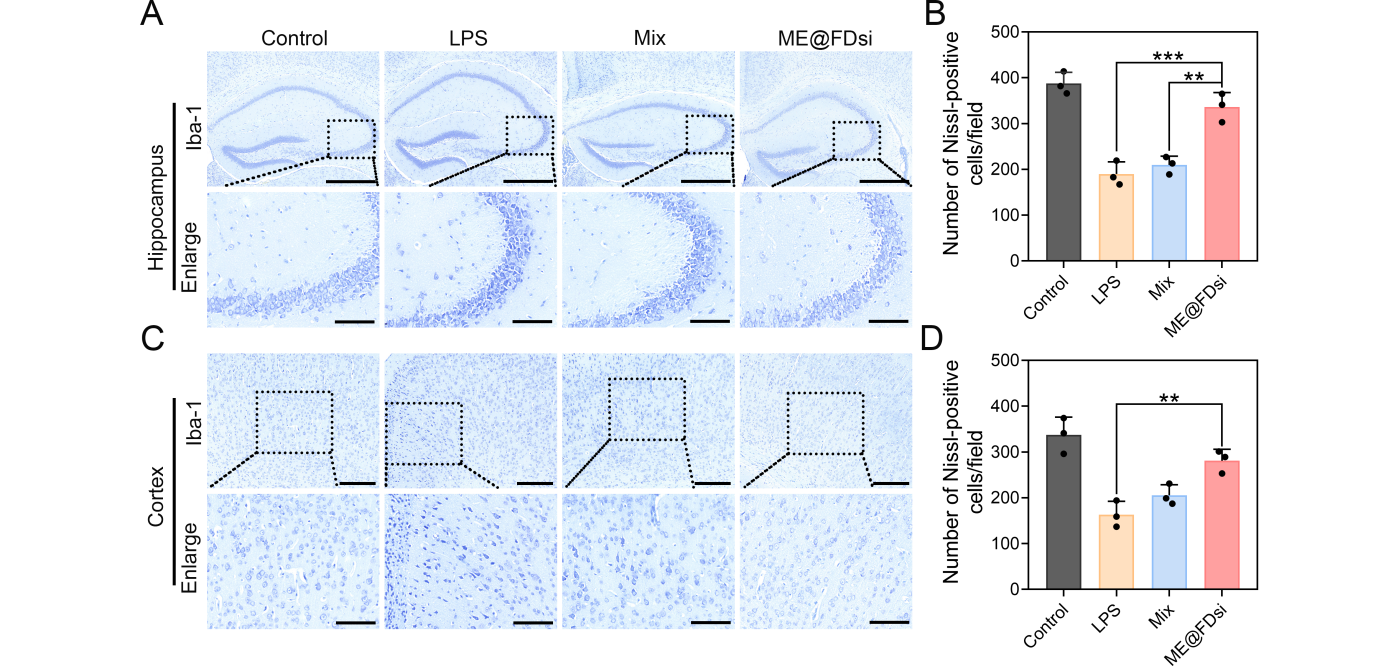


**Figure S36. (A)** Representative images of Nissl-positive cells in the hippocampus and cortex on day 3 post-modeling (scale bar: 200 µm) and magnified views (scale bar: 50 µm). **(B)** Quantitative analysis of Nissl-positive cells. **Data are presented as mean ± standard deviation. Statistical analysis was performed using one-way ANOVA. **p* < 0.05, ***p* < 0.01, ****p* < 0.001, *n.s.* indicates no significant difference.**


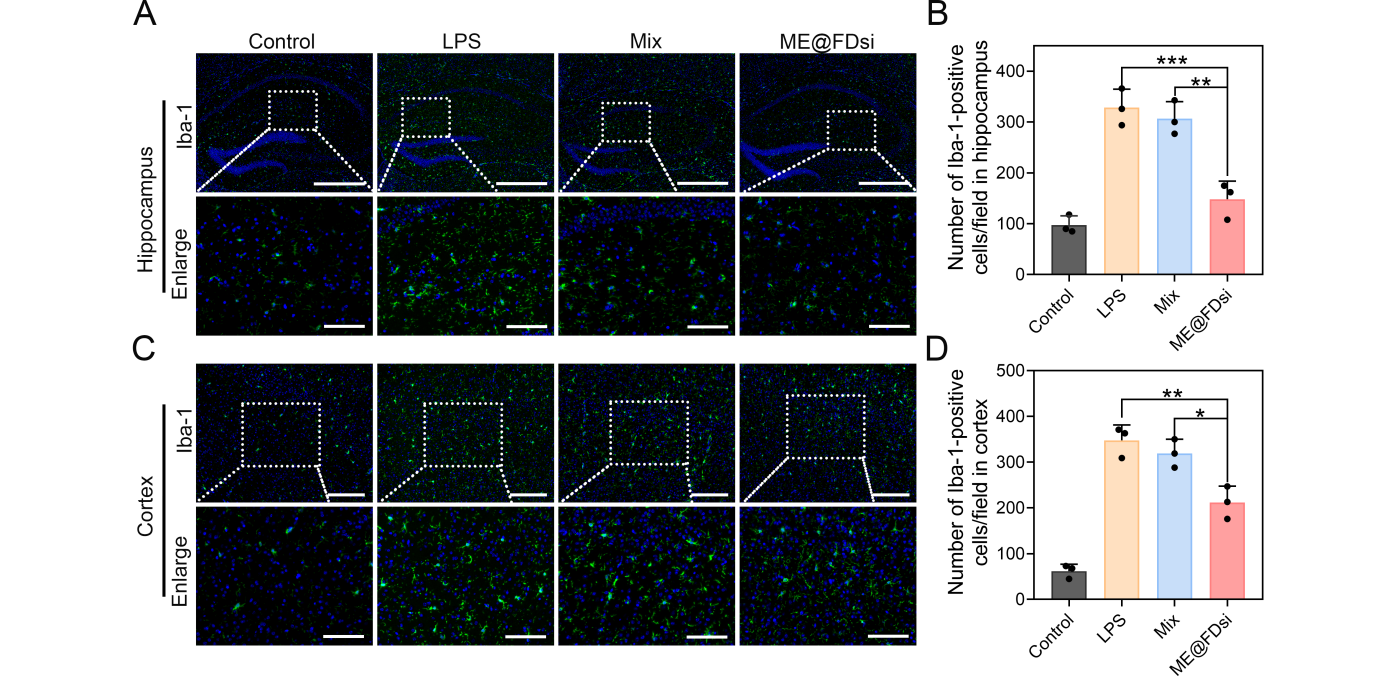


**Figure S37. (A)** Representative immunofluorescence images showing Iba-1 expression in the injured hippocampus and cortex region of septic model mice from different treatment groups on day 3 post-treatment (scale bar: 200 µm) and magnified views (scale bar: 50 µm). **(B)** Quantitative analysis of Iba-1-positive cells in the injured hippocampus and cortex region of septic model mice from different treatment groups on day 3 post-treatment. **Data are presented as mean ± standard deviation. Statistical analysis was performed using one-way ANOVA. **p* < 0.05, ***p* < 0.01, ****p* < 0.001, *n.s.* indicates no significant difference.**


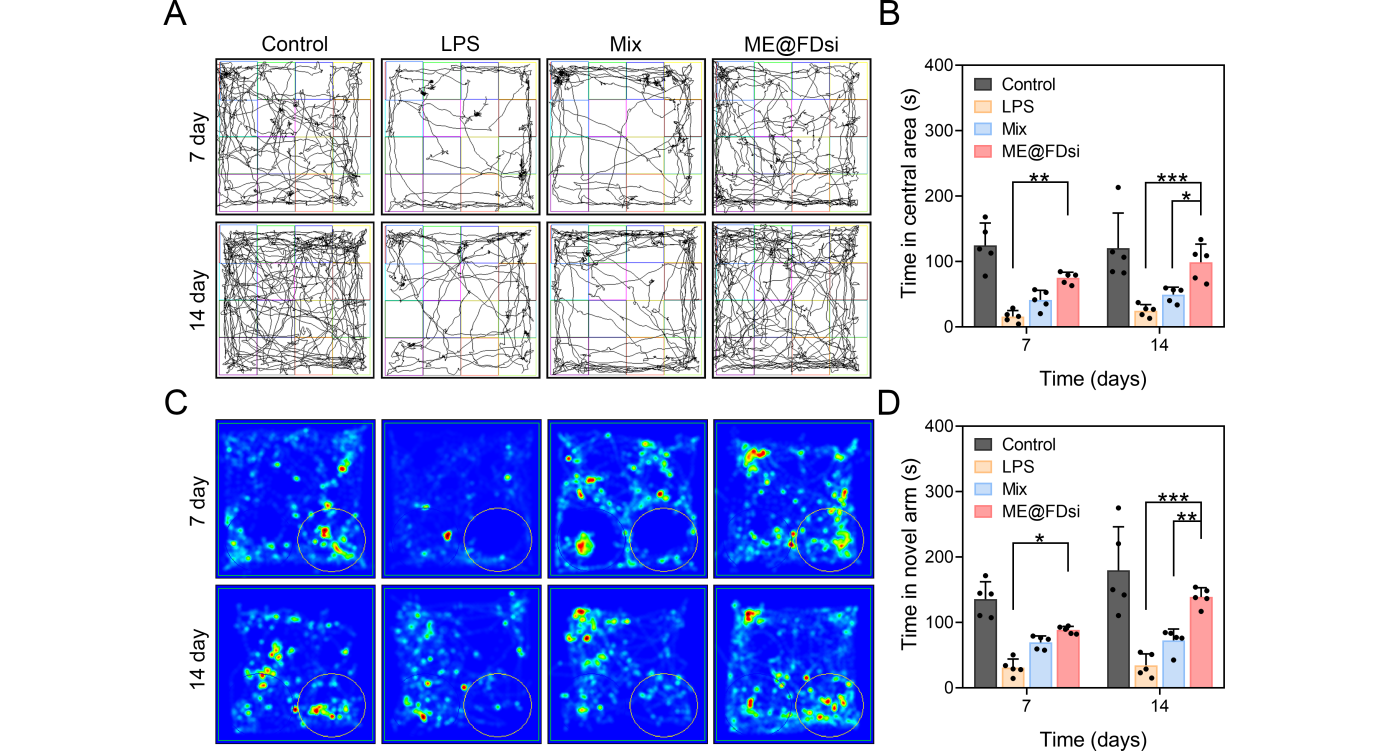


**Figure S38.** Validation of the durability of ME@FDsi therapeutic effects and the necessity of the nanostructure. SAE mice were treated with PBS (Control), LPS, the simple mixture (Mix), or fully assembled ME@FDsi. Behavioral tests were performed on days 7 and 14 post-treatment. (A) Center zone residence time in the open field test, reflecting anxiety-like behavior and exploratory drive. (B) Novel object exploration time in the novel object recognition test, reflecting recognition memory. Data are presented as mean ± standard deviation. Statistical analysis was performed using one-way ANOVA. **p* < 0.05, ***p* < 0.01, ****p* < 0.001, *n.s.* indicates no significant difference.


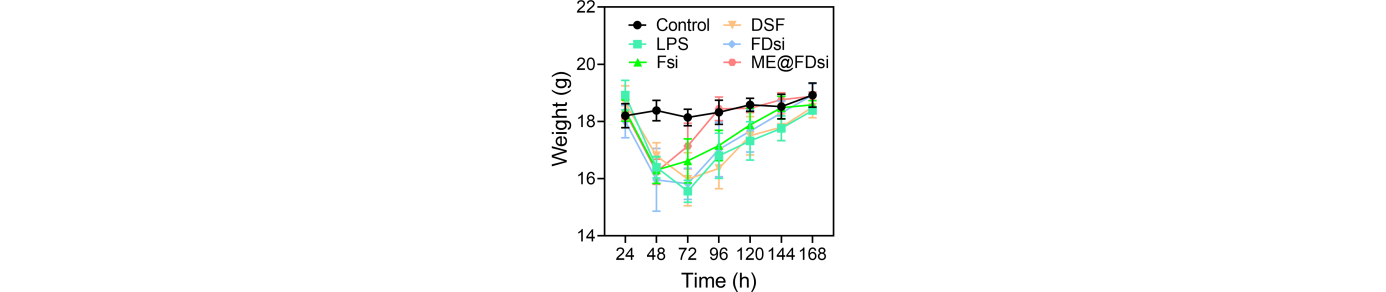


**Figure S39.** Body weight records of septic mice.


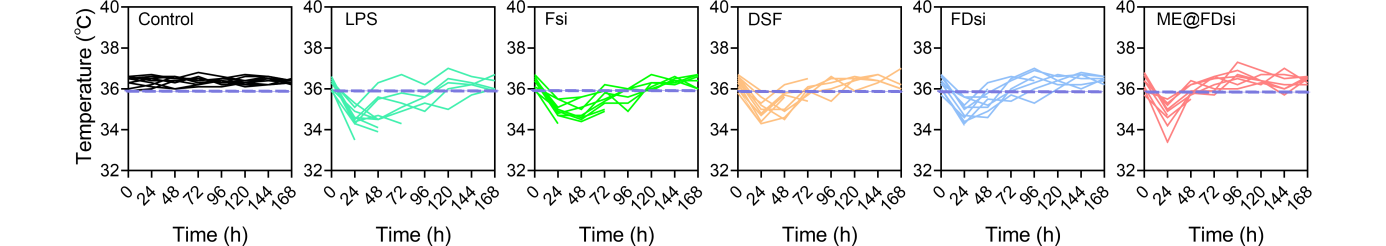


**Figure S40.** Body temperature records of septic mice.


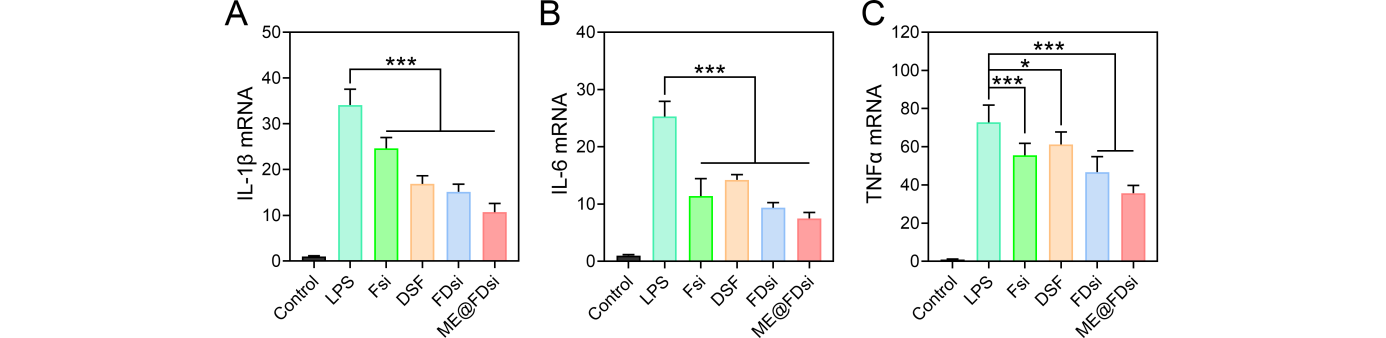


**Figure S41. (A-C)** Expression of *IL-1β*, *IL-6*, and *TNF-α* in the lung tissue of septic mice was analyzed by RT-PCR. **Data are presented as mean ± standard deviation. Statistical analysis was performed using one-way ANOVA. **p* < 0.05, ***p* < 0.01, ****p* < 0.001, *n.s.* indicates no significant difference.**

**
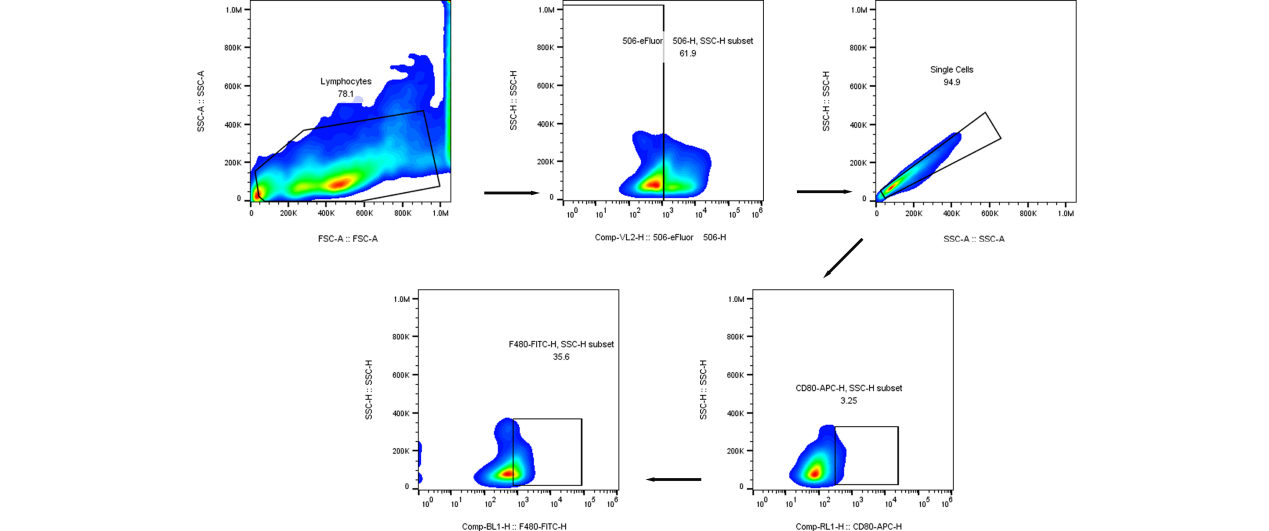
**

**Figure S42.** Gating strategy for sorting M1 macrophages from splenic tissue.

**
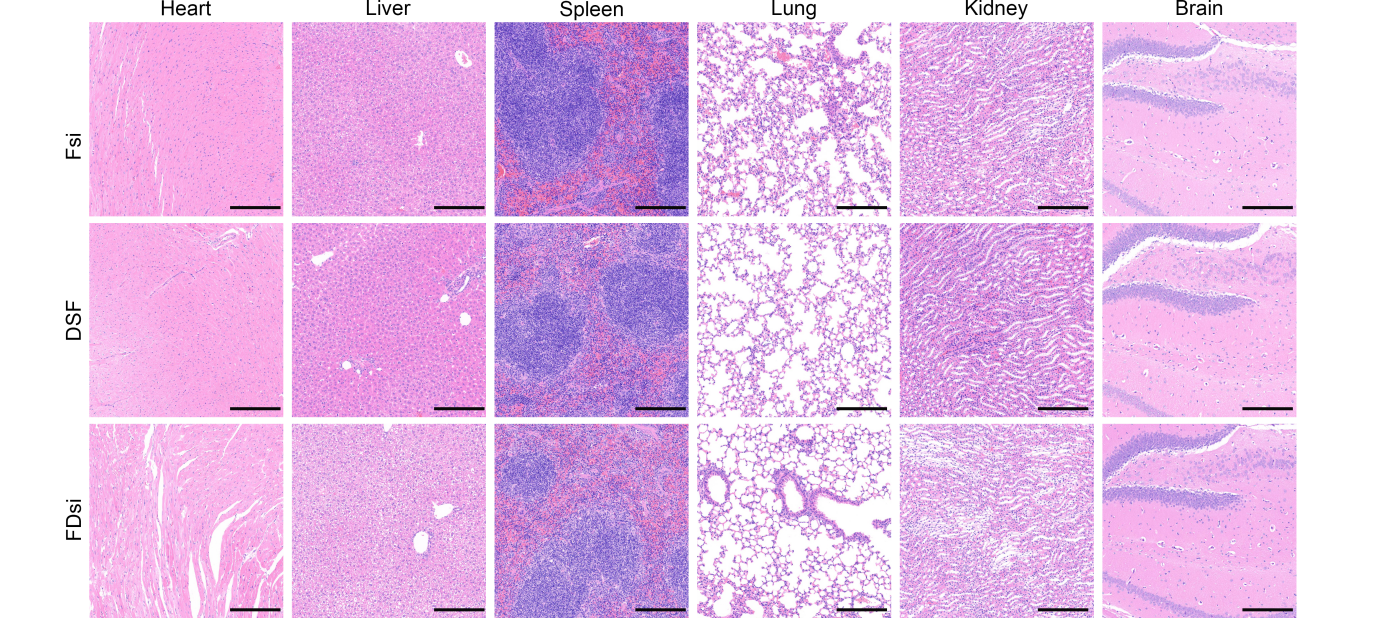
**

**Figure S43. H&E staining results of various organs from healthy mice after intravenous administration of Fsi, DSF, and FDsi *via* the tail vein. Scale bar = 200 μm.**

**
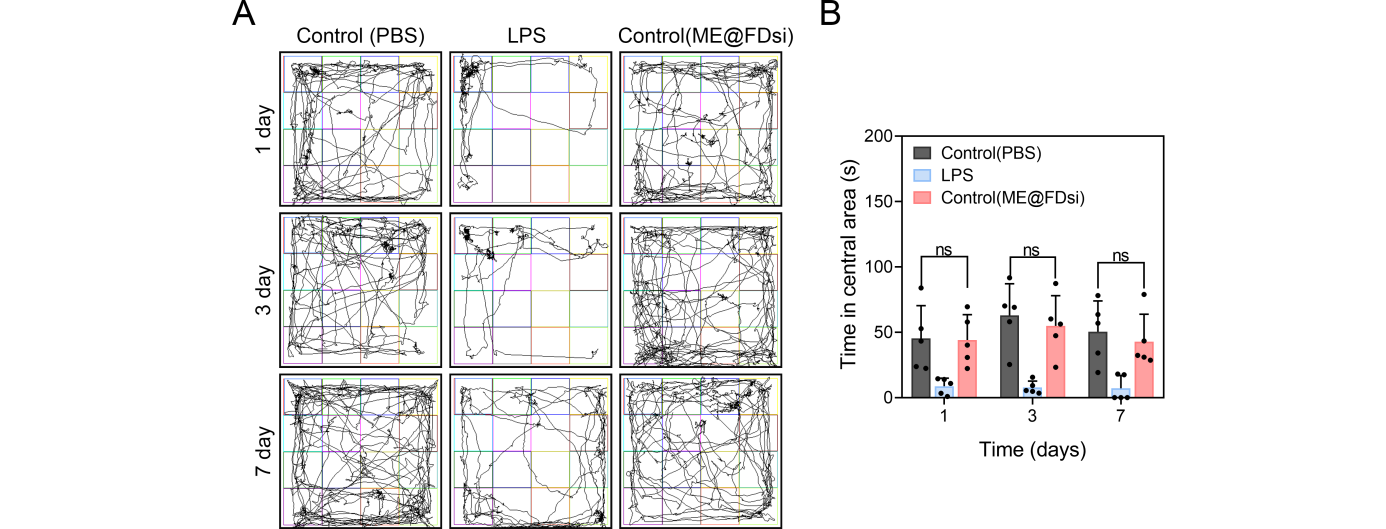
**

**Figure S44. Behavioral safety assessment of ME@FDsi in healthy mice. Healthy mice were divided into three groups: the Control (PBS) group (healthy control, injected with PBS *via* the tail vein), the LPS group (SAE model positive control, intraperitoneally injected with 10 mg/kg LPS), and the Control (ME@FDsi) group (healthy mice injected with ME@FDsi *via* the tail vein). Open field tests were performed on days 1, 3, and 7 post-administration to assess the time spent in the center zone. Data are presented as mean ± standard deviation. Statistical analysis was performed using one-way ANOVA. **p* < 0.05, ***p* < 0.01, ****p* < 0.001, *n.s.* indicates no significant difference.**

**
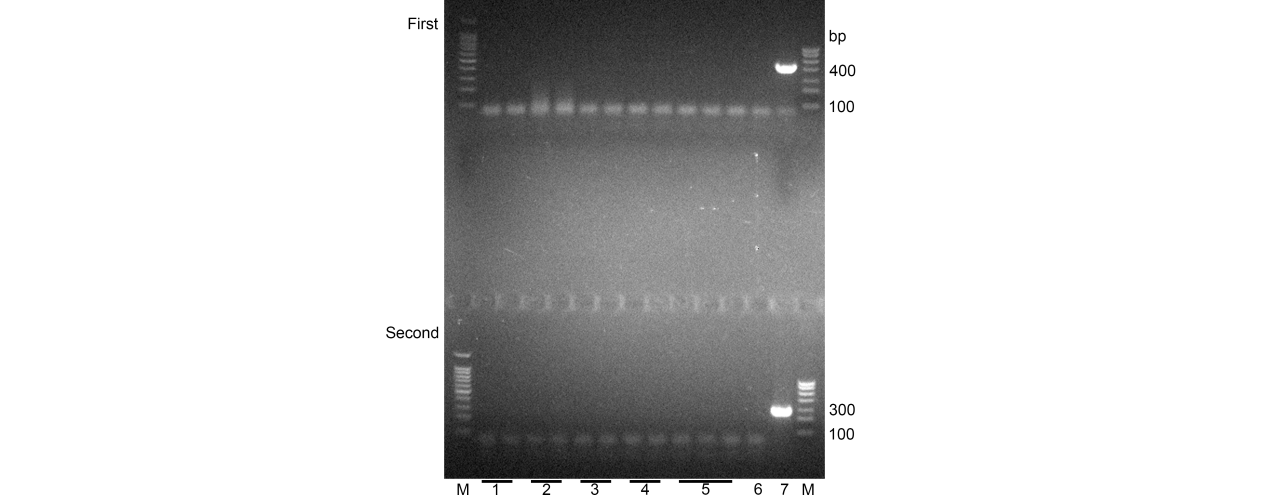
**

**Figure S45.**Mycoplasma Testing Results of Different Cell Lines. M, Marker; Lane 1, BV2 cells; Lane 2, bEnd.3 cells; Lane 3, Raw 264.7 cells; Lane 4, HUVEC cells; Lane 5, Water in Different Incubators; Lane 6, Negative Control; Lane 7, Positive Control. The PCR product sizes for the positive controls were 448 bp in the first round and 304 bp in the second round.
